# Analysis of immune cell infiltration characteristics in severe acute pancreatitis through integrated bioinformatics

**DOI:** 10.1038/s41598-024-59205-1

**Published:** 2024-04-15

**Authors:** Shuai Xiao, Xiao Han, Shuhui Bai, Rui Chen

**Affiliations:** 1 Department of Intensive Care Medicine, Tengzhou Central People’s Hospital, Tengzhou, China; 2 Department of General Practice, The Third People’s Hospital of Chengdu, Chengdu, China; 3 Department of Nutriology, The Affiliated Hospital of Xuzhou Medical University, Xuzhou, China; 4 Department of General Practice, Jining First People’s Hospital, Jining, China

**Keywords:** Biological techniques, Computational biology and bioinformatics, Gastroenterology

## Abstract

The etiopathogenesis of severe acute pancreatitis (SAP) remains poorly understood. We aim to investigate the role of immune cells Infiltration Characteristics during SAP progression. Gene expression profiles of the GSE194331 dataset were retrieved from the GEO. Lasso regression and random forest algorithms were employed to select feature genes from genes related to SAP progression and immune responses. CIBERSORT was utilized to estimate differences in immune cell types and proportions and the relationship between immune cells and gene expression. We performed pathway enrichment analysis using GSEA to examine disparities in KEGG signaling pathways when comparing the two groups. Additionally, CMap analysis was executed to identify prospective small molecular compounds. The three hub genes (CBLB, JADE2, RNF144A) were identified that can predict SAP progression. Analysis of CIBERSORT and TISIDB databases has shown that there are significant differences in immune cell expression levels between the normal and SAP groups, and three hub genes (CBLB, JADE2, RNF144A) were highly correlated with multiple immune cells, regulating the characteristics of immune cell infiltration in the microenvironment. Finally, drug prediction through the Connectivity Map database suggested that compounds such as Entecavir, KU-0063794, Y-27632, and Antipyrine have certain effects as potential targeted drugs for the treatment of SAP. CBLB, JADE2, and RNF144A are hub genes in SAP, potentially playing important roles in SAP progression. This finding further broadens the understanding of the etiopathogenesis of SAP and provides a feasible basis for future research on diagnostic and immunotherapeutic targets for SAP.

## Introduction

Severe acute pancreatitis (SAP) is a prevalent clinical emergency case marked by extensive necrosis in the pancreas and the surrounding tissues. This condition swiftly advances from localized inflammation to trigger systemic inflammatory response syndrome (SIRS), resulting in harm to vital organs, ultimately leading to the development of multiple organ dysfunction syndrome (MODS). It is reported that the mortality rate of SAP is as high as 15–35%^1,2^. At present, the clinical treatment of SAP mainly focuses on supportive and non-specific measures. Recent treatment recommendations include targeted intravenous fluid resuscitation, appropriate sedation and analgesic management, timely enteral nutrition when necessary, avoidance of prophylactic use of antibiotics, and ERCP in patients diagnosed with acute biliary pancreatitis^3^. The etiology and pathogenesis of SAP are still unclear, although existing researches suggest that immune cells. At present, the clinical treatment of SAP mainly focuses on supportive and non-specific measures. Recent treatment recommendations include targeted intravenous fluid resuscitation, appropriate sedation and analgesic management, timely enteral nutrition when necessary, avoidance of prophylactic use of antibiotics, and ERCP in patients diagnosed with acute biliary pancreatitis^4^.

In the initial stages of acute pancreatitis, immune cells become activated, releasing cytokines and inflammatory mediators. This activation sets off a chain of coagulation reactions, ultimately resulting in SIRS. The body responds by amplifying both inflammatory and anti-inflammatory processes, contributing to the development of CARS. However, as the disease progresses, an excessive immune response or decreased immune function increases the risk of death in patients with SAP^5^. Therefore, balancing the pro-inflammatory and anti-inflammatory processes through immune regulation is key to improving SAP prognosis.

With the widespread application of high-throughput biological techniques in biomedical research, microarray data analysis has been extensively utilized to explore new critical genes associated with disease mechanisms. Prior bioinformatics studies have shown that immune cell infiltration and immunological-related pathways are involved in SAP^6^, highlighting the pivotal role of the immune mechanisms in SAP. This study applies biological techniques to analyze hub genes and immune cell infiltration differences in SAP, aiming to further provide a theoretical basis for its early diagnosis and treatment.

## Materials

### Data download

#### The GEO database

The dataset (GSE194331) was downloaded from the GEO database. And GSE194331 included gene expression profiles of 42 patients, selecting 10 SAP samples and 32 normal samples.

### Differential expression analysis

The Limma package can identify differentially expressed genes between different groups and analyze the molecular mechanisms of SAP data. Differentially expressed genes are selected based on the criteria of P < 0.05 and |logFC|> 1 to generate volcanic and heat maps.

### GO and KEGG enrichment analyses

GO and KEGG were used to evaluate the relevant functional categories in detail. Significance was assigned when the p-value and q-value < 0.05 of the enriched pathway corresponding to GO and KEGG.

### Lasso regression and random forest

We used Lasso regression and random forest algorithms to select diagnostic markers for SAP. The "glmnet" package was used for the Lasso algorithm. Random samples are selected with replacement to create decision trees. The importance of features was assessed using the random forest algorithm, evaluating feature importance based on %IncMSE. The top 5 features were selected for subsequent study.

### Immune cell infiltration analysis

The CIBERSORT method involves the use of support vector regression principles to decompose the expression matrix of immune cell subsets. In this study, the CIBERSORT algorithm was used to calculate the relative proportions of various immune cells and perform Spearman correlation analysis to investigate their relationship with gene expression levels.

### GSEA pathway enrichment

GSEA ranks genes based on differential expression between two types of samples, and then tests for enrichment of the predefined gene set. GSEA was performed to compare KEGG pathway differences between two different expression groups,obtaining the molecular mechanisms of hub genes in two patient populations.

### CMap drug prediction

CMap analysis was executed to identify prospective small molecular compounds.

### Statistical analysis

All statistical analyses were carried out using R software 4.2.2. All statistical tests were two-sided, and p < 0.05 was considered significant**.**

## Results

**Figure 1:**
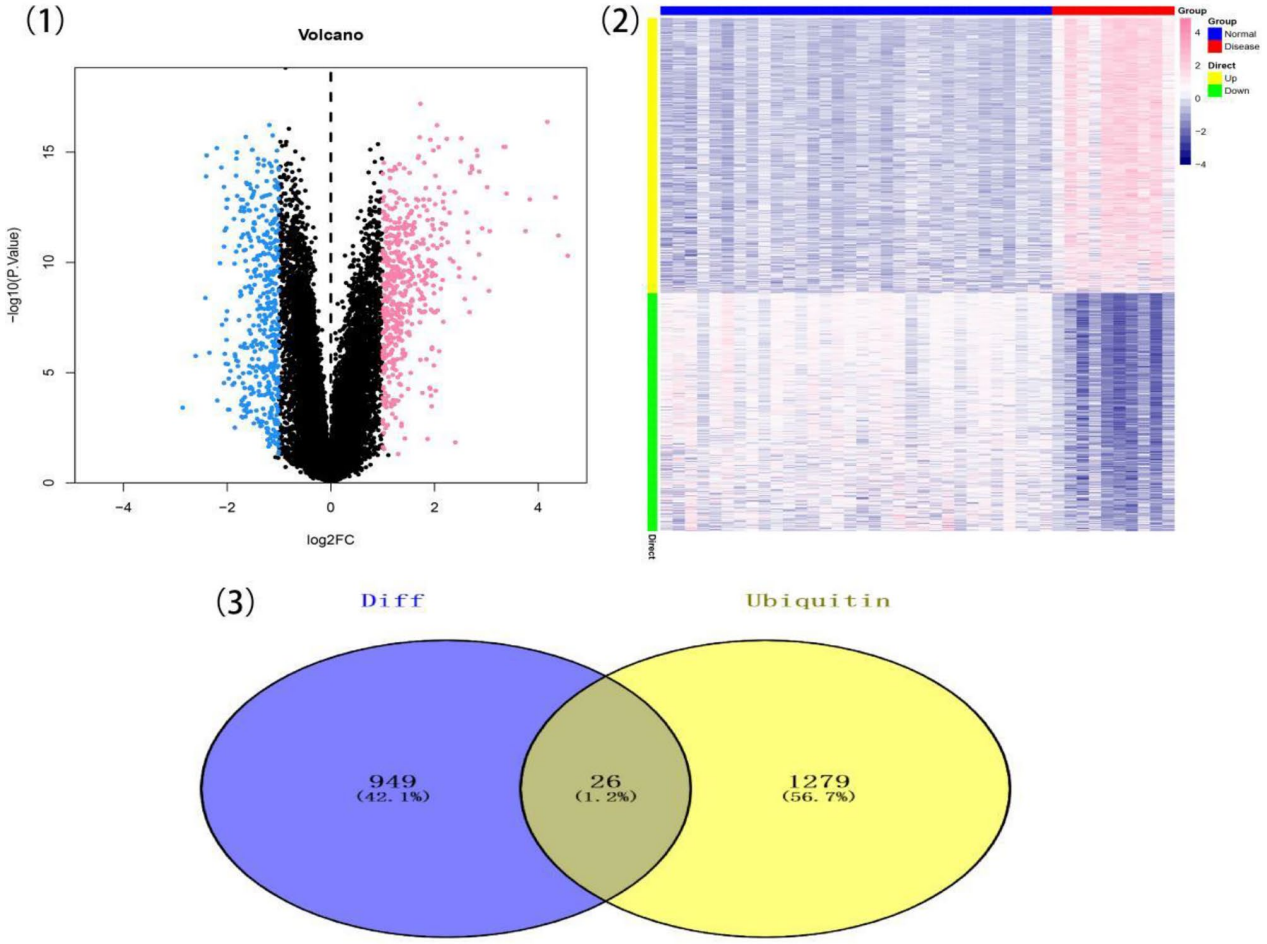
Identification of DEGs between control group and disease group. (1) Volcano plot showing the DEGs between control and disease groups after analysis of the GSE194331 dataset with R software. (2) A heatmap showing the DEGs between the two groups. (3) Extract ubiquitination related genes with Relevance score > 5 and intersect with differential genes to obtain 26 intersecting genes.

**Figure 2:**
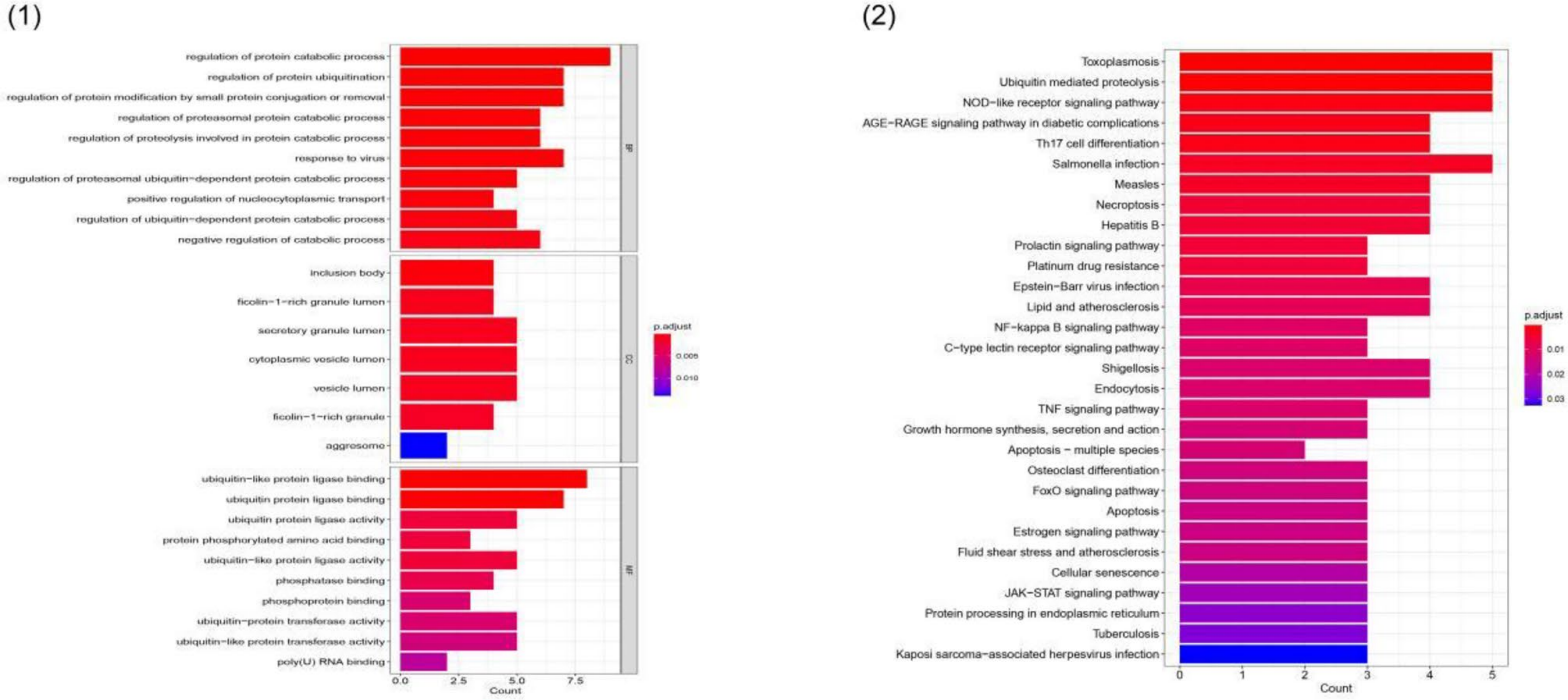
Biofunctional enrichment analysis of DEGs. (1) GO functional enrichment analyses of DEGs. (2) KEGG pathway enrichment analyses of DEGs.

**Figure 3:**
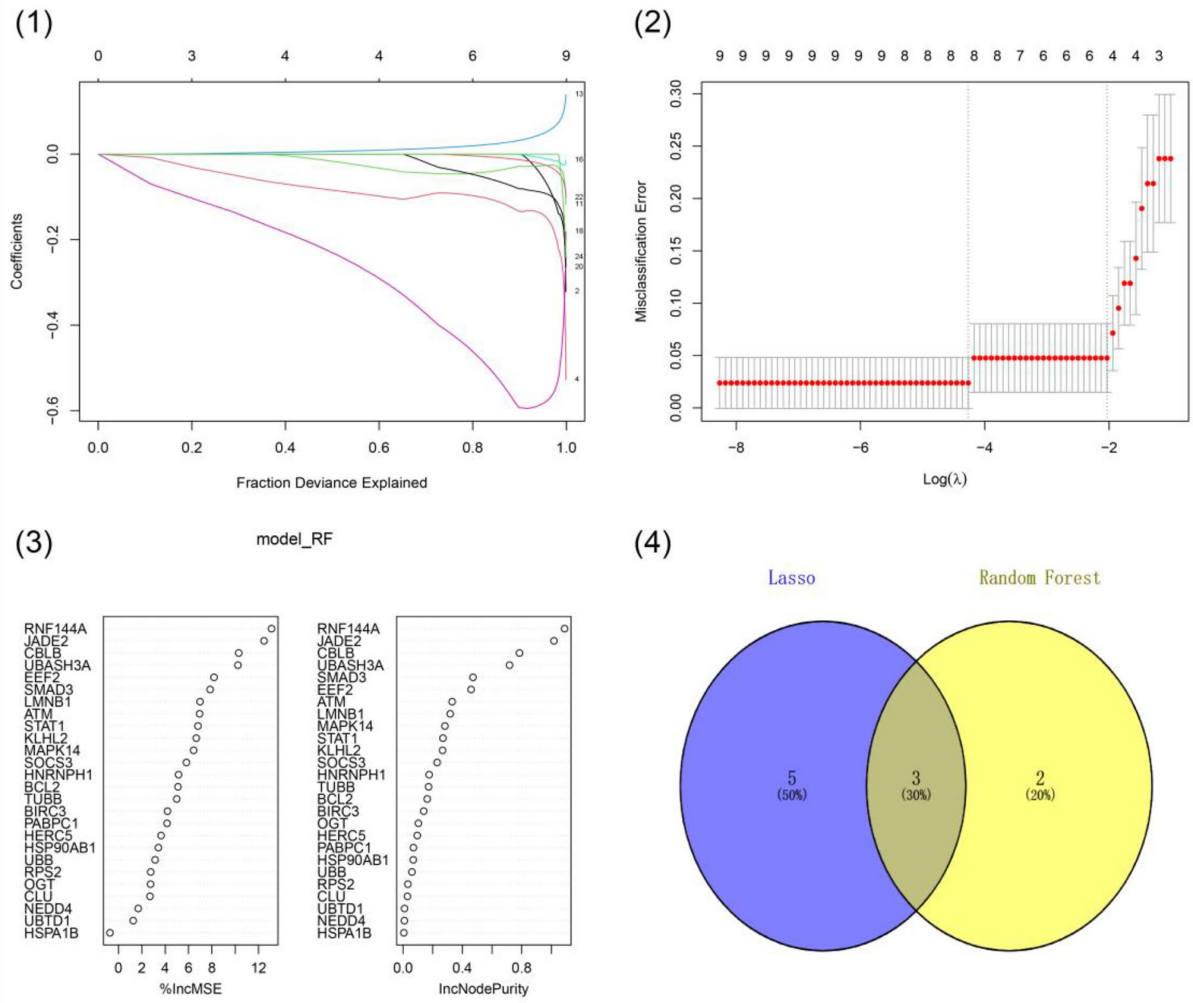
Identification of key genes influencing severe pancreatitis. (1–2) Lasso Regression identifies 8 characteristic genes of severe pancreatitis. (3) Top 5 characteristic genes of severe pancreatitis screened by Random Forest. (4) 3 intersecting genes were screened by Lasso Regression and Random Forest.

**Figure 4:**
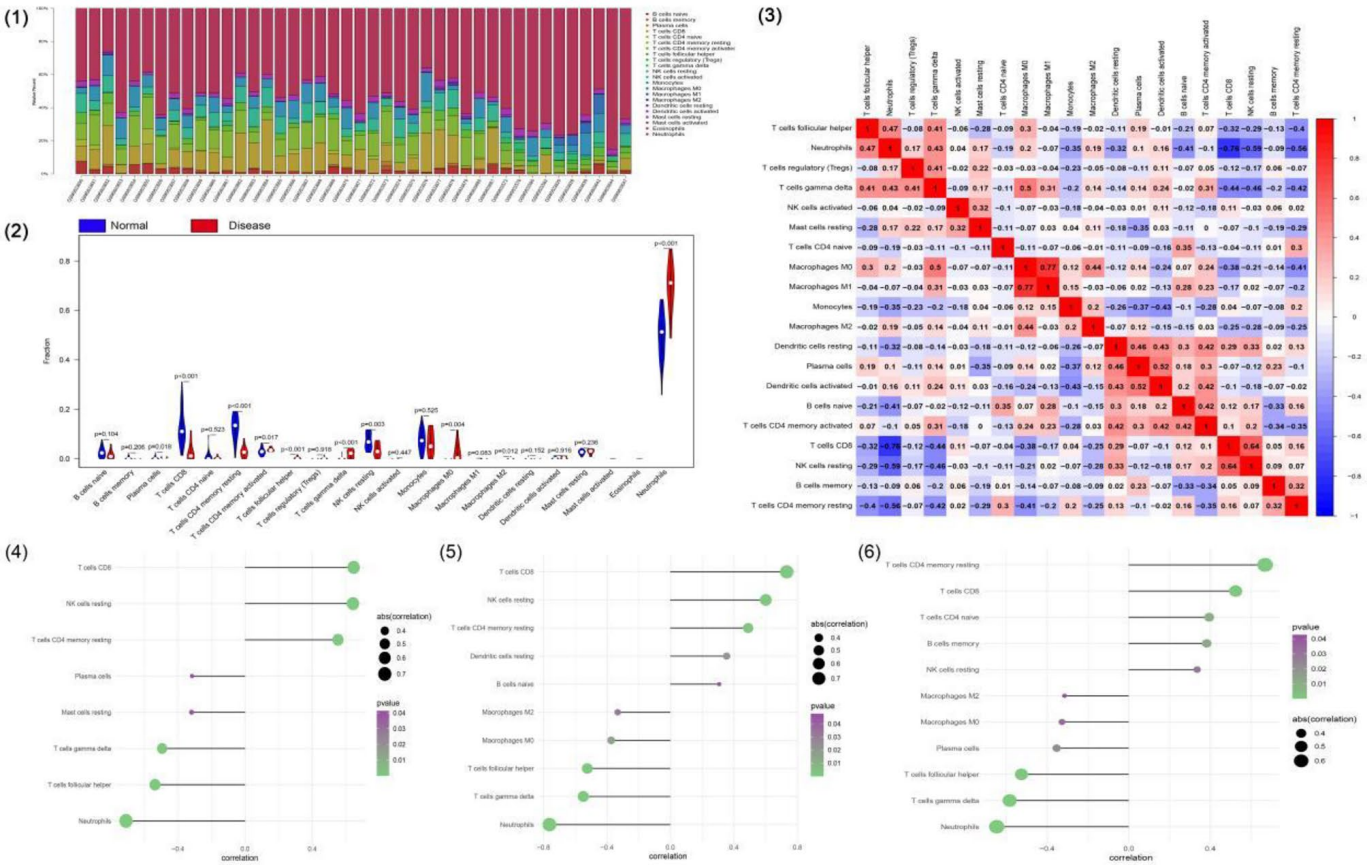
Immune cell infiltration patterns in control group and disease group. (1) Histogram of the proportions of 22 immune cell subpopulations. x-axis: GEO samples; y-axis: percentage of each immune cell type. (2) Violin plot showing the differentially infiltrated immune cells between the two groups. Blue represents the control group and red represents the disease group. (3) Correlation heatmap of all immune cells. Numbers in the small square represent Pearson’s correlation coefficient between the two immune cells on the horizontal and vertical coordinates; red squares indicate positive correlation, and blue squares indicate negative correlation. (4) The correlation between the hub gene and the immune cell of CBLB. (5) The correlation between the hub gene and the immune cell of JADE2. (6) The correlation between the hub gene and the immune cell of RNF144A. The size of the dots represents the strength of the correlation between genes and immune cells, and the color of the dots represents the p-value. p < 0.05 was considered statistically significant.

**Figure 5:**
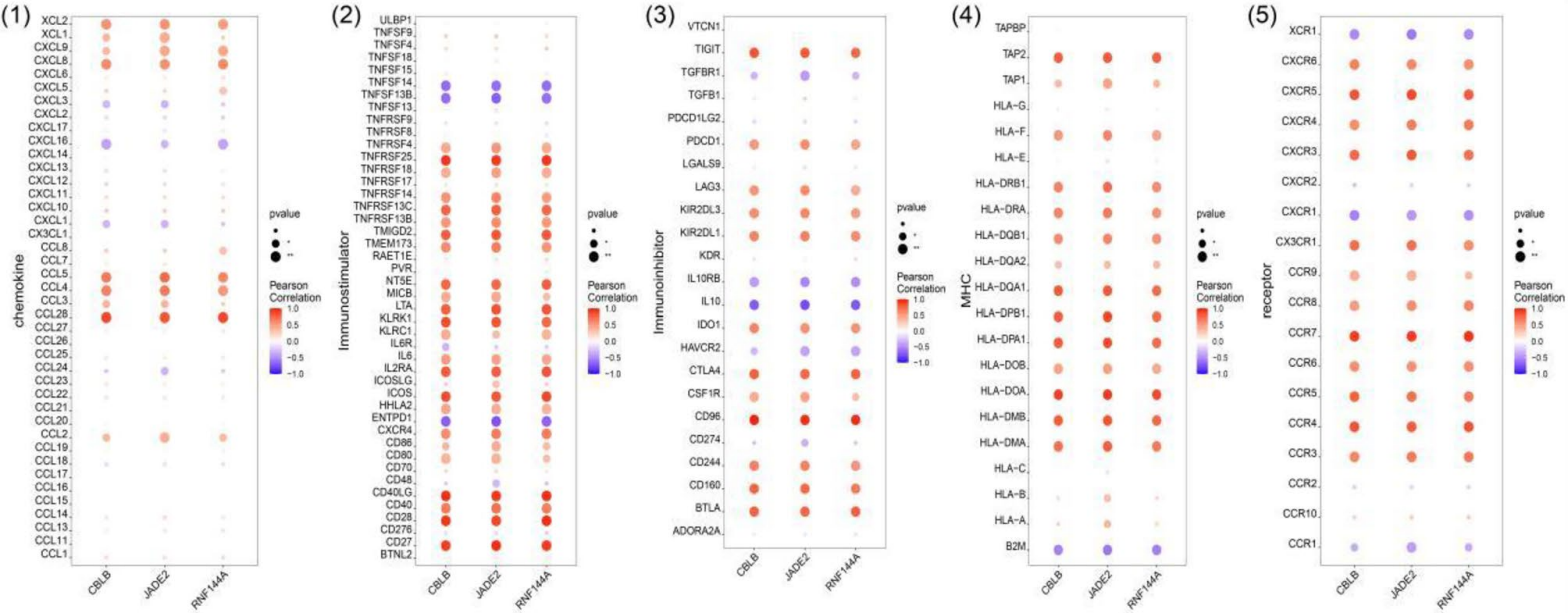
The correlation between the hub gene and the immune cell. (1) Chemokine. (2) Immunostimulator. (3) Immunoinhibitor. (4) MHC. (5) Receptor. *p < 0.05; **p < 0.01; and ***p < 0.001.

**Figure 6:**
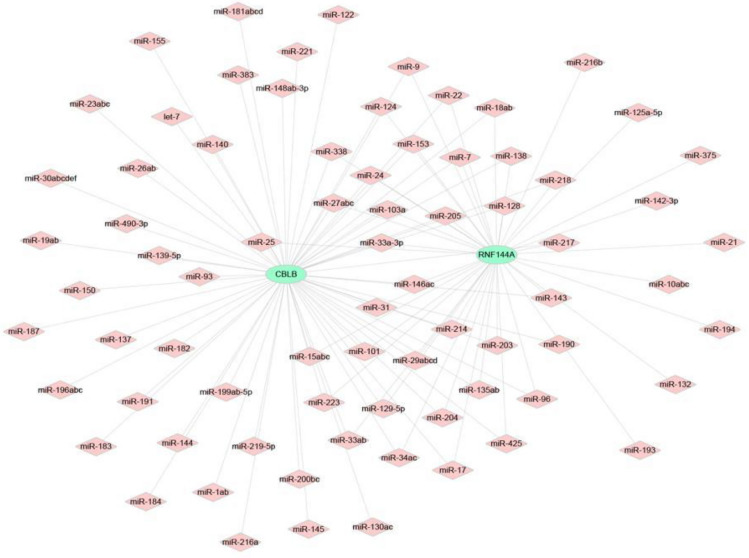
Interaction network of mRNA-miRNA.

**Figure 7:**
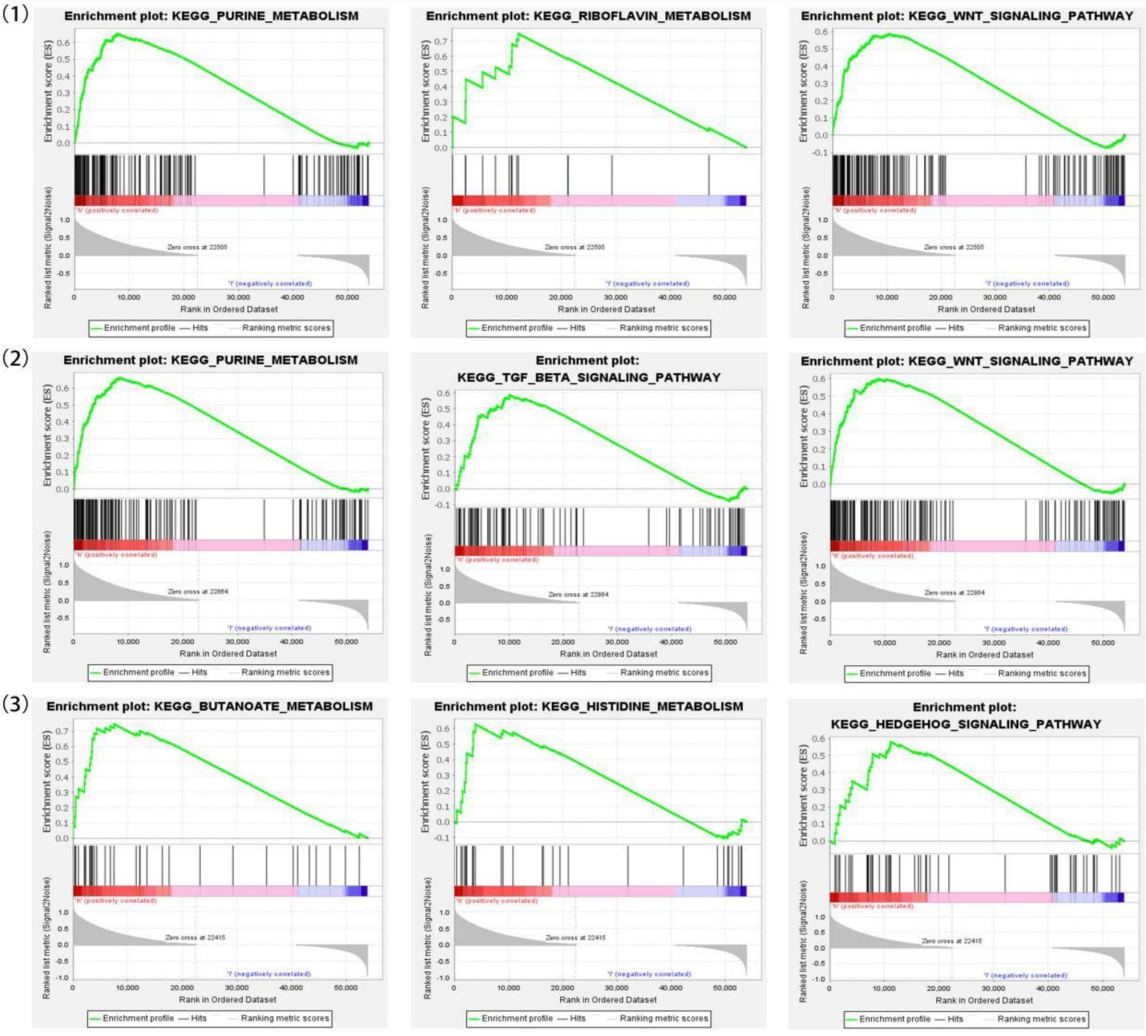
The GSEA of hub gene. (1) CBLB. (2) JADE2. (3) RNF144A.

**Figure 8:**
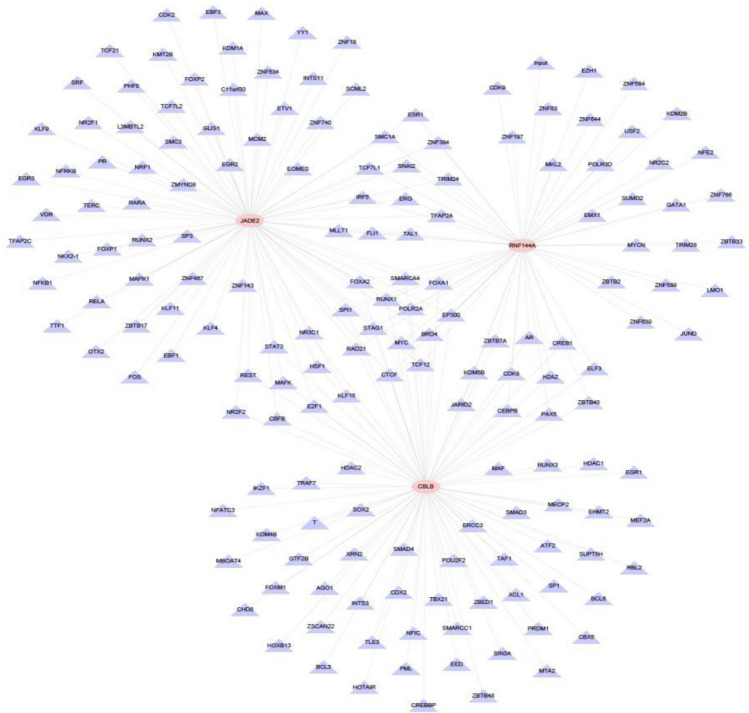
Cistrome analysis.

**Figure 9:**
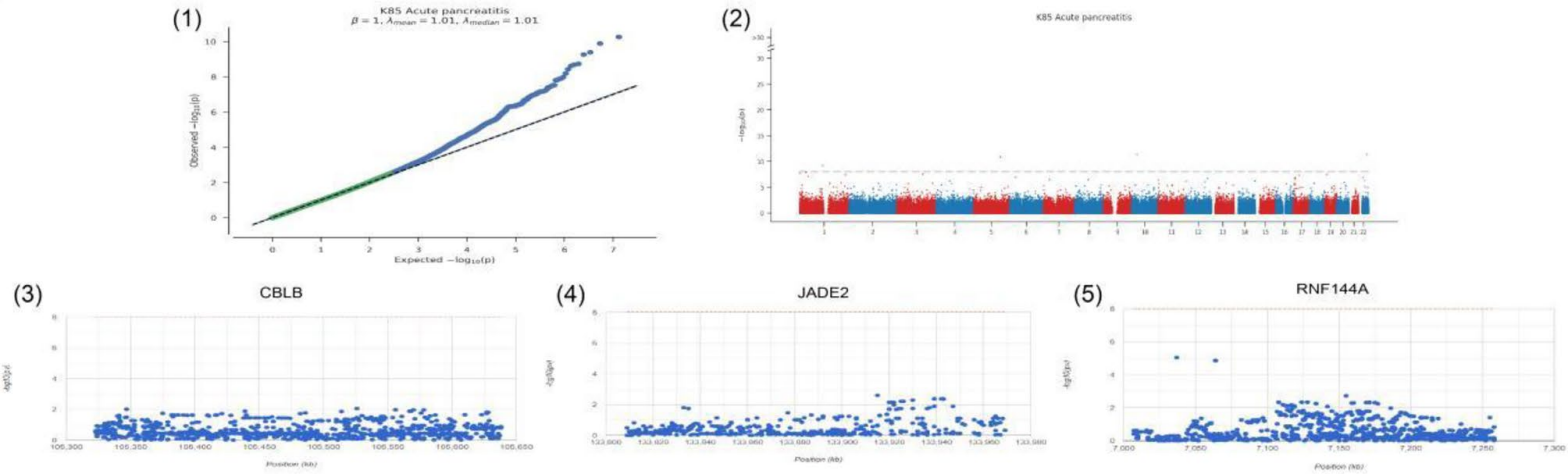
GWAS analysis. (1) Disease-associated significant SNP loci identified. (2) Description of key SNP loci distributed in the enriched region. (3–5) Demonstration of SNP pathogenic regions corresponding to CBLB, JADE2, RNF144A.

**Figure 10:**
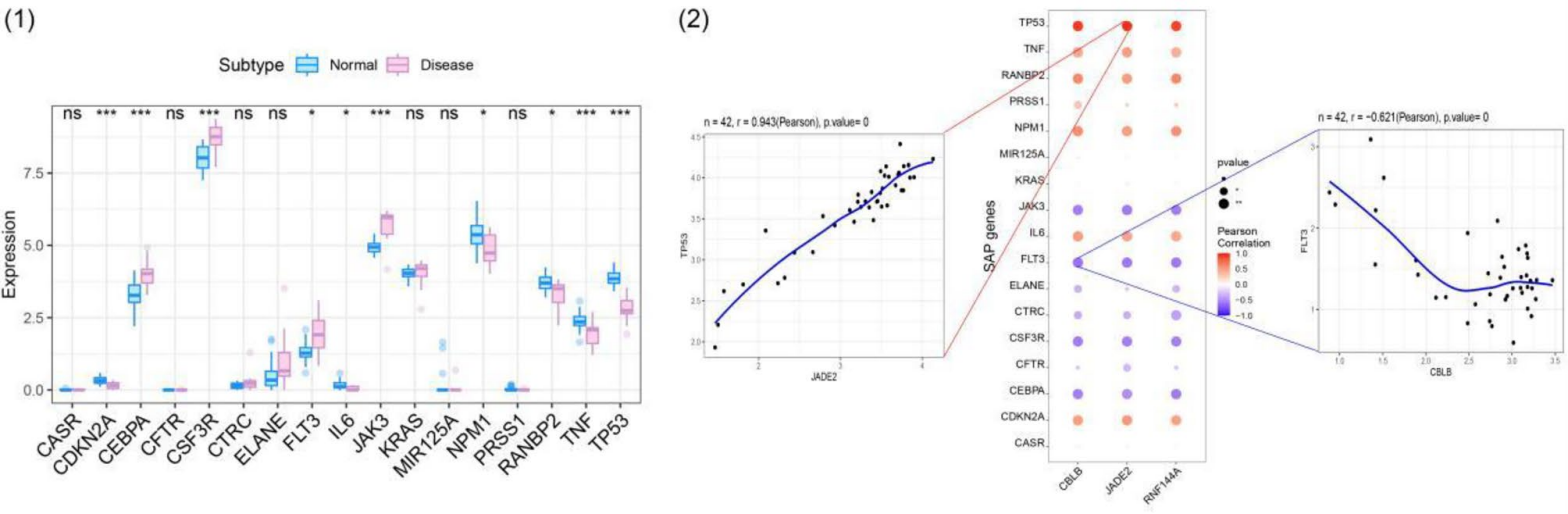
Correlation with severe acute pancreatitis. (1) Diff expression. (2) Correlation analysis. *p < 0.05; **p < 0.01; and ***p < 0.001.

**Figure 11:**
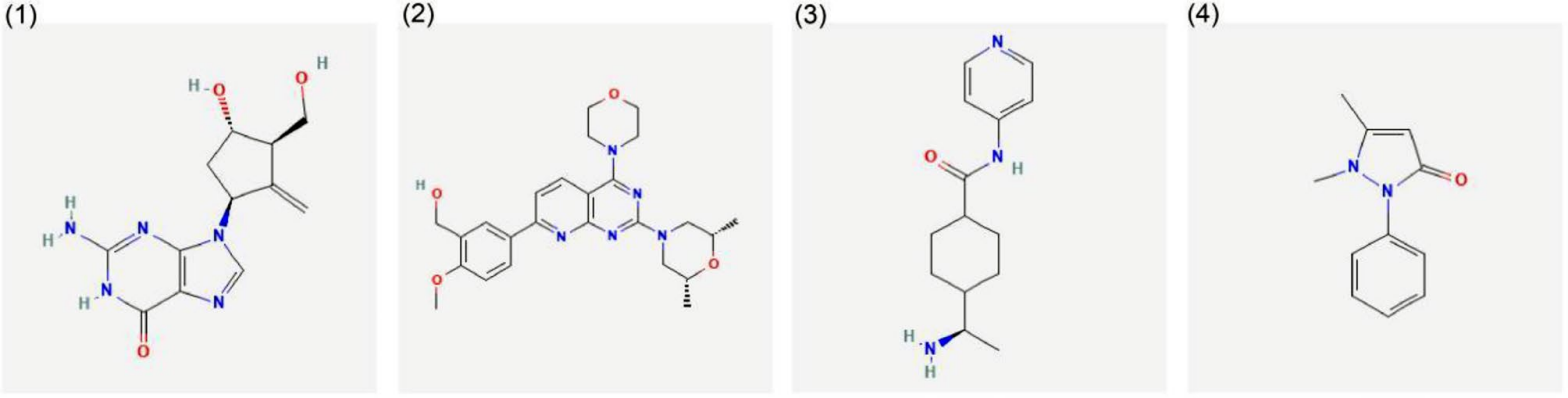
Drug predication. (1) Entecavir. (2) KU-0063794. (3) Y-27632. (4) Antipyrine.

1. From the GEO database, we downloaded the GSE194331 dataset related to SAP, including a control group (n = 32) and a disease group (n = 10). Differential genes between control and disease groups were calculated using limma software package,with the screening criteria of P-value < 0.05 and |logFC|> 1, and identified a total of 975 differential genes (Fig. 1(1,2)), including 523 upregulated and 452 downregulated. Subsequently, we extracted ubiquitin-related genes with Relevance score > 5 from the GeneCards database and intersected them with the differential genes, resulting in 26 overlapping genes (Fig. 1(3)). We further performed pathway analysis on the overlapping genes. The GO enrichment analysis showed that the overlapping genes were mainly enriched in pathways such as regulation of protein ubiquitination and others (Fig. 2(1)). The KEGG enrichment analysis showed that the overlapping genes were mainly enriched in pathways such as Ubiquitin-mediated proteolysis and others (Fig. 2(2)).
2. To identify hub genes influencing SAP, we used lasso regression and random forest methods, utilizing the intersecting genes obtained in the previous step. The results from Lasso regression identified 8 genes as feature genes for SAP (Fig. 3(1,2)). Additionally, we utilized the random forest algorithm to select feature genes, considering the top 5 ranked genes as feature genes for SAP (Fig. 3(3)). We then took the intersection of these feature genes with those obtained from the Lasso regression algorithm, resulting in 3 intersecting genes (Fig. 3(4)). CBLB, JADE2, and RNF144A were designated as hub genes for our subsequent research.
3. We analyzed the SAP dataset to obtain the immune cells ratios and the correlation immune cells among different patients (Fig. 4(1,2)). Comparing the immune cell levels between the two groups, we found significant differences in T cell CD8, T cell CD4 memory resting, etc. (Fig. 4(3)). Next, we performed Spearman correlation analysis and revealed that the three hub genes, CBLB, JADE2, and RNF144A, were highly correlated with multiple immune cells(Fig. 4(4–6)). Additionally, we obtained from the TISIDB database the correlation between hub genes and immune suppressive factors, immune stimulatory factors, chemotactic factors, and receptors among different immune factors (Fig. 5). These analyses suggest that hub genes may regulate the immune cell infiltration characteristics in the microenvironment during SAP progression.
4. We used the mircode database for reverse prediction of hub genes,and obtained 108 mRNA-miRNA relationship pairs (Table S2). Then, we utilized Cytoscape to visualize these relationships(Fig. 6).GSEA analysis was used to compare the differences in signaling pathway expression between the two groups for CBLB, JADE2, and RNF144A genes, revealing the unknown mechanisms of SAP progression.CBLB enrichment was observed in pathways such as PURINE METABOLISM, RIBOFLAVIN METABOLISM, and WNT SIGNALING PATHWAY (Fig. 7(1)). JADE2 enrichment was found in pathways like TGF BETA SIGNALING PATHWAY, PURINE METABOLISM, and KEGG WNT SIGNALING PATHWAY (Fig. 7(2)). RNF144A enrichment was observed in pathways like BUTANOATE METABOLISM, HISTIDINE METABOLISM, and HEDGEHOG SIGNALING PATHWAY (Fig. 7(3)).
5. Using the Cistrome database, we obtained a predicted list of transcription factors for each of these threes genes to reveal the transcriptional regulatory networks involved in these hub genes. We predicted that CBLB is regulated by 84 transcription factors, JADE2 by 86 transcription factors, and RNF144A by 62 transcription factors. These predicted transcription factors can serve as backup candidates for further investigation of the transcriptional regulatory networks of these three hub genes. We then used Cytoscape to visualize the transcriptional regulatory network of these three hub genes (Fig. 8). Next, GWAS data was analyzed of the three hub genes to identify the regions related to SAP. The Q-Q plot displayed the significant disease-related SNP loci identified through GWAS data (Fig. 9). Through accurate localization of the GWAS data, the precise localization of key SNP sites enriched in the disease-related regions is described.We presented the disease-associated regions corresponding to CBLB, JADE2, and RNF144A, where CBLB is located on chromosome 3, JADE2 on chromosome 5, and RNF144A on chromosome 2. Significant SNP loci matching to the three genes are listed in the table (GWAS data.xlsx).
6. We obtained genes associated with SAP from https://www.genecards.org/. By calculating the top 20 genes expressed level based on correlation scores (Table S1), we observed intergroup differences in these SAP-related genes expression,including CDKN2A, CEBPA, CSF3R, FLT3, IL6, JAK3, NPM1, RANBP2, TNF, TP53, and others (Fig. 10(1)). In addition, we also observed close link between three hub genes expressed level and multiple genes associated with SAP (Fig. 10(2)). And it is worth noting that there is a remarkably positive correlation between JADE2 and TP53 (Pearson correlation coefficient of 0.943), while there is a remarkably negative correlation between CBLB and FLT3 (Pearson correlation coefficient of − 0.621).
7. We divided the top 150 up- and downregulated genes into two groups and conducted drug predictions using CMap, and the results indicated that the expression profiles of drugs-perturbed such as Entecavir, KU-0063794, Y-27632, and Antipyrine were significantly negatively correlated with the expression profiles of disease-perturbed, suggesting that these drugs can improve or even stop SAP progress (Fig. 11).

## Discussion

Through studying the epidemiological development trends of pancreatitis in recent years, we can find that the incidence of acute pancreatitis is constantly increasing. Approximately 20% of patients with AP will progress to moderate or severe pancreatitis. SAP, characterised by high mortality rate, aggressive progression, and diverse complications, which makes it one of the major public health problems globally^7^. Therefore, early and accurate identification of the severity of SAP is crucial for patient rescue. As early as 1974,s using CMap, and the results indicated that the expression profiles of drugs-perturbed such as Entecavir, KU-0063794, Y-27632, and Antipyrine were significantly negatively correlated with the expression profiles of disease-perturbed, suggesting that these drugs can improve or even stop SAP progress^8^. Subsequently, numerous scoring systems based on clinical, imaging, and laboratory indicators, such as Glasgow^9^, APACHE II^10^, APACHE II^11^, APACHE II^12^, were developed to evaluate AP severity. While existing scoring systems have played a significant role in predicting sustained organ failure in AP, they have limitations, such as requiring multiple statistical parameters, being computationally complex, having a broad time span for required indicators, and exhibiting some lag in assessing disease severity. Therefore, due to the high misdiagnosis rate of severe pancreatitis, which can reach up to 20–30%, there is an urgent need to identify reliable biomarkers to effectively detect the development of SAP.

The immunomodulation of pro-inflammatory and anti-inflammatory processes during the course of AP has been a focus of basic research in recent years. As everyone knows, pancreatitis is primarily considered a sterile inflammatory condition. Early in the disease, a large amount of pancreatic enzymes are activated, leading to the necrosis of acinar cells and the production of various cytokines and inflammatory mediators^13^. As a defence mechanism firstly, it leads to a significant increase in immune-infiltrating cells, such as macrophages, neutrophils, T cells, dendritic cells, B cells, and mast cells, which can help promote disease recovery^14^. As pathogenic factors persist, immune cell-related inflammatory reactions continue and amplify. At the same time, these inflammatory cells produce cytokines and chemokines, which recruit more inflammatory cells to aggregate, activate the cascade of inflammatory factors, and exacerbate pancreatic damage This process can continue and cause damage to vital organs such as the liver, lungs, resulting in MODS (multi-organ dysfunction syndrome)^15^. Through the secretion of pro-inflammatory mediators. This uncontrolled inflammatory response is often accompanied by CARS, related to immune suppression. At the same time, these inflammatory cells produce cytokines and chemokines, which recruit more inflammatory cells to aggregate, activate the cascade of inflammatory factors, and exacerbate pancreatic damage This process can continue and cause damage to vital organs such as the liver, lungs, resulting in MODS (multi-organ dysfunction syndrome) creatic necrosis^5^. To explore the imbalance of inflammatory cells in SAP, we conduct an immune infiltration analysis and find significantly elevated levels of immune cell types such as T cell CD8,T cell CD4 memory resting, etc. in SAP patients compared to controls. This is consistent with previous studies^16–21^. The above evidence suggests that cellular immune infiltration is closely related to SAP progression.

In addition, in our study of the relationship between hub genes and immune cells, we find that the three hub genes, CBLB, JADE2, and RNF144A, are highly correlated with multiple immune cells. Using lasso regression and random forest, we ultimately identified three hub genes (CBLB, JADE2, RNF144A) in SAP. These genes are significant discoveries since they have never been linked to SAP progression before. The immune system cells feature a significant expression of CBL-B, which is necessary for its regulatory function. CBL-B exerts a significant influence on the inhibition of peripheral T cell tolerance and autoimmune diseases by promoting the ubiquitination and degradation of receptor internalization signaling proteins^22^. CBL-B is involved in the TCR-induced NF-κB activation process, which is primarily regulated in primary T cells through Akt-dependent and PKC-θ-dependent pathways^23^. By knocking out the CBLB gene, the inhibitory effect of the TCR-CD28 co-stimulation pathway can be alleviated, thereby activating the PI3K and NF-κB signaling pathways, disrupting the inhibition of the T cell negative feedback mechanism, and ultimately xacerbate pancreatic tissue damage^22^. JADE2 (jade family PHD finger 2) is a member of the JADE family, also known as PHF15. Little is known about JADE2 currently. Our study proposes that JADE2 may participate in SAP progression through pathways such as TGF BETA SIGNALING PATHWAY, PURINE METABOLISM, and KEGG WNT SIGNALING PATHWAY, highlighting the need for further foundational research on JADE2's biological mechanisms.RNF144A is a ligase that catalyzes the transfer of ubiquitin to substrates and is involved in a variety of cellular processes including apoptosis and innate immunity. Shiuh-Rong Ho's study analyzed the BioGPS database and found RNF144A to be specifically expressed in pancreatic islet cells. RNF144A can suppress the pro-survival function of DNA-PKcs through the ubiquitin–proteasome system, inducing apoptosis in cells with sustained or severe DNA damage^24^. Our research indicates that RNF144A is enriched in pathways such as BUTANOATE METABOLISM, HISTIDINE METABOLISM, and HEDGEHOG SIGNALING PATHWAY, suggesting the involvement of multiple mechanisms and pathways in SAP progression. To further understand the mechanism of action of the hub genes identified in the previous step, we conducted correlation analysis between these genes and immune factors including immunosuppressive factors, immune stimulatory factors, chemokines, and receptors. The results of this analysis will help us better understand the role of these hub genes in immune regulation and tumorigenesis. These analysis results indicate that hub genes are closely associated with immune cell infiltration levels and play an important role in the immune microenvironment, but further validation of these findings is required in clinical patients and subsequent animal experiments to support their application in clinical practice.

The pathogenesis of AP is complex, with alcohol consumption, biliary tract disease, and high triglyceride lipids being the three most common etiologic factors. In addition, older, gender, degree of localized pancreatic injury, and genetic susceptibility can also contribute to AP^25,26^. All of these risk factors have the potential to develop into severe if not effectively managed at an early stage. Among these, older is a non-negligible etiologic factor contributing to SAP, and early recognition and timely action are essential to improve outcomes in this population^27^. We studied the role of immune cells infiltration characteristics during severe acute pancreatitis (SAP) progression using gene expression profiles of the GSE194331 dataset from the GEO, and Lasso regression and random forest algorithms with selecting feature genes from genes related to SAP progression and immune responses. However, due to the lack of relevant clinical data in the dataset (e.g., age, gender, etiology of underlying disease, etc.), we did not separately analyze the occurrence of SAP due to different etiologies. Therefore, subsequent studies can be conducted around this concern to further refine the pathomechanisms associated with SAP.

## Conclusion

In this study, chip analysis is used to identify the hub genes and pathways closely related to SAP. In addition, we describe in detail the underlying immune infiltration pattern in SAP (Supplementary Information).

### Supplementary Information

Supplementary Information.

## Data Availability

All data can be found in the GEO database. https://www.ncbi.nlm.nih.gov/geo/query/acc.cgi?acc=GSE194331. Further information can be obtained by contacting the corresponding author.
