# Supplementary material for: Analysis of immune cell infiltration characteristics in severe acute pancreatitis through integrated bioinformatics
Source: Sci Rep. 2024 Apr 15;14:8711. doi: 10.1038/s41598-024-59205-1 (PMC11018854; doi:10.1038/s41598-024-59205-1)
Supplement: Supplementary file 1 — Supplementary Information. [file 41598_2024_59205_MOESM1_ESM.docx]

table S1. Drug correlation scores by calculating the top 20 genes expressed levels

| Rank | Score | Type | ID | Name | Description |
| --- | --- | --- | --- | --- | --- |
| 8540 | -97.25 | cp | BRD-K08206212 | entecavir | Reverse transcriptase inhibitor |
| 8534 | -96.42 | cp | BRD-K67566344 | KU-0063794 | MTOR inhibitor |
| 8525 | -94.4 | cp | BRD-K44084986 | Y-27632 | Rho associated kinase inhibitor |
| 8518 | -93.56 | cp | BRD-K46937689 | phenazone | Cyclooxygenase inhibitor |
| 8514 | -93.06 | cp | BRD-K23192422 | lestaurtinib | FLT3 inhibitor |
| 8510 | -91.97 | cp | BRD-A26002865 | verrucarin-a | Protein synthesis inhibitor |
| 8503 | -91.25 | cp | BRD-K90382497 | GW-843682X | PLK inhibitor |
| 8496 | -90.78 | cp | BRD-A25687296 | emetine | Protein synthesis inhibitor |
| 8487 | -89.77 | cp | BRD-K69932463 | AZD-8055 | MTOR inhibitor |
| 8484 | -89.61 | cp | BRD-A53077924 | tianeptine | Selective serotonin reuptake enhancer (SSRE) |
| 8473 | -88.73 | cp | BRD-K76674262 | homoharringtonine | Protein synthesis inhibitor |
| 8468 | -88.2 | cp | BRD-K80348542 | cephaeline | Protein synthesis inhibitor |
| 8462 | -87.07 | cp | BRD-K05434375 | HA-1004 | Calcium channel blocker |
| 8461 | -86.88 | cp | BRD-A83695761 | chromanol | Potassium channel blocker |
| 8447 | -85.03 | cp | BRD-K69688083 | mestinon | Cholinesterase inhibitor |
| 8420 | -82.74 | cp | BRD-K83837640 | JNJ-26854165 | HDAC inhibitor |
| 8416 | -82.59 | cp | BRD-K20995441 | U-54494A | Opioid receptor agonist |
| 8411 | -81.87 | cp | BRD-K27871032 | lysergol | Ergoline alkaloid |
| 8394 | -79.89 | cp | BRD-K55930204 | phenytoin | Hydantoin antiepileptic |
| 8392 | -79.8 | cp | BRD-A45664787 | iloprost | Platelet aggregation inhibitor |
| 8390 | -79.69 | cp | BRD-K70577657 | H-9 | PKA inhibitor |
| 8387 | -79.5 | cp | BRD-A39230911 | chlorphensin | Muscle relaxant |
| 8373 | -77.93 | cp | BRD-K10852020 | tolcapone | Catechol O methyltransferase inhibitor |
| 8360 | -76.54 | cp | BRD-K52930707 | rescinnamine | ACE inhibitor |
| 8359 | -76.47 | cp | BRD-A74907996 | equol | Estrogen receptor agonist |
| 8358 | -76.42 | cp | BRD-K68174511 | torin-2 | MTOR inhibitor |
| 8357 | -76.32 | cp | BRD-A50157456 | terbutaline | Adrenergic receptor agonist |
| 8356 | -75.84 | cp | BRD-K72034655 | peucedanin | Apoptosis stimulant |
| 8352 | -75.46 | cp | BRD-A09161221 | nomilin | HSP inhibitor |
| 8339 | -74.7 | cp | BRD-K20285085 | fostamatinib | SYK inhibitor |
| 8330 | -74.35 | cp | BRD-K56001384 | antimycin-a | ATP synthase inhibitor |
| 8328 | -74.33 | cp | BRD-A54596827 | solifenacin | Acetylcholine receptor antagonist |
| 8327 | -74.27 | cp | BRD-A04706586 | bucladesine | Adenosine receptor agonist |
| 8321 | -73.89 | cp | BRD-K63068307 | ZSTK-474 | PI3K inhibitor |
| 8319 | -73.83 | cp | BRD-U01690642 | acetyl-geranyl-cysteine | Isoprenylated protein methylation inhibitor |
| 8318 | -73.64 | cp | BRD-A07000685 | hydrocortisone | Glucocorticoid receptor agonist |
| 8316 | -73.53 | cp | BRD-A28746609 | paclitaxel | Tubulin inhibitor |
| 8315 | -73.43 | cp | BRD-U33728988 | QL-X-138 | MTOR inhibitor |
| 8311 | -73.06 | cp | BRD-K18742343 | H-8 | PKA inhibitor |
| 8304 | -72.55 | cp | BRD-K69837166 | trap-101 | Nociceptin/orphanin FQ (NOP) receptor antagonist |
| 8300 | -72.32 | cp | BRD-K01493881 | apigenin | Casein kinase inhibitor |
| 8293 | -71.8 | cp | BRD-K46212057 | voriconazole | Cytochrome P450 inhibitor |
| 8282 | -71.06 | cp | BRD-K08589866 | linsitinib | IGF-1 inhibitor |
| 8271 | -70.34 | cp | BRD-K81729199 | AQ-RA741 | Acetylcholine receptor antagonist |
| 8266 | -69.53 | cp | BRD-K18895904 | olanzapine | Dopamine receptor antagonist |
| 8257 | -68.81 | cp | BRD-A67438293 | treprostinil | Prostacyclin analog |
| 8244 | -67.49 | cp | BRD-A78391468 | prednisolone | Glucocorticoid receptor agonist |
| 8239 | -67.38 | cp | BRD-K43880410 | pregnenolone | glutamate receptor modulator |
| 8238 | -67.37 | cp | BRD-K85925969 | zalcitabine | Nucleoside reverse transcriptase inhibitor |
| 8228 | -66.59 | cp | BRD-K99818283 | PIK-90 | PI3K inhibitor |
| 8221 | -66.32 | cp | BRD-K67578145 | GDC-0879 | RAF inhibitor |
| 8216 | -66.15 | cp | BRD-K82147103 | lofepramine | Norepinephrine reuptake inhibitor |
| 8209 | -65.77 | cp | BRD-K06792661 | narciclasine | Coflilin signaling pathway activator |
| 8207 | -65.75 | cp | BRD-M41783010 | acamprosate | Glutamate receptor antagonist |
| 8206 | -65.69 | cp | BRD-K53523901 | arctigenin | MEK inhibitor |
| 8205 | -65.64 | cp | BRD-K09186807 | KIN001-244 | Phosphoinositide dependent kinase inhibitor |
| 8197 | -65.38 | cp | BRD-K60923938 | veratridine | Sodium channel activator |
| 8179 | -64.2 | cp | BRD-A57457122 | VU-0400071-3 | Glutamate receptor modulator |
| 8178 | -64.14 | cp | BRD-K14807180 | SB-221284 | Serotonin receptor antagonist |
| 8176 | -63.81 | cp | BRD-A14985772 | ascorbyl-palmitate | antioxidant |
| 8155 | -62.25 | cp | BRD-A85280935 | quinpirole | Dopamine receptor agonist |
| 8146 | -61.42 | cp | BRD-K89708791 | rifaximin | RNA synthesis inhibitor |
| 8143 | -61.2 | cp | BRD-K16233984 | eriochrome-black-t | Azo dye |
| 8140 | -61.15 | cp | BRD-K87142802 | veliparib | PARP inhibitor |
| 8139 | -61.06 | cp | BRD-A44448661 | pentobarbital | Barbiturate antiepileptic |
| 8136 | -60.76 | cp | BRD-K24994810 | androstenol | GABA receptor modulator |
| 8135 | -60.71 | cp | BRD-K49404994 | levetiracetam | Calcium channel blocker |
| 8125 | -60.01 | cp | BRD-K40175214 | torin-1 | MTOR inhibitor |
| 8123 | -59.94 | cp | BRD-K73824630 | skatole | Thrombin inhibitor |
| 8116 | -59.71 | cp | BRD-A62057054 | MDL-11939 | Serotonin receptor antagonist |
| 8115 | -59.56 | cp | BRD-K48168960 | propylthiouracil | Thyroid peroxidase inhibitor |
| 8114 | -59.55 | cp | BRD-K13211965 | L-741742 | Dopamine receptor antagonist |
| 8111 | -59.49 | cp | BRD-K01648091 | LE-300 | Dopamine receptor antagonist |
| 8112 | -59.49 | cp | BRD-K21936341 | oxotremorine | Acetylcholine receptor agonist |
| 8109 | -59.43 | cp | BRD-K90885812 | propantheline | Acetylcholine receptor antagonist |
| 8103 | -59.17 | cp | BRD-A26845397 | isamoltan | Adrenergic receptor antagonist |
| 8101 | -58.95 | cp | BRD-K80639402 | SB-258585 | Serotonin receptor antagonist |
| 8098 | -58.75 | cp | BRD-A43082555 | loxoprofen | Cyclooxygenase inhibitor |
| 8097 | -58.72 | cp | BRD-A67981824 | cefotaxime | Bacterial cell wall synthesis inhibitor |
| 8096 | -58.71 | cp | BRD-K88568253 | iproniazid | Monoamine oxidase inhibitor |
| 8090 | -58.38 | cp | BRD-A65282128 | cefazolin | Bacterial cell wall synthesis inhibitor |
| 8086 | -58.27 | cp | BRD-K67445247 | flurofamide | Urease inhibitor |
| 8083 | -58.1 | cp | BRD-A30655177 | LFM-A13 | BTK inhibitor |
| 8082 | -58.06 | cp | BRD-A38747044 | KU-14R | Imidazoline receptor ligand |
| 8079 | -57.96 | cp | BRD-A87125127 | 3-matida | Glutamate receptor antagonist |
| 8077 | -57.88 | cp | BRD-A96799240 | 4-hydroxyretinoic-acid | Retinoid receptor binder |
| 8073 | -57.8 | cp | BRD-K07736136 | VX-702 | p38 MAPK inhibitor |
| 8072 | -57.57 | cp | BRD-A12016240 | LY-278584 | Serotonin receptor antagonist |
| 8069 | -57.46 | cp | BRD-K55301415 | abiraterone | 17,20 lyase inhibitor |
| 8064 | -57.33 | cp | BRD-K92015269 | GBR-12783 | Dopamine uptake inhibitor |
| 8060 | -56.96 | cp | BRD-K88871508 | lisuride | Dopamine receptor agonist |
| 8055 | -56.68 | cp | BRD-K77998258 | ganglioside | SRC activator |
| 8050 | -56.47 | cp | BRD-K08115555 | tyrphostin-AG-1288 | TNF production inhibitor |
| 8047 | -56.33 | cp | BRD-K77286328 | reversine | Aurora kinase inhibitor |
| 8043 | -55.95 | cp | BRD-K15868788 | SDZ-205-557 | Serotonin receptor antagonist |
| 8041 | -55.8 | cp | BRD-K22009844 | phenprobamate | Muscle relaxant |
| 8035 | -55.52 | cp | BRD-K75641298 | metoclopramide | Dopamine receptor antagonist |
| 8032 | -55.34 | cp | BRD-K18316707 | O-1918 | Cannabinoid receptor antagonist |
| 8029 | -55.01 | cp | BRD-A70449690 | forskolin | Adenylyl cyclase activator |
| 8028 | -54.95 | cp | BRD-A68281735 | REV-5901 | Leukotriene receptor antagonist |
| 8025 | -54.81 | cp | BRD-A53952395 | prilocaine | Local anesthetic |
| 8022 | -54.66 | cp | BRD-K58299615 | RO-90-7501 | Beta amyloid inhibitor |
| 8021 | -54.57 | cp | BRD-K47323024 | methapyrilene | Histamine receptor antagonist |
| 8020 | -54.53 | cp | BRD-K26573499 | DMAB-anabaseine | Adrenergic receptor agonist |
| 8014 | -54.31 | cp | BRD-K68756823 | FR-180204 | -666 |
| 8012 | -54.13 | cp | BRD-K67043667 | altretamine | DNA synthesis inhibitor |
| 8009 | -53.93 | cp | BRD-K51967704 | BIIB021 | HSP inhibitor |
| 8008 | -53.86 | cp | BRD-K40992116 | parachlorophenol | Anti-infective |
| 8007 | -53.84 | cp | BRD-K94294671 | OSI-027 | MTOR inhibitor |
| 8005 | -53.74 | cp | BRD-K48935217 | epothilone | Microtubule inhibitor |
| 8000 | -53.49 | cp | BRD-A74980173 | gatifloxacin | Bacterial DNA gyrase inhibitor |
| 7992 | -53.16 | cp | BRD-K21971034 | OM-137 | Aurora kinase inhibitor |
| 7985 | -52.81 | cp | BRD-K67439147 | SIB-1893 | Glutamate receptor antagonist |
| 7983 | -52.54 | cp | BRD-A49225603 | alimemazine | Histamine receptor agonist |
| 7977 | -52.48 | cp | BRD-K59637651 | NSC-119889 | Protein synthesis inhibitor |
| 7969 | -52.02 | cp | BRD-K92817986 | BJM-CSC-19 | MEK inhibitor |
| 7966 | -51.9 | cp | BRD-K19554809 | MK-212 | Serotonin receptor agonist |
| 7960 | -51.79 | cp | BRD-K81855038 | roxatidine | Histamine receptor antagonist |
| 7951 | -51.3 | cp | BRD-K96527333 | dehydroisoandosterone | GABA receptor modulator |
| 7946 | -50.97 | cp | BRD-A76093993 | cromakalim | Potassium channel activator |
| 7945 | -50.96 | cp | BRD-K14767410 | SC-560 | Cyclooxygenase inhibitor |
| 7938 | -50.84 | cp | BRD-K62965247 | tipifarnib-P2 | farnesyltransferase inhibitor |
| 7931 | -50.63 | cp | BRD-A26711594 | nicardipine | Calcium channel blocker |
| 7928 | -50.46 | cp | BRD-A87848830 | bimatoprost | Prostanoid receptor agonist |
| 7926 | -50.41 | cp | BRD-A39172021 | ampiroxicam | Cyclooxygenase inhibitor |
| 7923 | -50.33 | cp | BRD-K80527266 | triacsin-c | Adrenergic receptor antagonist |
| 7921 | -50.17 | cp | BRD-K80325895 | eicosadienoic-acid | -666 |
| 7914 | -50.03 | cp | BRD-A74904029 | EI-231 | Casein kinase inhibitor |
| 7909 | -49.82 | cp | BRD-K62310379 | fluticasone | Glucocorticoid receptor agonist |
| 7907 | -49.74 | cp | BRD-K88789588 | letrozole | Aromatase inhibitor |
| 7906 | -49.7 | cp | BRD-K14965640 | ibuprofen | Cyclooxygenase inhibitor |
| 7904 | -49.68 | cp | BRD-K69600043 | thiethylperazine | Dopamine receptor antagonist |
| 7905 | -49.68 | cp | BRD-K20920669 | cromoglicic-acid | Immunosuppressant |
| 7897 | -49.28 | cp | BRD-K50140147 | NVP-TAE684 | ALK inhibitor |
| 7895 | -48.98 | cp | BRD-K52751261 | TAK-715 | p38 MAPK inhibitor |
| 7892 | -48.91 | cp | BRD-K82823076 | RO-15-4513 | GABA benzodiazepine site receptor inverse agonist |
| 7885 | -48.41 | cp | BRD-K39391626 | ethylestrenol | Progesterone receptor agonist |
| 7881 | -48.2 | cp | BRD-K94887716 | TFMPP | Serotonin receptor agonist |
| 7871 | -47.81 | cp | BRD-A33084410 | 5'-guanidinonaltrindole | Opioid receptor antagonist |
| 7861 | -47.4 | cp | BRD-A98431941 | ephedrine | Adrenergic receptor agonist |
| 7860 | -47.34 | cp | BRD-K67080878 | milrinone | Phosphodiesterase inhibitor |
| 7855 | -47.25 | cp | BRD-A18043272 | phensuximide | Succinimide antiepileptic |
| 7849 | -47.19 | cp | BRD-A39522003 | OMDM-2 | FAAH inhibitor |
| 7848 | -47.12 | cp | BRD-A00100033 | nifurtimox | DNA inhibitor |
| 7846 | -47.11 | cp | BRD-U68942961 | JW-7-24-1 | LCK Inhibitor |
| 7842 | -46.88 | cp | BRD-A81866333 | CGS-21680 | Adenosine receptor agonist |
| 7840 | -46.81 | cp | BRD-K47659338 | EMD-386088 | Serotonin receptor agonist |
| 7837 | -46.62 | cp | BRD-K86204871 | terconazole | Sterol demethylase inhibitor |
| 7832 | -46.56 | cp | BRD-A80928489 | 1-monopalmitin | P-glycoprotein inhibitor |
| 7830 | -46.31 | cp | BRD-A62890442 | 3-methyl-GABA | GABA aminotransferase activator |
| 7829 | -46.21 | cp | BRD-A99571536 | dubinidine | Anti-epileptic |
| 7823 | -45.92 | cp | BRD-K28912512 | nicotinamide | Protein synthesis stimulant |
| 7814 | -45.42 | cp | BRD-K95992530 | Cyclo-[Arg-Gly-Asp-D-Phe-Val] | integrin antagonist |
| 7808 | -45.18 | cp | BRD-K20714604 | RS-56812 | Serotonin receptor partial agonist |
| 7809 | -45.18 | cp | BRD-K48692744 | NU-1025 | PARP inhibitor |
| 7807 | -45.16 | cp | BRD-K59273480 | propentofylline | Adenosine reuptake inhibitor |
| 7806 | -45.14 | cp | BRD-K52172416 | anastrozole | Aromatase inhibitor |
| 7799 | -45.03 | cp | BRD-A92826379 | lupanine | Sodium channel blocker |
| 7797 | -45 | cp | BRD-K44899736 | RO-16-6941 | Monoamine oxidase inhibitor |
| 7794 | -44.96 | cp | BRD-K74195153 | irsogladine | Phosphodiesterase inhibitor |
| 7796 | -44.96 | cp | BRD-K15108141 | gemcitabine | Ribonucleotide reductase inhibitor |
| 7787 | -44.49 | cp | BRD-K17953061 | staurosporine | PKC inhibitor |
| 7786 | -44.47 | cp | BRD-A15530910 | carpindolol | Adrenergic receptor antagonist |
| 7784 | -44.35 | cp | BRD-K24526313 | levcromakalim | Potassium channel activator |
| 7782 | -44.29 | cp | BRD-K49890030 | gavestinel | Glutamate receptor antagonist |
| 7779 | -44.23 | cp | BRD-K60160658 | tiagabine | GABA uptake inhibitor |
| 7776 | -44.11 | cp | BRD-A31575449 | CGP-20712 | Adrenergic receptor antagonist |
| 7775 | -44.08 | cp | BRD-K37798499 | etoposide | Topoisomerase inhibitor |
| 7774 | -44.07 | cp | BRD-K84085265 | CG-930 | JNK inhibitor |
| 7773 | -44 | cp | BRD-K14643723 | 4-(2-Amino-ethyl)-benzenesulfonamide | carbonic anhydrase inhibitor |
| 7772 | -43.99 | cp | BRD-K00337317 | NU-7441 | DNA dependent protein kinase inhibitor |
| 7769 | -43.98 | cp | BRD-A62021152 | WAY-161503 | Serotonin receptor agonist |
| 7759 | -43.73 | cp | BRD-K87991767 | umbelliferone | Cyclooxygenase inhibitor |
| 7760 | -43.73 | cp | BRD-A18917088 | estradiol | Contraceptive agent |
| 7757 | -43.69 | cp | BRD-K40530731 | hyoscyamine | Acetylcholine receptor antagonist |
| 7751 | -43.2 | cp | BRD-K86465814 | HO-013 | PPAR receptor agonist |
| 7750 | -43.01 | cp | BRD-K28863208 | PNU-282987 | Cholinergic receptor agonist |
| 7733 | -42.4 | cp | BRD-K09668667 | benzo(a)pyrene | Carcinogen |
| 7727 | -42.27 | cp | BRD-K46384212 | o-3M3FBS | phospholipase activator |
| 7726 | -42.26 | cp | BRD-A30590053 | MR-16728 | Acetylcholine release enhancer |
| 7725 | -42.19 | cp | BRD-K32977963 | eugenol | Androgen receptor antagonist |
| 7722 | -42.11 | cp | BRD-A18620900 | estriol | Estrogen receptor agonist |
| 7721 | -42.09 | cp | BRD-K52911425 | GDC-0941 | PI3K inhibitor |
| 7720 | -42.06 | cp | BRD-K98763141 | niflumic-acid | Cyclooxygenase inhibitor |
| 7716 | -41.95 | cp | BRD-A87387433 | cefpodoxime | Bacterial cell wall synthesis inhibitor |
| 7712 | -41.83 | cp | BRD-A97674275 | ranolazine | Sodium channel blocker |
| 7707 | -41.64 | cp | BRD-A96485169 | EBPC | Aldose reductase inhibitor |
| 7701 | -41.48 | cp | BRD-K56558538 | ambroxol | Sodium channel blocker |
| 7698 | -41.4 | cp | BRD-K76274772 | MAPP-D-erythro | Ceramidase inhibitor |
| 7690 | -41.09 | cp | BRD-K68620903 | dydrogesterone | Progesterone receptor agonist |
| 7686 | -40.91 | cp | BRD-K46424862 | hymecromone | Monoamine oxidase inhibitor |
| 7685 | -40.88 | cp | BRD-K77793136 | hydroxyfasudil | Rho associated kinase inhibitor |
| 7683 | -40.79 | cp | BRD-K83144676 | olmesartan | Angiotensin antagonist |
| 7677 | -40.53 | cp | BRD-A59303141 | quinethazone | Thiazide diuretic |
| 7675 | -40.41 | cp | BRD-A31312900 | montelukast | Leukotriene receptor antagonist |
| 7674 | -40.4 | cp | BRD-K08556791 | ethoprop | Acetylcholinesterase inhibitor |
| 7672 | -40.32 | cp | BRD-A81402010 | KU-C103443N | CDC inhibitor |
| 7659 | -39.94 | cp | BRD-K97118047 | 4,5,6,7-tetrabromobenzotriazole | Casein kinase inhibitor |
| 7660 | -39.94 | cp | BRD-K26015241 | ODQ | Guanylyl cyclase inhibitor |
| 7658 | -39.92 | cp | BRD-A51829654 | BRL-15572 | Serotonin receptor antagonist |
| 7653 | -39.84 | cp | BRD-K13725475 | rhodomyrtoxin | Cytotoxic agent |
| 7651 | -39.83 | cp | BRD-A55756846 | H-7 | PKA inhibitor |
| 7652 | -39.83 | cp | BRD-K72222507 | quinapril | ACE inhibitor |
| 7642 | -39.66 | cp | BRD-A83431637 | resmethrin | Cytochrome P450 inhibitor |
| 7641 | -39.65 | cp | BRD-K97752965 | nicorandil | Nitric oxide donor |
| 7638 | -39.57 | cp | BRD-A91699651 | chloroquine | Antimalarial |
| 7630 | -39.24 | cp | BRD-K63641886 | cefuroxime | Bacterial cell wall synthesis inhibitor |
| 7622 | -39.07 | cp | BRD-K64746805 | MBCQ | Phosphodiesterase inhibitor |
| 7614 | -38.9 | cp | BRD-A41112154 | oleanolic-acid | G protein-coupled receptor agonist |
| 7612 | -38.85 | cp | BRD-K90027355 | spironolactone | Mineralocorticoid receptor antagonist |
| 7610 | -38.79 | cp | BRD-K79602928 | metformin | Insulin sensitizer |
| 7609 | -38.78 | cp | BRD-K96670504 | lonidamine | Glucokinase inhibitor |
| 7602 | -38.62 | cp | BRD-K23875128 | RHO-kinase-inhibitor-III[rockout] | Rho associated kinase inhibitor |
| 7600 | -38.59 | cp | BRD-K07996107 | harpagoside | Acetylcholinesterase inhibitor |
| 7589 | -38.35 | cp | BRD-K01436366 | XMD-1150 | Leucine rich repeat kinase inhibitor |
| 7590 | -38.35 | cp | BRD-K17349619 | HLI-373 | MDM inhibitor |
| 7587 | -38.25 | cp | BRD-A75517195 | thiazolopyrimidine | CDC inhibitor |
| 7575 | -37.99 | cp | BRD-A85472596 | L-670596 | Prostanoid receptor antagonist |
| 7572 | -37.85 | cp | BRD-K69032158 | diprotin-a | Dipeptidyl peptidase inhibitor |
| 7566 | -37.76 | cp | BRD-K50422030 | clomethiazole | GABA receptor antagonist |
| 7565 | -37.75 | cp | BRD-K91370081 | anisomycin | DNA synthesis inhibitor |
| 7563 | -37.74 | cp | BRD-K66956375 | oleoylethanolamide | Cannabinoid receptor agonist |
| 7562 | -37.7 | cp | BRD-K14536225 | piceid | Glucosidase inhibitor |
| 7561 | -37.66 | cp | BRD-A65013509 | oxybutynin | Acetylcholine receptor antagonist |
| 7560 | -37.58 | cp | BRD-A02176148 | tubaic-acid | Mitochondrial complex I inhibitor |
| 7551 | -37.34 | cp | BRD-A76279427 | myriocin | Serine palmitoyltransferase inhibitor |
| 7544 | -37.11 | cp | BRD-K31283835 | tofacitinib | JAK inhibitor |
| 7541 | -37.09 | cp | BRD-K13514097 | everolimus | MTOR inhibitor |
| 7539 | -37.03 | cp | BRD-K50387473 | XMD-892 | MAP kinase inhibitor |
| 7537 | -37 | cp | BRD-K93880783 | stavudine | DNA directed DNA polymerase inhibitor |
| 7529 | -36.76 | cp | BRD-K28183345 | proguanil | Dihydrofolate reductase inhibitor |
| 7527 | -36.74 | cp | BRD-K14696368 | 9-methyl-5H-6-thia-4,5-diaza-chrysene-6,6-dioxide | NFkB pathway inhibitor |
| 7519 | -36.62 | cp | BRD-K33396764 | alpha-linolenic-acid | Omega 3 fatty acid stimulant |
| 7514 | -36.41 | cp | BRD-K87696786 | LY-456236 | Glutamate receptor antagonist |
| 7513 | -36.33 | cp | BRD-K03319035 | maprotiline | Norepinephrine reuptake inhibitor |
| 7511 | -36.32 | cp | BRD-K18250272 | propoxycaine | Local anesthetic |
| 7508 | -36.19 | cp | BRD-A64933752 | CV-1808 | Adenosine receptor agonist |
| 7504 | -36.13 | cp | BRD-K51751936 | alfadolone | GABA receptor agonist |
| 7503 | -36.12 | cp | BRD-A74500471 | ethambutol | Bacterial cell wall synthesis inhibitor |
| 7497 | -36.01 | cp | BRD-K77008974 | WYE-354 | MTOR inhibitor |
| 7495 | -35.95 | cp | BRD-K62959606 | sphingosine | Ceramidase inhibitor |
| 7494 | -35.92 | cp | BRD-K97509413 | coumestrol | Estrogen receptor agonist |
| 7489 | -35.81 | cp | BRD-A47513740 | calyculin | Protein phosphatase inhibitor |
| 7488 | -35.79 | cp | BRD-A65145453 | ATPA | Glutamate receptor agonist |
| 7487 | -35.74 | cp | BRD-K64890080 | BI-2536 | PLK inhibitor |
| 7486 | -35.72 | cp | BRD-K20152659 | gamma-homolinolenic-acid | Cholesterol inhibitor |
| 7485 | -35.7 | cp | BRD-A54487287 | cortisone | Glucocorticoid receptor agonist |
| 7484 | -35.69 | cp | BRD-K24240364 | GYKI-52466 | Glutamate receptor antagonist |
| 7482 | -35.63 | cp | BRD-K26801045 | pipamperone | Dopamine receptor antagonist |
| 7479 | -35.55 | cp | BRD-K50135270 | GBR-12935 | Dopamine uptake inhibitor |
| 7478 | -35.52 | cp | BRD-K47539947 | tetradecylthioacetic-acid | Lipid peroxidase inhibitor |
| 7475 | -35.5 | cp | BRD-K03600606 | catechin | Beta secretase inhibitor |
| 7472 | -35.31 | cp | BRD-U66370498 | androstanol | CAR antagonist |
| 7462 | -34.76 | cp | BRD-K87919739 | tyrphostin-AG-825 | Receptor tyrosine protein kinase inhibitor |
| 7461 | -34.75 | cp | BRD-A51382177 | fosinopril | ACE inhibitor |
| 7459 | -34.71 | cp | BRD-K11634954 | GBR-13069 | Dopamine uptake inhibitor |
| 7458 | -34.68 | cp | BRD-K79124250 | ioxaglic-acid | Radiopaque medium |
| 7449 | -34.48 | cp | BRD-K51941867 | LM-1685 | Cyclooxygenase inhibitor |
| 7447 | -34.44 | cp | BRD-K32536677 | AGK-2 | SIRT inhibitor |
| 7442 | -34.27 | cp | BRD-K92726801 | hydrastinine | Haemostatic agent |
| 7441 | -34.25 | cp | BRD-K91733562 | secoisolariciresinol | Antioxidant |
| 7435 | -33.98 | cp | BRD-K49865102 | PD-0325901 | MEK inhibitor |
| 7434 | -33.94 | cp | BRD-K08132273 | tyrphostin | EGFR inhibitor |
| 7431 | -33.83 | cp | BRD-A15034104 | bergenin | Interleukin inhibitor |
| 7423 | -33.46 | cp | BRD-K88677950 | PD-198306 | MAP kinase inhibitor |
| 7418 | -33.28 | cp | BRD-K41859756 | NVP-AUY922 | HSP inhibitor |
| 7413 | -33.15 | cp | BRD-K54665485 | R-59022 | Diacylglycerol kinase inhibitor |
| 7412 | -33.14 | cp | BRD-K02953697 | naringin | Cytochrome P450 inhibitor |
| 7407 | -32.95 | cp | BRD-K96354014 | nifedipine | Calcium channel blocker |
| 7405 | -32.93 | cp | BRD-K61177364 | NBI-27914 | CRF receptor antagonist |
| 7396 | -32.74 | cp | BRD-K10466330 | AVA | Nucleophosmin inhibitor |
| 7394 | -32.7 | cp | BRD-K28667793 | pyrazinamide | Fatty acid synthase inhibitor |
| 7393 | -32.69 | cp | BRD-A75479906 | rimantadine | Antiviral |
| 7386 | -32.52 | cp | BRD-K60623809 | SU-11652 | Tyrosine kinase inhibitor |
| 7381 | -32.42 | cp | BRD-K38903228 | hesperidin | Flavanone glycoside |
| 7378 | -32.37 | cp | BRD-K01663662 | diphenidol | Acetylcholine receptor agonist |
| 7379 | -32.37 | cp | BRD-K88560311 | rucaparib | PARP inhibitor |
| 7375 | -32.28 | cp | BRD-K89152108 | liothyronine | Thyroid hormone stimulant |
| 7374 | -32.27 | cp | BRD-K68103045 | CGS-20625 | Benzodiazepine receptor agonist |
| 7366 | -31.99 | cp | BRD-K80672993 | M2-PK-activator | -666 |
| 7358 | -31.63 | cp | BRD-A82096673 | KU-C103428N | CDC inhibitor |
| 7352 | -31.57 | cp | BRD-K05350981 | oligomycin-c | ATPase inhibitor |
| 7353 | -31.57 | cp | BRD-K09537769 | NU-7026 | DNA dependent protein kinase inhibitor |
| 7351 | -31.54 | cp | BRD-K50128260 | sildenafil | Phosphodiesterase inhibitor |
| 7350 | -31.53 | cp | BRD-K54416256 | methimazole | Antithyroid |
| 7349 | -31.5 | cp | BRD-K44353683 | nateglinide | Insulin secretagogue |
| 7335 | -31.06 | cp | BRD-K63533170 | AM-630 | Cannabinoid receptor antagonist |
| 7333 | -31.04 | cp | BRD-K48115423 | 2-(4-methoxybenzylthio)-6-methylpyrimidin-4-ol | Matrix metalloprotease inhibitor |
| 7329 | -30.99 | cp | BRD-K38323065 | phenacetin | Cyclooxygenase inhibitor |
| 7328 | -30.94 | cp | BRD-K84266862 | BRL-50481 | Phosphodiesterase inhibitor |
| 7327 | -30.92 | cp | BRD-A07395371 | esmolol | Adrenergic receptor antagonist |
| 7326 | -30.85 | cp | BRD-K22878149 | SB-205607 | Delta 1 opioid receptor agonist |
| 7324 | -30.78 | cp | BRD-K06750613 | GSK-1059615 | PI3K inhibitor |
| 7319 | -30.66 | cp | BRD-K02113016 | olaparib | PARP inhibitor |
| 7316 | -30.56 | cp | BRD-A38898897 | GW-311616 | Leukocyte elastase inhibitor |
| 7314 | -30.49 | cp | BRD-K48427617 | U-0124 | MEK inhibitor |
| 7312 | -30.48 | cp | BRD-A80213327 | NSC-23766 | Ras GTPase inhibitor |
| 7309 | -30.44 | cp | BRD-K50388907 | fenofibrate | PPAR receptor agonist |
| 7306 | -30.38 | cp | BRD-K94176593 | TWS-119 | Glycogen synthase kinase inhibitor |
| 7301 | -30.27 | cp | BRD-K56596464 | QX-314 | Sodium channel blocker |
| 7300 | -30.26 | cp | BRD-K81128206 | edrophonium | Acetylcholinesterase inhibitor |
| 7298 | -30.14 | cp | BRD-K23204545 | busulfan | DNA inhibitor |
| 7297 | -30.12 | cp | BRD-K97354755 | SU-6656 | SRC inhibitor |
| 7296 | -30.07 | cp | BRD-K67506692 | tyrphostin-AG-126 | ERK1 and ERK2 phosphorylation inhibitor |
| 7292 | -30.02 | cp | BRD-A19053259 | pseudopelletierine | Anthelmintic |
| 7286 | -29.91 | cp | BRD-K06147391 | telenzepine | Acetylcholine receptor antagonist |
| 7285 | -29.86 | cp | BRD-A15435692 | BMY-14802 | Sigma receptor antagonist |
| 7283 | -29.82 | cp | BRD-A66563878 | medetomidine | Adrenergic receptor agonist |
| 7281 | -29.59 | cp | BRD-A67748489 | K3644 | Kinesin-like spindle protein inhibitor |
| 7278 | -29.52 | cp | BRD-A35338386 | NECA | Adenosine receptor agonist |
| 7276 | -29.5 | cp | BRD-K35377380 | I-OMe-AG-538 | IGF-1 inhibitor |
| 7275 | -29.49 | cp | BRD-A29731977 | 17-hydroxyprogesterone-caproate | progesterone receptor agonist |
| 7274 | -29.45 | cp | BRD-K50660797 | epicatechin | Bacterial DNA gyrase inhibitor |
| 7270 | -29.27 | cp | BRD-K98426715 | tubacin | HDAC inhibitor |
| 7265 | -29.18 | cp | BRD-K15891719 | tenofovir | Reverse transcriptase inhibitor |
| 7262 | -29.12 | cp | BRD-K52735702 | cefdinir | Bacterial cell wall synthesis inhibitor |
| 7256 | -29.03 | cp | BRD-K77677632 | SB-200646 | Serotonin receptor antagonist |
| 7253 | -29 | cp | BRD-K12906962 | dichlorobenzamil | Sodium/calcium exchange inhibitor |
| 7252 | -28.94 | cp | BRD-A16934955 | nalbuphine | Opioid receptor agonist |
| 7247 | -28.86 | cp | BRD-K93331255 | lypressin | Vasopressin receptor agonist |
| 7245 | -28.8 | cp | BRD-K63874012 | thioperamide | Histamine receptor antagonist |
| 7243 | -28.74 | cp | BRD-K68488863 | ENMD-2076 | FLT3 inhibitor |
| 7241 | -28.68 | cp | BRD-K31699485 | DMEOB | glutamate receptor modulator |
| 7238 | -28.56 | cp | BRD-K23913458 | coumarin | Vitamin K antagonist |
| 7237 | -28.53 | cp | BRD-A02990301 | lofexidine | Adrenergic receptor agonist |
| 7231 | -28.41 | cp | BRD-K05737787 | isoeugenol | Nitric oxide production inhibitor |
| 7226 | -28.27 | cp | BRD-K63913457 | eicosatrienoic-acid | Vasodilator |
| 7216 | -28.1 | cp | BRD-K94379058 | BML-190 | Cannabinoid receptor inverse agonist |
| 7217 | -28.1 | cp | BRD-K81376179 | TCS-359 | FLT3 inhibitor |
| 7218 | -28.1 | cp | BRD-A23290232 | westcort | Glucocorticoid receptor agonist |
| 7210 | -27.87 | cp | BRD-K73397362 | purmorphamine | Smoothened receptor agonist |
| 7209 | -27.82 | cp | BRD-A69512159 | carbidopa | Aromatic L-amino acid decarboxylase inhibitor |
| 7207 | -27.8 | cp | BRD-K64881305 | ispinesib | Kinesin-like spindle protein inhibitor |
| 7203 | -27.71 | cp | BRD-K36324071 | NF-449 | Purinergic receptor antagonist |
| 7204 | -27.71 | cp | BRD-A26032986 | zaldaride | Calmodulin antagonist |
| 7202 | -27.69 | cp | BRD-K13356952 | methazolamide | Carbonic anhydrase inhibitor |
| 7199 | -27.62 | cp | BRD-K40990712 | hexamethyleneamiloride | Sodium/hydrogen antiport inhibitor |
| 7200 | -27.62 | cp | BRD-K67100011 | pivmecillinam | Bacterial cell wall synthesis inhibitor |
| 7196 | -27.58 | cp | BRD-A46179541 | doxapram | Potassium channel blocker |
| 7189 | -27.35 | cp | BRD-A11135865 | nor-binaltorphimine | Opioid receptor antagonist |
| 7184 | -27.1 | cp | BRD-A23683907 | NAS-181 | Serotonin receptor antagonist |
| 7182 | -27.08 | cp | BRD-K08640512 | RS-100329 | Adrenergic receptor antagonist |
| 7176 | -26.86 | cp | BRD-K45330754 | diethylstilbestrol | Estrogen receptor agonist |
| 7175 | -26.84 | cp | BRD-K53959060 | indirubin | CDK inhibitor |
| 7172 | -26.72 | cp | BRD-K71823332 | epothilone-a | Microtubule stabilizing agent |
| 7166 | -26.6 | cp | BRD-A47598013 | citalopram | Serotonin reuptake inhibitor |
| 7163 | -26.45 | cp | BRD-K52512893 | SC-19220 | Prostanoid receptor antagonist |
| 7156 | -26.3 | cp | BRD-K87990216 | piretanide | Glucocorticoid receptor agonist |
| 7154 | -26.28 | cp | BRD-K74212935 | ergocryptine | Dopamine agonist |
| 7150 | -26.15 | cp | BRD-K22227508 | targinine | Nitric oxide synthase inhibitor |
| 7151 | -26.15 | cp | BRD-K34014345 | naproxol | Anti-inflammatory |
| 7147 | -26.07 | cp | BRD-A94669766 | naringenin | Aromatase inhibitor |
| 7142 | -25.92 | cp | BRD-K41996876 | tyrphostin-1 | EGFR inhibitor |
| 7140 | -25.88 | cp | BRD-K90976994 | dehydrocholic-acid | Bile acid |
| 7138 | -25.85 | cp | BRD-K36529613 | PU-H71 | HSP inhibitor |
| 7133 | -25.75 | cp | BRD-K33864865 | LY-225910 | CCK receptor antagonist |
| 7123 | -25.54 | cp | BRD-K11158509 | tyrphostin-B44 | EGFR inhibitor |
| 7121 | -25.52 | cp | BRD-A17819071 | gedunin | HSP inhibitor |
| 7113 | -25.38 | cp | BRD-K51066026 | aminoindazole | Ionophore |
| 7109 | -25.31 | cp | BRD-K50324045 | avrainvillamide-analog-6 | nucleophosmin inhibitor |
| 7104 | -25.18 | cp | BRD-A88254928 | salbutamol | Adrenergic receptor agonist |
| 7099 | -25.05 | cp | BRD-U97083655 | teicoplanin | Bacterial cell wall synthesis inhibitor |
| 7098 | -25.02 | cp | BRD-A36630025 | SN-38 | Topoisomerase inhibitor |
| 7094 | -24.89 | cp | BRD-A84702196 | penicillin | Bacterial cell wall synthesis inhibitor |
| 7090 | -24.82 | cp | BRD-K92446736 | zatebradine | HCN channel blocker |
| 7085 | -24.73 | cp | BRD-K40887525 | ritanserin | Serotonin receptor antagonist |
| 7080 | -24.58 | cp | BRD-K87932577 | CDK1-5-inhibitor | CDK inhibitor |
| 7079 | -24.57 | cp | BRD-K54316499 | tolterodine | Acetylcholine receptor antagonist |
| 7078 | -24.53 | cp | BRD-A41301928 | bongkrek-acid | Mitochondrial ADP, ATP translocase inhibitor |
| 7075 | -24.47 | cp | BRD-A03623303 | metoprolol | Adrenergic receptor antagonist |
| 7067 | -24.38 | cp | BRD-U82589721 | HG-5-113-01 | Protein kinase inhibitor |
| 7068 | -24.38 | cp | BRD-K27351809 | nomegestrol | Progesterone receptor agonist |
| 7066 | -24.36 | cp | BRD-K72024482 | MRS-1754 | Adenosine receptor antagonist |
| 7063 | -24.26 | cp | BRD-K60640630 | mometasone | Glucocorticoid receptor agonist |
| 7062 | -24.25 | cp | BRD-A65076780 | dihydroergocristine | Adrenergic receptor antagonist |
| 7060 | -24.24 | cp | BRD-A85025557 | NCS-382 | GABA receptor antagonist |
| 7061 | -24.24 | cp | BRD-A32836748 | leu-enkephalin | Opioid receptor agonist |
| 7046 | -23.95 | cp | BRD-K37130656 | rivaroxaban | Coagulation inhibitor |
| 7045 | -23.94 | cp | BRD-K11540476 | EMF-BCA1-64 | Caspase inhibitor |
| 7044 | -23.92 | cp | BRD-A68631409 | evodiamine | ATPase inhibitor |
| 7040 | -23.79 | cp | BRD-A81795050 | U-18666A | Oxidosqualene cyclase inhibitor |
| 7041 | -23.79 | cp | BRD-A66199457 | asiaticoside | Antineoplastic |
| 7038 | -23.78 | cp | BRD-K97863768 | prothionamide | Mycobacterium tuberculosis enoyl-[acyl-carrier-protein] reductase [NADH] (inhA) inhibitor |
| 7037 | -23.77 | cp | BRD-A14798026 | mestranol | Estrogen receptor agonist |
| 7033 | -23.67 | cp | BRD-A32349859 | methyl-angolensate | Apoptosis inhibitor |
| 7032 | -23.63 | cp | BRD-K37792168 | denbufylline | Phosphodiesterase inhibitor |
| 7029 | -23.62 | cp | BRD-K50384076 | 7,4'-dihydroxyflavone | Opioid receptor antagonist |
| 7018 | -23.51 | cp | BRD-K27184429 | levocetirizine | Histamine receptor antagonist |
| 7019 | -23.51 | cp | BRD-A07780951 | orciprenaline | Adrenergic receptor agonist |
| 7017 | -23.5 | cp | BRD-A36074203 | remacemide | Glutamate receptor antagonist |
| 7013 | -23.43 | cp | BRD-K69556541 | ryanodine | Calcium channel blocker |
| 7012 | -23.39 | cp | BRD-K64451768 | GANT-58 | GLI antagonist |
| 7009 | -23.34 | cp | BRD-K17306061 | aprepitant | Tachykinin antagonist |
| 7006 | -23.18 | cp | BRD-K35483542 | alitretinoin | Retinoid receptor agonist |
| 7007 | -23.18 | cp | BRD-A43671941 | oxprenolol | Adrenergic receptor antagonist |
| 7003 | -23.08 | cp | BRD-K17378184 | prestwick-559 | Dopamine receptor agonist |
| 7002 | -23 | cp | BRD-A97104540 | fenoterol | Adrenergic receptor agonist |
| 6999 | -22.89 | cp | BRD-K21283037 | riluzole | Glutamate inhibitor |
| 6996 | -22.85 | cp | BRD-K43736954 | cortisone | Glucocorticoid receptor agonist |
| 6995 | -22.81 | cp | BRD-A84389633 | tropanyl-3,5-dimethylbenzoate | Serotonin receptor antagonist |
| 6990 | -22.68 | cp | BRD-A23359898 | sibutramine | Serotonin reuptake inhibitor |
| 6989 | -22.64 | cp | BRD-K82164249 | andarine | Androgen receptor modulator |
| 6981 | -22.39 | cp | BRD-A25143711 | hydrocortisone | Glucocorticoid receptor agonist |
| 6977 | -22.3 | cp | BRD-K51476772 | ST-638 | Tyrosine kinase inhibitor |
| 6973 | -22.24 | cp | BRD-K92731339 | perindopril | ACE inhibitor |
| 6969 | -22.12 | cp | BRD-K26373640 | IKK-2-inhibitor | IKK inhibitor |
| 6965 | -22.11 | cp | BRD-K20287671 | SU-4312 | PDGFR receptor inhibitor |
| 6964 | -22.09 | cp | BRD-K86958018 | olvanil | TRPV agonist |
| 6963 | -22.06 | cp | BRD-K83010055 | VU-0415374-1 | Glutamate receptor modulator |
| 6961 | -21.99 | cp | BRD-K68997413 | PF-3845 | FAAH inhibitor |
| 6958 | -21.86 | cp | BRD-K66412701 | pazufloxacin | Topoisomerase inhibitor |
| 6959 | -21.86 | cp | BRD-A24122750 | saclofen | GABA receptor antagonist |
| 6954 | -21.82 | cp | BRD-A77291778 | cyclopentolate | Acetylcholine receptor antagonist |
| 6947 | -21.77 | cp | BRD-K96319534 | phentermine | Dopamine uptake inhibitor |
| 6949 | -21.77 | cp | BRD-A13946108 | sulindac | Cyclooxygenase inhibitor |
| 6940 | -21.66 | cp | BRD-K83597974 | pargyline | Monoamine oxidase inhibitor |
| 6935 | -21.59 | cp | BRD-M16762496 | PIK-75 | DNA protein kinase inhibitor |
| 6932 | -21.55 | cp | BRD-K82795137 | loratadine | Histamine receptor antagonist |
| 6929 | -21.51 | cp | BRD-K89210380 | biotin | Vitamin B |
| 6928 | -21.48 | cp | BRD-K08973992 | linoleic-acid | Oxidative stress inducer |
| 6925 | -21.41 | cp | BRD-A20239487 | atenolol | Adrenergic receptor antagonist |
| 6924 | -21.36 | cp | BRD-K56064827 | EI-273 | PKC inhibitor |
| 6917 | -21.18 | cp | BRD-K69650333 | idarubicin | Topoisomerase inhibitor |
| 6916 | -21.17 | cp | BRD-K37618799 | MRS-1220 | Adenosine receptor antagonist |
| 6913 | -21.14 | cp | BRD-M45964048 | verteporfin | Photosensitizing agent |
| 6914 | -21.14 | cp | BRD-K92760278 | riboflavin | Vitamin B |
| 6908 | -21.07 | cp | BRD-K01192156 | tyrphostin-AG-112 | Protein tyrosine kinase inhibitor |
| 6907 | -21.05 | cp | BRD-K31987754 | oleylethanolamide | Cannabinoid receptor agonist |
| 6905 | -21.03 | cp | BRD-K57304726 | PRE-084 | Sigma receptor agonist |
| 6906 | -21.03 | cp | BRD-K35531059 | molsidomine | Guanylyl cyclase activator |
| 6900 | -20.96 | cp | BRD-A85234536 | N6-cyclopentyladenosine | Adenosine receptor agonist |
| 6895 | -20.83 | cp | BRD-K45401373 | betulinic-acid | Apoptosis stimulant |
| 6894 | -20.81 | cp | BRD-A54845972 | dihydroergotamine | Serotonin receptor agonist |
| 6890 | -20.75 | cp | BRD-K70241288 | L-692585 | Growth hormone releasing peptide ligand agonist |
| 6891 | -20.75 | cp | BRD-K12184916 | dactolisib | MTOR inhibitor |
| 6887 | -20.67 | cp | BRD-A65597028 | RX-821002 | Adrenergic receptor antagonist |
| 6881 | -20.61 | cp | BRD-K64614248 | salicin | Anti-inflammatory |
| 6879 | -20.59 | cp | BRD-K40782193 | QX-222 | Sodium channel blocker |
| 6873 | -20.43 | cp | BRD-K10860596 | granisetron | Serotonin receptor antagonist |
| 6868 | -20.28 | cp | BRD-K46142322 | RS-67333 | Serotonin receptor partial agonist |
| 6863 | -20.16 | cp | BRD-K31491153 | 1-phenylbiguanide | Serotonin receptor agonist |
| 6859 | -20.11 | cp | BRD-K77625572 | etomoxir | Carnitine palmitoyltransferase inhibitor |
| 6857 | -19.98 | cp | BRD-K08273968 | griseofulvin | Tubulin inhibitor |
| 6855 | -19.97 | cp | BRD-A87479750 | tenidap | Cyclooxygenase inhibitor |
| 6854 | -19.96 | cp | BRD-K06159959 | CCMQ | Inhibitor of the binding of homoquinolinic acid to non-NMDA sensitive sites |
| 6852 | -19.91 | cp | BRD-K27141178 | SB-203186 | Serotonin receptor antagonist |
| 6851 | -19.9 | cp | BRD-K90259198 | W-7 | Calmodulin antagonist |
| 6847 | -19.76 | cp | BRD-K05181084 | NGB-2904 | Dopamine receptor antagonist |
| 6841 | -19.64 | cp | BRD-A13650332 | flucloxacillin | Bacterial cell wall synthesis inhibitor |
| 6840 | -19.61 | cp | BRD-K45479396 | BP-554 | Serotonin receptor agonist |
| 6839 | -19.59 | cp | BRD-K57011718 | UK-356618 | Metalloproteinase inhibitor |
| 6835 | -19.5 | cp | BRD-K05804044 | AZ-628 | RAF inhibitor |
| 6834 | -19.47 | cp | BRD-A45499626 | UBP-302 | Glutamate receptor antagonist |
| 6830 | -19.44 | cp | BRD-A75552914 | isoxicam | Cyclooxygenase inhibitor |
| 6828 | -19.41 | cp | BRD-K63265447 | docetaxel | Tubulin inhibitor |
| 6829 | -19.41 | cp | BRD-A35511923 | L-803087 | Somatostatin receptor agonist |
| 6826 | -19.39 | cp | BRD-A68039575 | liquiritigenin | Aromatase inhibitor |
| 6822 | -19.33 | cp | BRD-K99174507 | cardiogenol-c | Cardiomyogenesis inducer |
| 6820 | -19.32 | cp | BRD-A59215453 | lobelanidine | Acetylcholine receptor antagonist |
| 6818 | -19.21 | cp | BRD-K94841585 | emodic-acid | Laxative |
| 6816 | -19.16 | cp | BRD-K41051431 | mecillinam | Bacterial cell wall synthesis inhibitor |
| 6811 | -19.08 | cp | BRD-A72441487 | stiripentol | GABA uptake inhibitor |
| 6808 | -19.07 | cp | BRD-A68888262 | azelastine | Histamine receptor antagonist |
| 6809 | -19.07 | cp | BRD-K48367671 | febuxostat | Xanthine oxidase inhibitor |
| 6806 | -19.04 | cp | BRD-A16754160 | ampicillin | Bacterial cell wall synthesis inhibitor |
| 6802 | -18.97 | cp | BRD-A74771556 | nikkomycin | Chitin inhibitor |
| 6801 | -18.95 | cp | BRD-A78377521 | monastrol | Kinesin-like spindle protein inhibitor |
| 6800 | -18.89 | cp | BRD-K62792802 | LY-83583 | Guanylyl cyclase inhibitor |
| 6797 | -18.82 | cp | BRD-K81839095 | estrone | Estrogen receptor agonist |
| 6792 | -18.74 | cp | BRD-K59633790 | VU-0420363-1 | SARS coronavirus 3C-like protease inhibitor |
| 6789 | -18.72 | cp | BRD-K18678457 | ZD-7288 | HCN channel blocker |
| 6784 | -18.61 | cp | BRD-U37049823 | HG-6-64-01 | RAF inhibitor |
| 6783 | -18.6 | cp | BRD-A13188892 | doxazosin | Adrenergic receptor antagonist |
| 6771 | -18.47 | cp | BRD-K24656285 | farnesol | FXR agonist |
| 6768 | -18.42 | cp | BRD-U44700465 | HG-5-88-01 | Protein kinase inhibitor |
| 6763 | -18.35 | cp | BRD-K04993501 | cefixime | Bacterial cell wall synthesis inhibitor |
| 6764 | -18.35 | cp | BRD-K18905250 | ST-91 | Adrenergic receptor agonist |
| 6762 | -18.28 | cp | BRD-K47207162 | zimelidine | Serotonin reuptake inhibitor |
| 6760 | -18.27 | cp | BRD-A64228451 | terreic-acid | BTK inhibitor |
| 6758 | -18.22 | cp | BRD-K26664453 | cytochalasin-b | Microtubule inhibitor |
| 6755 | -18.16 | cp | BRD-K15842202 | eudesmic-acid | -666 |
| 6751 | -18.06 | cp | BRD-A34751532 | homosalate | HSP inducer |
| 6749 | -18.01 | cp | BRD-K15834839 | lobendazole | Anthelmintic |
| 6743 | -17.87 | cp | BRD-A01145011 | zebularine | DNA methyltransferase inhibitor |
| 6740 | -17.8 | cp | BRD-A33447119 | oxfendazole | Anthelmintic |
| 6741 | -17.8 | cp | BRD-K62056274 | quipazine | Serotonin receptor agonist |
| 6739 | -17.79 | cp | BRD-K35723520 | darinaparsin | Apoptosis stimulant |
| 6735 | -17.76 | cp | BRD-A17411484 | carprofen | Cyclooxygenase inhibitor |
| 6734 | -17.74 | cp | BRD-K13664374 | dichloroacetic-acid | Pyruvate dehydrogenase kinase inhibitor |
| 6733 | -17.72 | cp | BRD-K50495309 | SRC-kinase-inhibitor-I | SRC inhibitor |
| 6731 | -17.63 | cp | BRD-K49328571 | dasatinib | BCR-ABL kinase inhibitor |
| 6727 | -17.54 | cp | BRD-A16700644 | isoxsuprine | Adrenergic receptor agonist |
| 6723 | -17.48 | cp | BRD-K96271548 | coumaric-acid | Antioxidant |
| 6722 | -17.47 | cp | BRD-A48237631 | mitomycin-c | DNA alkylating agent |
| 6719 | -17.42 | cp | BRD-K67352070 | TC-2559 | Acetylcholine receptor agonist |
| 6716 | -17.38 | cp | BRD-K32755366 | reserpic-acid | Norepinephrine transporter inhibitor |
| 6715 | -17.37 | cp | BRD-K46056750 | AZD-7762 | CHK inhibitor |
| 6708 | -17.31 | cp | BRD-K06198550 | isorotenone | Mitochondrial complex I inhibitor |
| 6707 | -17.3 | cp | BRD-K76133116 | benzydamine | Membrane integrity inhibitor |
| 6705 | -17.28 | cp | BRD-A10070317 | propranolol | Adrenergic receptor antagonist |
| 6704 | -17.27 | cp | BRD-A96456596 | FPL-55712 | Leukotriene receptor antagonist |
| 6700 | -17.19 | cp | BRD-K52459643 | prostaglandin-e1 | Prostanoid receptor agonist |
| 6697 | -17.08 | cp | BRD-K06388322 | pramipexole | Dopamine receptor agonist |
| 6695 | -17.07 | cp | BRD-K00206590 | P-1075 | ATP channel activator |
| 6696 | -17.07 | cp | BRD-A19661776 | mianserin | Serotonin receptor antagonist |
| 6694 | -17.06 | cp | BRD-K72903603 | zidovudine | Reverse transcriptase inhibitor |
| 6691 | -17.01 | cp | BRD-A73581086 | ergometrine | Adrenergic receptor agonist |
| 6689 | -16.98 | cp | BRD-A39747742 | estradiol-valerate | Estrogen receptor agonist |
| 6688 | -16.97 | cp | BRD-K53979406 | ALX-5407 | Glycine transporter inhibitor |
| 6685 | -16.9 | cp | BRD-K53878242 | MMPX | Phosphodiesterase inhibitor |
| 6681 | -16.84 | cp | BRD-K12513978 | fenbufen | Cyclooxygenase inhibitor |
| 6679 | -16.83 | cp | BRD-K31633810 | DAU-5884 | Acetylcholine receptor antagonist |
| 6675 | -16.78 | cp | BRD-K60298136 | ITE | Aryl hydrocarbon receptor agonist |
| 6676 | -16.78 | cp | BRD-K83302049 | protopine | Histamine receptor antagonist |
| 6674 | -16.77 | cp | BRD-K55991774 | BAS-09104376 | HIV integrase inhibitor |
| 6669 | -16.7 | cp | BRD-K27450477 | EHNA | Adenosine deaminase inhibitor |
| 6668 | -16.69 | cp | BRD-A52193669 | withaferin-a | IKK inhibitor |
| 6666 | -16.68 | cp | BRD-K06854232 | AM-580 | Retinoid receptor agonist |
| 6664 | -16.65 | cp | BRD-A00993607 | alprenolol | Adrenergic receptor antagonist |
| 6661 | -16.61 | cp | BRD-K64606589 | apicidin | HDAC inhibitor |
| 6659 | -16.59 | cp | BRD-A51393488 | noscapine | Bradykinin receptor antagonist |
| 6656 | -16.55 | cp | BRD-K04877770 | FTase-inhibitor-B581 | Farnesyltransferase inhibitor |
| 6651 | -16.43 | cp | BRD-A80574334 | oxalomalic-acid | Isocitrate dehydrogenase inhibitor |
| 6650 | -16.42 | cp | BRD-K20986251 | lithocholic-acid | FXR antagonist |
| 6649 | -16.39 | cp | BRD-K05906022 | limonin | HIV protease inhibitor |
| 6639 | -16.24 | cp | BRD-A42571354 | cetirizine | Histamine receptor antagonist |
| 6638 | -16.21 | cp | BRD-K14888893 | minoxidil | KATP activator |
| 6634 | -16.16 | cp | BRD-K82381502 | acetylcholine | Acetylcholine receptor agonist |
| 6633 | -16.15 | cp | BRD-K79437791 | acetyl-farnesyl-cysteine | Inhibitor of methylation of endogenous isoprenylated proteins |
| 6632 | -16.14 | cp | BRD-K89046952 | ciclacillin | Bacterial cell wall synthesis inhibitor |
| 6630 | -16.11 | cp | BRD-A94543220 | bifonazole | Sterol demethylase inhibitor |
| 6631 | -16.11 | cp | BRD-A79981887 | midodrine | Adrenergic receptor agonist |
| 6627 | -16.1 | cp | BRD-K84175871 | pseudoephedrine | Adrenergic receptor agonist |
| 6618 | -15.96 | cp | BRD-K11373525 | ZD-7155 | Angiotensin receptor antagonist |
| 6617 | -15.95 | cp | BRD-K37846922 | 3,3'-diindolylmethane | CHK inhibitor |
| 6613 | -15.88 | cp | BRD-K18855837 | varenicline | Acetylcholine receptor agonist |
| 6608 | -15.82 | cp | BRD-K82216340 | medroxyprogesterone | progesterone receptor agonist |
| 6607 | -15.81 | cp | BRD-K59456551 | methotrexate | Dihydrofolate reductase inhibitor |
| 6602 | -15.71 | cp | BRD-A99411506 | esculin | Antioxidant |
| 6601 | -15.66 | cp | BRD-K56115039 | BU-226 | Imidazoline receptor ligand |
| 6598 | -15.63 | cp | BRD-K43468059 | byssochlamic-acid | Mycotoxin |
| 6593 | -15.57 | cp | BRD-A29901043 | KIN001-127 | ITK inhibitor |
| 6590 | -15.56 | cp | BRD-K51677086 | erythromycin | NFkB pathway inhibitor |
| 6587 | -15.5 | cp | BRD-A49370193 | RO-60-0175 | Serotonin receptor agonist |
| 6583 | -15.44 | cp | BRD-K95763993 | trapidil | PDGFR receptor inhibitor |
| 6582 | -15.43 | cp | BRD-A36217750 | sulfinpyrazone | Uricosuric blocker |
| 6580 | -15.42 | cp | BRD-K47631482 | bromhexine | Mucolytic agent |
| 6579 | -15.41 | cp | BRD-K49668410 | clarithromycin | Bacterial 50S ribosomal subunit inhibitor |
| 6576 | -15.4 | cp | BRD-K16551401 | PNU-22394 | Serotonin receptor agonist |
| 6577 | -15.4 | cp | BRD-K28453807 | nitrocaramiphen | Cholinergic receptor antagonist |
| 6578 | -15.4 | cp | BRD-K92778217 | mefenamic-acid | Cyclooxygenase inhibitor |
| 6570 | -15.36 | cp | BRD-K09255212 | clioquinol | Chelating agent |
| 6568 | -15.32 | cp | BRD-A97730597 | hexylcaine | Sodium channel blocker |
| 6567 | -15.31 | cp | BRD-K00656370 | 6-aminochrysene | Transferase inhibitor |
| 6563 | -15.25 | cp | BRD-K70490179 | rimcazole | Sigma receptor antagonist |
| 6562 | -15.22 | cp | BRD-K70511574 | sunitinib | PLK inhibitor |
| 6561 | -15.2 | cp | BRD-K57886322 | fluocinonide | Glucocorticoid receptor agonist |
| 6560 | -15.16 | cp | BRD-K55424922 | anpirtoline | Serotonin receptor agonist |
| 6554 | -15.08 | cp | BRD-K71860425 | CDK2-5-inhibitor | CDK inhibitor |
| 6553 | -15.07 | cp | BRD-K32330832 | VER-155008 | HSP inhibitor |
| 6549 | -15.05 | cp | BRD-K50891186 | GR-103691 | Dopamine receptor antagonist |
| 6546 | -14.97 | cp | BRD-A41941932 | vitexin | Antioxidant |
| 6545 | -14.96 | cp | BRD-K08619574 | thioproperazine | Dopamine receptor antagonist |
| 6535 | -14.81 | cp | BRD-A04668240 | CGP-52432 | GABA receptor antagonist |
| 6536 | -14.81 | cp | BRD-A33711280 | metixene | Acetylcholine receptor antagonist |
| 6534 | -14.79 | cp | BRD-A47494775 | dipivefrine | Adrenergic receptor agonist |
| 6531 | -14.78 | cp | BRD-A22713669 | BVT-948 | Tyrosine phosphatase inhibitor |
| 6526 | -14.65 | cp | BRD-K26160755 | RS-45041-190 | Imidazoline receptor agonist |
| 6522 | -14.54 | cp | BRD-K15601958 | SEW-2871 | Lysophospholipid receptor agonist |
| 6521 | -14.52 | cp | BRD-K78084463 | W-12 | Calmodulin antagonist |
| 6520 | -14.49 | cp | BRD-A72711497 | lasalocid | Bacterial permeability inducer |
| 6519 | -14.44 | cp | BRD-K12762134 | XAV-939 | Tankyrase inhibitor |
| 6513 | -14.41 | cp | BRD-A51410489 | yohimbine | Adrenergic receptor antagonist |
| 6511 | -14.37 | cp | BRD-K47598052 | PP-1 | SRC inhibitor |
| 6512 | -14.37 | cp | BRD-K15933101 | ropinirole | Dopamine receptor agonist |
| 6508 | -14.36 | cp | BRD-K13087974 | 4,5-dianilinophthalimide | EGFR inhibitor |
| 6504 | -14.33 | cp | BRD-A64297288 | amlodipine | Calcium channel blocker |
| 6506 | -14.33 | cp | BRD-A29260609 | acebutolol | Adrenergic receptor antagonist |
| 6503 | -14.32 | cp | BRD-A43930669 | L-368899 | Oxytocin receptor antagonist |
| 6499 | -14.27 | cp | BRD-K09635314 | M-3M3FBS | phospholipase activator |
| 6497 | -14.26 | cp | BRD-K60476892 | YC-1 | Guanylyl cyclase activator |
| 6493 | -14.23 | cp | BRD-K73293050 | WZ-3146 | EGFR inhibitor |
| 6487 | -14.16 | cp | BRD-K72816382 | cinalukast | Leukotriene receptor antagonist |
| 6479 | -13.99 | cp | BRD-U88459701 | atorvastatin | HMGCR inhibitor |
| 6477 | -13.98 | cp | BRD-K45861246 | azaperone | Dopamine receptor antagonist |
| 6475 | -13.94 | cp | BRD-K60184833 | tyrphostin-46 | Tyrosine kinase inhibitor |
| 6473 | -13.91 | cp | BRD-K06926592 | tretinoin | Retinoid receptor agonist |
| 6471 | -13.9 | cp | BRD-A50928468 | norgestrel | Progesterone receptor agonist |
| 6469 | -13.88 | cp | BRD-A02367930 | ethinyl-estradiol | DNA directed DNA polymerase stimulant |
| 6470 | -13.88 | cp | BRD-K45117373 | Y-26763 | Potassium channel activator |
| 6468 | -13.86 | cp | BRD-K07881437 | danusertib | Aurora kinase inhibitor |
| 6464 | -13.81 | cp | BRD-K45435259 | SCH-23390 | Dopamine receptor antagonist |
| 6465 | -13.81 | cp | BRD-K46766488 | S-14506 | Serotonin receptor agonist |
| 6461 | -13.78 | cp | BRD-A22707317 | SB-205384 | GABA receptor modulator |
| 6458 | -13.74 | cp | BRD-K67868012 | PI-103 | MTOR inhibitor |
| 6457 | -13.73 | cp | BRD-A24543851 | nornicotine | Acetylcholine receptor agonist |
| 6456 | -13.72 | cp | BRD-A14395271 | mesoridazine | Dopamine receptor antagonist |
| 6453 | -13.63 | cp | BRD-A98299281 | velnacrine | cholinesterase inhibitor |
| 6448 | -13.57 | cp | BRD-K28029915 | dolasetron | Serotonin receptor antagonist |
| 6449 | -13.57 | cp | BRD-K53123955 | niridazole | Phosphofructokinase inhibitor |
| 6446 | -13.54 | cp | BRD-K28578425 | cilostamide | Phosphodiesterase inhibitor |
| 6445 | -13.45 | cp | BRD-K08109516 | L-701324 | Glutamate receptor antagonist |
| 6444 | -13.43 | cp | BRD-K30197592 | 5-methoxytryptamine | Serotonin receptor agonist |
| 6440 | -13.4 | cp | BRD-A62071884 | siguazodan | Phosphodiesterase inhibitor |
| 6427 | -13.07 | cp | BRD-K02404261 | caffeine | Adenosine receptor antagonist |
| 6421 | -12.97 | cp | BRD-K64157027 | ZD-2079 | Adrenergic receptor agonist |
| 6420 | -12.96 | cp | BRD-M72442222 | vicriviroc | CC chemokine receptor antagonist |
| 6418 | -12.93 | cp | BRD-K30743633 | TCPOBOP | CAR agonist |
| 6415 | -12.92 | cp | BRD-A14574269 | UB-165 | Acetylcholine receptor agonist |
| 6414 | -12.91 | cp | BRD-K17110974 | aristolochic-acid | Phospholipase inhibitor |
| 6412 | -12.86 | cp | BRD-K52397688 | amperozide | Dopamine receptor antagonist |
| 6411 | -12.84 | cp | BRD-K59419204 | AM-281 | Cannabinoid receptor antagonist |
| 6406 | -12.82 | cp | BRD-A47144777 | dihydro-7-desacetyldeoxygedunin | HSP inhibitor |
| 6403 | -12.79 | cp | BRD-K01902415 | pirinixic-acid | PPAR receptor agonist |
| 6402 | -12.78 | cp | BRD-K57179821 | crotamiton | Antipruritic |
| 6398 | -12.68 | cp | BRD-K66296774 | fluvastatin | HMGCR inhibitor |
| 6395 | -12.67 | cp | BRD-A96897502 | U-74389F | Lipid peroxidase inhibitor |
| 6394 | -12.63 | cp | BRD-K32247306 | primidone | GABA receptor antagonist |
| 6393 | -12.59 | cp | BRD-A39415247 | norethisterone | Progesterone receptor agonist |
| 6391 | -12.54 | cp | BRD-K02130563 | panobinostat | HDAC inhibitor |
| 6389 | -12.51 | cp | BRD-K44442813 | pidotimod | Interferon receptor agonist |
| 6379 | -12.35 | cp | BRD-K05673000 | dicloxacillin | Bacterial cell wall synthesis inhibitor |
| 6376 | -12.34 | cp | BRD-A65615053 | zacopride | Serotonin receptor antagonist |
| 6374 | -12.3 | cp | BRD-A55393291 | testosterone | Androgen receptor agonist |
| 6373 | -12.29 | cp | BRD-K72541103 | JAK3-inhibitor-I | JAK inhibitor |
| 6371 | -12.25 | cp | BRD-K02407574 | parbendazole | Tubulin inhibitor |
| 6358 | -12.12 | cp | BRD-K70883034 | nimetazepam | GABA receptor agonist |
| 6353 | -12.1 | cp | BRD-A48261811 | argatroban | Thrombin inhibitor |
| 6354 | -12.1 | cp | BRD-K16277217 | piperacetazine | Dopamine receptor antagonist |
| 6355 | -12.1 | cp | BRD-K10042277 | desmethylclozapine | Acetylcholine receptor agonist |
| 6352 | -12.09 | cp | BRD-A84493640 | atovaquone | Mitochondrial electron transport inhibitor |
| 6351 | -12.06 | cp | BRD-K85871428 | SC-68376 | p38 MAPK inhibitor |
| 6347 | -12.04 | cp | BRD-A61793559 | metolazone | Carbonic anhydrase inhibitor |
| 6350 | -12.04 | cp | BRD-K79145749 | dibenzepin | Norepinephrine reuptake inhibitor |
| 6345 | -12 | cp | BRD-K25394294 | oxaprozin | Cyclooxygenase inhibitor |
| 6344 | -11.99 | cp | BRD-K86595100 | chlordiazepoxide | Benzodiazepine receptor agonist |
| 6339 | -11.92 | cp | BRD-K55420858 | mirin | MRE11A exonuclease inhibitor |
| 6340 | -11.92 | cp | BRD-K34170797 | fexaramine | FXR agonist |
| 6335 | -11.81 | cp | BRD-A55369275 | CGP-54626 | GABA receptor antagonist |
| 6331 | -11.72 | cp | BRD-K34820100 | tebuthiuron | Photosynthesis inhibitor |
| 6325 | -11.66 | cp | BRD-K18618618 | cimetidine | Histamine receptor antagonist |
| 6323 | -11.63 | cp | BRD-K45446451 | JZL-184 | Monoacylglucerol lipase inhibitor |
| 6324 | -11.63 | cp | BRD-K11129031 | gemfibrozil | Lipoprotein lipase activator |
| 6317 | -11.56 | cp | BRD-A51714012 | venlafaxine | Adrenergic inhibitor |
| 6316 | -11.55 | cp | BRD-K82143716 | flucytosine | Antifungal |
| 6314 | -11.54 | cp | BRD-K46469693 | SCH-442416 | Adenosine receptor antagonist |
| 6315 | -11.54 | cp | BRD-K72783841 | tyrphostin-AG-555 | EGFR inhibitor |
| 6313 | -11.53 | cp | BRD-K97158071 | droperidol | Dopamine receptor antagonist |
| 6312 | -11.52 | cp | BRD-K51805276 | temefos | Cholinesterase inhibitor |
| 6308 | -11.43 | cp | BRD-K24132293 | piperlongumine | Glutathione transferase inhibitor |
| 6305 | -11.39 | cp | BRD-K99107520 | felbamate | Glutamate receptor antagonist |
| 6301 | -11.36 | cp | BRD-K49372556 | mofezolac | Cyclooxygenase inhibitor |
| 6294 | -11.3 | cp | BRD-K86887724 | dofetilide | Potassium channel blocker |
| 6295 | -11.3 | cp | BRD-K57546357 | prunetin | Breast cancer resistance protein inhibitor |
| 6290 | -11.26 | cp | BRD-K56509348 | BMS-182874 | Endothelin receptor antagonist |
| 6283 | -11.07 | cp | BRD-A41304429 | practolol | Adrenergic receptor antagonist |
| 6279 | -11.05 | cp | BRD-K31792052 | pifithrin | Interleukin receptor antagonist |
| 6275 | -11.03 | cp | BRD-K89162000 | tandutinib | FLT3 inhibitor |
| 6272 | -11.02 | cp | BRD-K88551539 | CAY-10585 | HIF modulator |
| 6273 | -11.02 | cp | BRD-K04853698 | LDN-193189 | Serine/threonine kinase inhibitor |
| 6274 | -11.02 | cp | BRD-K01095011 | finasteride | 5-alpha reductase inhibitor |
| 6270 | -11.01 | cp | BRD-A41722204 | sulmazole | Adenosine receptor antagonist |
| 6266 | -11 | cp | BRD-K53972329 | ruxolitinib | JAK inhibitor |
| 6261 | -10.92 | cp | BRD-K39339537 | epirizole | Cyclooxygenase inhibitor |
| 6260 | -10.91 | cp | BRD-K92678294 | irilin-a | Isoflavone |
| 6258 | -10.88 | cp | BRD-A09495397 | bicuculline | GABA receptor antagonist |
| 6255 | -10.86 | cp | BRD-K30677119 | PP-30 | RAF inhibitor |
| 6256 | -10.86 | cp | BRD-K76840893 | RS-17053 | Adrenergic receptor antagonist |
| 6254 | -10.85 | cp | BRD-K13810148 | givinostat | HDAC inhibitor |
| 6252 | -10.82 | cp | BRD-K19352500 | prochlorperazine | Dopamine receptor antagonist |
| 6250 | -10.69 | cp | BRD-K53913732 | SB-408124 | Orexin receptor antagonist |
| 6248 | -10.67 | cp | BRD-U94846492 | quinine | Hemozoin biocrystallization inhibitor |
| 6245 | -10.64 | cp | BRD-K04010869 | prostaglandin-a1 | HSP inducer |
| 6243 | -10.63 | cp | BRD-K26521938 | dinoprostone | Prostanoid receptor agonist |
| 6240 | -10.61 | cp | BRD-K68095457 | palmitoylethanolamide | Cannabinoid receptor agonist |
| 6237 | -10.59 | cp | BRD-K85030058 | benactyzine | Acetylcholine receptor antagonist |
| 6239 | -10.59 | cp | BRD-K55044200 | amoxicillin | Penicillin binding protein inhibitor |
| 6232 | -10.56 | cp | BRD-K35240538 | methylprednisolone | Glucocorticoid receptor agonist |
| 6234 | -10.56 | cp | BRD-A45543382 | metrizamide | Radiopaque medium |
| 6231 | -10.54 | cp | BRD-K12002134 | megestrol | progesterone receptor agonist |
| 6221 | -10.45 | cp | BRD-K27305650 | LY-294002 | MTOR inhibitor |
| 6222 | -10.45 | cp | BRD-A90799790 | isradipine | Calcium channel blocker |
| 6218 | -10.43 | cp | BRD-K76568384 | PHTPP | Estrogen receptor antagonist |
| 6213 | -10.4 | cp | BRD-K36965586 | m-chlorophenylbiguanide | Serotonin receptor agonist |
| 6206 | -10.36 | cp | BRD-A90311807 | cilastatin | Dehydropeptidase inhibitor |
| 6207 | -10.36 | cp | BRD-K43290182 | Ro-04-6790 | Serotonin receptor antagonist |
| 6198 | -10.26 | cp | BRD-K67977190 | eprosartan | Angiotensin receptor antagonist |
| 6199 | -10.26 | cp | BRD-K49456190 | prima-1-met | thioredoxin inhibitor |
| 6197 | -10.24 | cp | BRD-K06895174 | cisapride | Serotonin receptor agonist |
| 6193 | -10.19 | cp | BRD-K71103788 | duloxetine | Serotonin reuptake inhibitor |
| 6195 | -10.19 | cp | BRD-K63430059 | methoxsalen | DNA synthesis inhibitor |
| 6191 | -10.09 | cp | BRD-K25906698 | immepip | Histamine receptor agonist |
| 6186 | -10.02 | cp | BRD-A79314293 | cephalosporanic-acid | Bacterial cell wall synthesis inhibitor |
| 6184 | -10.01 | cp | BRD-K54708045 | nTZDpa | PPAR receptor agonist |
| 6185 | -10.01 | cp | BRD-K60174629 | z-prolyl-prolinal | Prolyl endopeptidase inhibitor |
| 6183 | -10 | cp | BRD-K62609077 | scoulerine | Adrenergic receptor antagonist |
| 6181 | -9.96 | cp | BRD-K82484965 | carmoxirole | Dopamine receptor agonist |
| 6172 | -9.89 | cp | BRD-K94080537 | diethyltoluamide | DEET activator of fly antenna ionotropic receptor IR40a |
| 6173 | -9.89 | cp | BRD-A69636825 | diltiazem | Calcium channel blocker |
| 6170 | -9.84 | cp | BRD-A39646320 | HC-toxin | HDAC inhibitor |
| 6166 | -9.83 | cp | BRD-K63516691 | T-0156 | Phosphodiesterase inhibitor |
| 6167 | -9.83 | cp | BRD-K29178788 | dictamnine | Furoquinoline alkaloid |
| 6168 | -9.83 | cp | BRD-A11813248 | AM-92016 | Potassium channel blocker |
| 6165 | -9.77 | cp | BRD-A96882008 | L-732138 | Tachykinin antagonist |
| 6162 | -9.71 | cp | BRD-K41731458 | triclosan | Enoyl-[acyl-carrier-protein] reductase [NADH] inhibitor |
| 6160 | -9.65 | cp | BRD-K40213712 | SAL-1 | Adenosine receptor antagonist |
| 6157 | -9.64 | cp | BRD-K97365803 | PI-828 | PI3K inhibitor |
| 6158 | -9.64 | cp | BRD-K46018455 | bezafibrate | PPAR receptor agonist |
| 6155 | -9.58 | cp | BRD-K66615216 | moxifloxacin | Bacterial DNA gyrase inhibitor |
| 6152 | -9.56 | cp | BRD-A79672927 | tropicamide | Acetylcholine receptor antagonist |
| 6148 | -9.55 | cp | BRD-K28115081 | apafant | Platelet activating factor receptor antagonist |
| 6147 | -9.54 | cp | BRD-K93658967 | aloisine | CDK inhibitor |
| 6144 | -9.51 | cp | BRD-A42553870 | L-152804 | Neuropeptide receptor antagonist |
| 6143 | -9.49 | cp | BRD-A55815733 | phylloquinone | Vitamin K |
| 6141 | -9.48 | cp | BRD-K36740062 | GSK-1070916 | Aurora kinase inhibitor |
| 6138 | -9.45 | cp | BRD-K02715688 | hydrastine | Tyrosine hydroxylase inhibitor |
| 6137 | -9.44 | cp | BRD-A16444946 | acarbose | Glucosidase inhibitor |
| 6134 | -9.42 | cp | BRD-K63945320 | dihydrosamidin | Phospholipase inhibitor |
| 6131 | -9.37 | cp | BRD-K48869804 | icilin | TRPV agonist |
| 6128 | -9.33 | cp | BRD-K93176058 | AC-55649 | Retinoid receptor agonist |
| 6126 | -9.3 | cp | BRD-K59369769 | tozasertib | Aurora kinase inhibitor |
| 6121 | -9.26 | cp | BRD-K80778372 | RO-19-4605 | GABA benzodiazepine site receptor inverse agonist |
| 6118 | -9.24 | cp | BRD-K75532464 | FTI-276 | Farnesyltransferase inhibitor |
| 6117 | -9.23 | cp | BRD-K17140735 | SCH-79797 | Proteasome inhibitor |
| 6111 | -9.13 | cp | BRD-K18779551 | bifemelane | Acetylcholine release stimulant |
| 6110 | -9.11 | cp | BRD-K26429091 | J-104129 | Acetylcholine receptor antagonist |
| 6104 | -9.06 | cp | BRD-K82577285 | dipropyl-dopamine | Dopamine receptor agonist |
| 6100 | -9.02 | cp | BRD-K81521265 | dicyclohexylurea | Epoxide hydolase inhibitor |
| 6099 | -9 | cp | BRD-K73319509 | PF-04217903 | c-Met inhibitor |
| 6098 | -8.98 | cp | BRD-A09925278 | etilefrine | Adrenergic receptor agonist |
| 6094 | -8.92 | cp | BRD-K42748308 | XE-991 | Potassium channel blocker |
| 6092 | -8.9 | cp | BRD-A95096829 | PNU-96415E | Dopamine receptor antagonist |
| 6090 | -8.86 | cp | BRD-K43330982 | JTE-013 | Lysophospholipid receptor antagonist |
| 6089 | -8.85 | cp | BRD-K02526760 | QS-11 | ARFGAP inhibitor |
| 6085 | -8.76 | cp | BRD-K02867583 | minaprine | Serotonin reuptake inhibitor |
| 6083 | -8.75 | cp | BRD-A68589262 | troxipide | Glucosamine synthetase stimulant |
| 6084 | -8.75 | cp | BRD-K72093121 | vidarabine | Antiviral |
| 6080 | -8.67 | cp | BRD-K78373679 | RO-3306 | CDK inhibitor |
| 6078 | -8.66 | cp | BRD-K31542390 | mycophenolic-acid | Dehydrogenase inhibitor |
| 6074 | -8.6 | cp | BRD-A77299732 | salubrinal | Eukaryotic translation initiation factor inhibitor |
| 6072 | -8.58 | cp | BRD-K59597909 | phenothiazine | Dopamine receptor antagonist |
| 6071 | -8.57 | cp | BRD-K73196317 | urapidil | Adrenergic receptor antagonist |
| 6065 | -8.5 | cp | BRD-K49481516 | galantamine | Acetylcholinesterase inhibitor |
| 6066 | -8.5 | cp | BRD-K95655893 | MAZ-51 | VEGFR inhibitor |
| 6058 | -8.45 | cp | BRD-A54490543 | pirlindole | Monoamine oxidase inhibitor |
| 6051 | -8.35 | cp | BRD-A48257147 | PHCCC | Glutamate receptor agonist |
| 6046 | -8.31 | cp | BRD-K42221274 | NNC-711 | GAT inhibitor |
| 6047 | -8.31 | cp | BRD-A55946879 | BW-B70C | Lipoxygenase inhibitor |
| 6045 | -8.27 | cp | BRD-K75615183 | talipexole | Adrenergic receptor agonist |
| 6042 | -8.24 | cp | BRD-A73368467 | fexofenadine | Histamine receptor antagonist |
| 6041 | -8.22 | cp | BRD-K66353228 | zoxazolamine | Myorelaxant |
| 6028 | -8.08 | cp | BRD-K12120659 | GR-144053 | Integrin antagonist |
| 6027 | -8.07 | cp | BRD-A29289453 | PCA-4248 | Platelet activating factor receptor antagonist |
| 6023 | -8.04 | cp | BRD-K58772419 | AZD-6482 | PI3K inhibitor |
| 6020 | -8.03 | cp | BRD-A01317026 | 7,8-dihydro-L-biopterin | Dihydroneopterin aldolase inhibitor |
| 6021 | -8.03 | cp | BRD-K12244279 | MEK1-2-inhibitor | MEK inhibitor |
| 6022 | -8.03 | cp | BRD-K34608650 | BRD-K34608650 | Cannabinoid receptor agonist |
| 6016 | -8.02 | cp | BRD-K00675675 | CL-82198 | Metalloproteinase inhibitor |
| 6013 | -7.99 | cp | BRD-K53220666 | trimetozine | Sedative |
| 6010 | -7.93 | cp | BRD-K42098891 | protriptyline | Tricyclic antidepressant |
| 6007 | -7.89 | cp | BRD-K64341947 | CFM-1571 | Guanylate cyclase activator |
| 6005 | -7.85 | cp | BRD-K80315159 | DPPE | Histamine receptor antagonist |
| 6004 | -7.83 | cp | BRD-A78942461 | ICI-118551 | Adrenergic receptor antagonist |
| 6002 | -7.82 | cp | BRD-K76723084 | isotretinoin | Retinoid receptor agonist |
| 6000 | -7.78 | cp | BRD-K36153907 | KU-C103885 | Cystic fibrosis transmembrane conductance regulator inhibitor |
| 6001 | -7.78 | cp | BRD-K57080016 | selumetinib | MEK inhibitor |
| 5994 | -7.73 | cp | BRD-A55416093 | colforsin | Adenylyl cyclase activator |
| 5995 | -7.73 | cp | BRD-K52313696 | tacedinaline | HDAC inhibitor |
| 5991 | -7.72 | cp | BRD-K75089421 | procainamide | Sodium channel blocker |
| 5992 | -7.72 | cp | BRD-K87226815 | cycloserine | Bacterial cell wall synthesis inhibitor |
| 5988 | -7.69 | cp | BRD-K14550461 | doxercalciferol | Vitamin D receptor agonist |
| 5986 | -7.68 | cp | BRD-K73999723 | telmisartan | Angiotensin receptor antagonist |
| 5983 | -7.64 | cp | BRD-K24576554 | AT-9283 | JAK inhibitor |
| 5984 | -7.64 | cp | BRD-A05352148 | ipratropium | Acetylcholine receptor antagonist |
| 5980 | -7.63 | cp | BRD-K46556543 | canrenoic-acid | Mineralocorticoid receptor antagonist |
| 5981 | -7.63 | cp | BRD-K91290917 | amodiaquine | Histamine receptor agonist |
| 5970 | -7.53 | cp | BRD-K63550407 | erythromycin | NFkB pathway inhibitor |
| 5967 | -7.5 | cp | BRD-K17896185 | FIT | Opioid receptor agonist |
| 5966 | -7.49 | cp | BRD-K95676198 | JAK3-inhibitor-V | JAK inhibitor |
| 5965 | -7.45 | cp | BRD-K93433262 | alfacalcidol | Vitamin D receptor agonist |
| 5963 | -7.43 | cp | BRD-K16554956 | PTB1 | AMPK activator |
| 5959 | -7.4 | cp | BRD-A31521121 | methocarbamol | Muscle relaxant |
| 5958 | -7.39 | cp | BRD-K97056771 | HY-11007 | BCR-ABL kinase inhibitor |
| 5957 | -7.38 | cp | BRD-K54314721 | zolmitriptan | Serotonin receptor agonist |
| 5956 | -7.37 | cp | BRD-A45498368 | WYE-125132 | MTOR inhibitor |
| 5952 | -7.29 | cp | BRD-K20141153 | atomoxetine | Norepinephrine transporter inhibitor |
| 5948 | -7.23 | cp | BRD-K23922020 | arecaidine | Acetylcholine receptor agonist |
| 5947 | -7.22 | cp | BRD-K15567136 | papaverine | Phosphodiesterase inhibitor |
| 5946 | -7.21 | cp | BRD-A39390670 | rabeprazole | ATPase inhibitor |
| 5941 | -7.15 | cp | BRD-A61856038 | tremulacin | Lipoxygenase inhibitor |
| 5942 | -7.15 | cp | BRD-K55034111 | pefloxacin | Bacterial DNA gyrase inhibitor |
| 5939 | -7.13 | cp | BRD-K81209512 | AG-494 | EGFR inhibitor |
| 5938 | -7.12 | cp | BRD-K59574735 | ubenimex | Leukotriene inhibitor |
| 5933 | -7.07 | cp | BRD-K16195444 | oxymetazoline | Adrenergic receptor agonist |
| 5931 | -7.04 | cp | BRD-K25741894 | skimmianine | Acetylcholinesterase inhibitor |
| 5928 | -7.03 | cp | BRD-K29359156 | ebselen | H+/K+-ATPase inhibitor |
| 5927 | -7.02 | cp | BRD-K18574842 | nafcillin | Bacterial cell wall synthesis inhibitor |
| 5922 | -6.94 | cp | BRD-K11630072 | carmofur | Thymidylate synthase inhibitor |
| 5923 | -6.94 | cp | BRD-A95696066 | nisoxetine | Norepinephrine reuptake inhibitor |
| 5920 | -6.93 | cp | BRD-K46678324 | RHO-kinase-inhibitor-II | Rho associated kinase inhibitor |
| 5915 | -6.87 | cp | BRD-K57569181 | pentoxifylline | Phosphodiesterase inhibitor |
| 5911 | -6.84 | cp | BRD-A75402480 | desoxycorticosterone | Mineralocorticoid receptor agonist |
| 5912 | -6.84 | cp | BRD-A84389091 | L-655708 | GABA receptor inverse agonist |
| 5913 | -6.84 | cp | BRD-A44090213 | indoprofen | Cyclooxygenase inhibitor |
| 5905 | -6.8 | cp | BRD-K60219430 | serdemetan | MDM inhibitor |
| 5906 | -6.8 | cp | BRD-A65440446 | cimaterol | Adrenergic receptor agonist |
| 5901 | -6.79 | cp | BRD-K51671335 | sulpiride | Dopamine receptor antagonist |
| 5902 | -6.79 | cp | BRD-K48923948 | BMS-641988 | Androgen receptor antagonist |
| 5904 | -6.79 | cp | BRD-K36927236 | glibenclamide | Sulfonylurea |
| 5895 | -6.73 | cp | BRD-K70358946 | aripiprazole | Serotonin receptor agonist |
| 5896 | -6.73 | cp | BRD-K07117950 | imperatorin | CDK inhibitor |
| 5890 | -6.7 | cp | BRD-K22828899 | TUL-XXI039 | Serine/threonine kinase inhibitor |
| 5888 | -6.69 | cp | BRD-K72264770 | QW-BI-011 | Histone lysine methyltransferase inhibitor |
| 5886 | -6.67 | cp | BRD-K32412559 | morphothebaine | Adrenergic receptor antagonist |
| 5887 | -6.67 | cp | BRD-A56675431 | altizide | Thiazide diuretic |
| 5881 | -6.62 | cp | BRD-K14282469 | LY-165163 | Serotonin receptor antagonist |
| 5883 | -6.62 | cp | BRD-A66435872 | HTMT | Histamine receptor agonist |
| 5875 | -6.55 | cp | BRD-A77050075 | heraclenol | Vitamin K antagonist |
| 5876 | -6.55 | cp | BRD-A74667430 | etodolac | Cyclooxygenase inhibitor |
| 5871 | -6.51 | cp | BRD-K14791739 | fluticasone | Glucocorticoid receptor agonist |
| 5867 | -6.49 | cp | BRD-K60038276 | irbesartan | Angiotensin receptor antagonist |
| 5859 | -6.41 | cp | BRD-K81783531 | VX-222 | HCV inhibitor |
| 5860 | -6.41 | cp | BRD-A80960055 | celastrol | Anti-inflammatory |
| 5853 | -6.36 | cp | BRD-A97739905 | ketoprofen | Cyclooxygenase inhibitor |
| 5850 | -6.31 | cp | BRD-K11801786 | trimidox | Ribonucleotide reductase inhibitor |
| 5847 | -6.29 | cp | BRD-K07212038 | selinidin | Mast cell stabilizer |
| 5848 | -6.29 | cp | BRD-A75478957 | PD-166793 | Metalloproteinase inhibitor |
| 5842 | -6.26 | cp | BRD-A26097136 | bulleyaconitine-a | Non-opiod analgesic |
| 5838 | -6.24 | cp | BRD-K17743697 | KB-R7943 | Sodium/calcium exchange inhibitor |
| 5839 | -6.24 | cp | BRD-K54411430 | robustic-acid | cAMP inhibitor |
| 5837 | -6.19 | cp | BRD-A41519720 | ezetimibe | Niemann-Pick C1-like 1 protein antagonist |
| 5832 | -6.16 | cp | BRD-K14920963 | erythrosine | Coloring agent |
| 5831 | -6.15 | cp | BRD-K56343971 | vemurafenib | RAF inhibitor |
| 5827 | -6.13 | cp | BRD-A29082194 | gitoxigenin | ATPase inhibitor |
| 5820 | -6.06 | cp | BRD-K84639753 | safinamide | Dopamine uptake inhibitor |
| 5816 | -6.04 | cp | BRD-K18799075 | BAY-59-3074 | Cannabinoid receptor partial agonist |
| 5812 | -6.02 | cp | BRD-K25224017 | pirenperone | Serotonin receptor antagonist |
| 5808 | -5.99 | cp | BRD-K93460210 | lamotrigine | Serotonin receptor antagonist |
| 5809 | -5.99 | cp | BRD-A43849199 | karakoline | Phytotoxin |
| 5803 | -5.97 | cp | BRD-A34706053 | CGP-12177 | Adrenergic receptor agonist |
| 5802 | -5.96 | cp | BRD-K24675965 | LY-288513 | CCK receptor antagonist |
| 5799 | -5.95 | cp | BRD-K64785675 | TG100-115 | -666 |
| 5798 | -5.93 | cp | BRD-A99833829 | bethanechol | Acetylcholine receptor agonist |
| 5795 | -5.92 | cp | BRD-K89375097 | pirenzepine | Acetylcholine receptor antagonist |
| 5794 | -5.9 | cp | BRD-K14221570 | benzopurpurin-4b | HIV entry inhibitor |
| 5790 | -5.88 | cp | BRD-A60070924 | alpha-estradiol | Estrogen receptor agonist |
| 5791 | -5.88 | cp | BRD-K14993104 | bemegride | Chemoreceptor agonist |
| 5792 | -5.88 | cp | BRD-A37776212 | ICI-204448 | Opioid receptor agonist |
| 5788 | -5.87 | cp | BRD-K63151507 | MNITMT | Lymphocyte inhibitor |
| 5787 | -5.85 | cp | BRD-A92670106 | tocainide | Sodium channel blocker |
| 5786 | -5.84 | cp | BRD-A50764878 | MDL-73005EF | Serotonin receptor antagonist |
| 5782 | -5.82 | cp | BRD-K98157055 | SIB-1757 | Glutamate receptor antagonist |
| 5783 | -5.82 | cp | BRD-K63165456 | norcyclobenzaprine | Adrenergic receptor agonist |
| 5779 | -5.78 | cp | BRD-A64977602 | mirtazapine | Adrenergic receptor antagonist |
| 5778 | -5.77 | cp | BRD-K54759182 | dosulepin | Norepinephrine reuptake inhibitor |
| 5775 | -5.75 | cp | BRD-K99029477 | prometon | Photosynthesis inhibitor |
| 5773 | -5.74 | cp | BRD-K61341215 | vecuronium | Acetylcholine receptor antagonist |
| 5771 | -5.71 | cp | BRD-K00234327 | RU-24969 | Serotonin receptor agonist |
| 5767 | -5.7 | cp | BRD-K67102207 | phenylbutyrate | HDAC inhibitor |
| 5765 | -5.68 | cp | BRD-A94709349 | metaxalone | Muscle relaxant |
| 5762 | -5.67 | cp | BRD-K14200658 | syrosingopine | Vesicular monoamine transporter inhibitor |
| 5764 | -5.67 | cp | BRD-A91555231 | norepinephrine | Adrenergic receptor agonist |
| 5760 | -5.66 | cp | BRD-A65671304 | candesartan | Angiotensin receptor antagonist |
| 5757 | -5.64 | cp | BRD-A56987319 | SQ-22536 | Adenylyl cyclase inhibitor |
| 5759 | -5.64 | cp | BRD-A29644307 | nomifensine | Dopamine uptake inhibitor |
| 5753 | -5.61 | cp | BRD-K32710582 | EI-247 | IGF-1 inhibitor |
| 5751 | -5.6 | cp | BRD-A71657825 | 2-(biphenyl-4-ylsulfonamido)pentanedioic-acid | Matrix metalloprotease inhibitor |
| 5746 | -5.56 | cp | BRD-K32828673 | chelidonine | Tubulin inhibitor |
| 5742 | -5.53 | cp | BRD-K94144010 | cotinine | Nicotine metabolite |
| 5741 | -5.51 | cp | BRD-A25576662 | streptozotocin | DNA alkylating agent |
| 5732 | -5.46 | cp | BRD-K61480498 | epoxycholesterol | LXR agonist |
| 5730 | -5.45 | cp | BRD-K64514229 | toltrazuril | Antiprotozoal |
| 5727 | -5.44 | cp | BRD-A90515964 | guaifenesin | Expectorant |
| 5729 | -5.44 | cp | BRD-K13571841 | pepstatin | Aspartic protease inhibitor |
| 5724 | -5.43 | cp | BRD-K01612348 | meropenem | Bacterial cell wall synthesis inhibitor |
| 5725 | -5.43 | cp | BRD-K66788707 | fludarabine | DNA synthesis inhibitor |
| 5704 | -5.27 | cp | BRD-K54704028 | BAY-36-7620 | Glutamate receptor antagonist |
| 5700 | -5.25 | cp | BRD-K32830106 | guanfacine | Adrenergic receptor agonist |
| 5696 | -5.22 | cp | BRD-A20131130 | 2',5'-dideoxyadenosine | Adenylyl cyclase inhibitor |
| 5690 | -5.16 | cp | BRD-A39290993 | cyproterone | Androgen receptor antagonist |
| 5688 | -5.13 | cp | BRD-K81062487 | taurocholic-acid | Bile acid |
| 5676 | -5 | cp | BRD-A59985574 | topotecan | Topoisomerase inhibitor |
| 5677 | -5 | cp | BRD-K34154330 | tracazolate | GABA receptor modulator |
| 5673 | -4.97 | cp | BRD-K55703048 | latrepirdine | Glutamate receptor antagonist |
| 5675 | -4.97 | cp | BRD-A50311610 | meclozine | CAR agonist |
| 5667 | -4.9 | cp | BRD-K42095107 | daidzein | Estrogen receptor agonist |
| 5664 | -4.86 | cp | BRD-A54236247 | racephedrine | Adrenergic receptor agonist |
| 5665 | -4.86 | cp | BRD-K40965114 | cyanopindolol | Adrenergic receptor antagonist |
| 5661 | -4.82 | cp | BRD-K34581968 | BMS-536924 | IGF-1 inhibitor |
| 5656 | -4.79 | cp | BRD-K79366068 | PSB-069 | NTPDase inhibitor |
| 5659 | -4.79 | cp | BRD-K91601245 | mercaptopurine | Immunosuppressant |
| 5652 | -4.77 | cp | BRD-K61401890 | deguelin | NADH-ubiquinone oxidoreductase (Complex I) inhibitor |
| 5649 | -4.76 | cp | BRD-A56359832 | zileuton | Leukotriene inhibitor |
| 5650 | -4.76 | cp | BRD-A93206962 | L-755507 | Adrenergic receptor agonist |
| 5639 | -4.72 | cp | BRD-K37848908 | ceforanide | Penicillin binding protein inhibitor |
| 5640 | -4.72 | cp | BRD-A72703248 | SKF-96365 | Calcium channel blocker |
| 5645 | -4.72 | cp | BRD-K47639036 | flavoxate | Acetylcholine receptor antagonist |
| 5646 | -4.72 | cp | BRD-K90543092 | levonorgestrel | Estrogen receptor agonist |
| 5636 | -4.7 | cp | BRD-A84174393 | meloxicam | Cyclooxygenase inhibitor |
| 5632 | -4.69 | cp | BRD-K40624912 | ZM-39923 | JAK inhibitor |
| 5634 | -4.69 | cp | BRD-K83963101 | MLN-8054 | Aurora kinase inhibitor |
| 5623 | -4.65 | cp | BRD-K82865713 | prostaglandin-b2 | cAMP inhibitor |
| 5624 | -4.65 | cp | BRD-A52588987 | SKF-83566 | Dopamine receptor antagonist |
| 5617 | -4.62 | cp | BRD-A03216249 | mepivacaine | Potassium channel blocker |
| 5607 | -4.57 | cp | BRD-K10961822 | latanoprost | Prostanoid receptor agonist |
| 5606 | -4.56 | cp | BRD-K95921201 | reserpine | Vesicular monoamine transporter inhibitor |
| 5600 | -4.55 | cp | BRD-K96134740 | kitasamycin | Protein synthesis inhibitor |
| 5601 | -4.55 | cp | BRD-K04548931 | pidorubicine | Topoisomerase inhibitor |
| 5605 | -4.55 | cp | BRD-K22193694 | dioxybenzone | Topical sunscreen agent |
| 5594 | -4.54 | cp | BRD-A51182606 | chloramphenicol | Protein synthesis inhibitor |
| 5595 | -4.54 | cp | BRD-K40742111 | baeomycesic-acid | Lipoxygenase inhibitor |
| 5589 | -4.52 | cp | BRD-K68191783 | ALW-II-38-3 | Ephrin inhibitor |
| 5590 | -4.52 | cp | BRD-A97701745 | pindolol | Adrenergic receptor antagonist |
| 5591 | -4.52 | cp | BRD-K50859149 | sulfafurazole | Bacterial antifolate |
| 5586 | -4.51 | cp | BRD-K28907958 | CD-437 | Retinoid receptor agonist |
| 5587 | -4.51 | cp | BRD-K15502390 | nevirapine | Reverse transcriptase inhibitor |
| 5588 | -4.51 | cp | BRD-K11742128 | triprolidine | Histamine receptor antagonist |
| 5580 | -4.48 | cp | BRD-K76775527 | nimesulide | Cyclooxygenase inhibitor |
| 5576 | -4.44 | cp | BRD-K54529596 | captopril | ACE inhibitor |
| 5577 | -4.44 | cp | BRD-K59058766 | chlorprothixene | Dopamine receptor antagonist |
| 5571 | -4.42 | cp | BRD-K41567364 | SB-334867 | Orexin receptor antagonist |
| 5572 | -4.42 | cp | BRD-K32501161 | vanoxerine | Dopamine uptake inhibitor |
| 5568 | -4.39 | cp | BRD-K64310881 | MW-STK33-3B | Potassium channel activator |
| 5565 | -4.37 | cp | BRD-K68402494 | ML-9 | Myosin light chain kinase inhibitor |
| 5559 | -4.33 | cp | BRD-K08890269 | CO-102862 | Sodium channel blocker |
| 5553 | -4.27 | cp | BRD-K96720755 | relcovaptan | Vasopressin receptor antagonist |
| 5554 | -4.27 | cp | BRD-K21733600 | rofecoxib | Cyclooxygenase inhibitor |
| 5552 | -4.26 | cp | BRD-K79131256 | albendazole | Anthelmintic |
| 5547 | -4.24 | cp | BRD-A61392169 | eliprodil | Glutamate receptor antagonist |
| 5537 | -4.19 | cp | BRD-A83855350 | naltrexone | Opioid receptor antagonist |
| 5536 | -4.18 | cp | BRD-A62182663 | YK-4279 | Apoptosis stimulant |
| 5531 | -4.12 | cp | BRD-A67799922 | phenoxybenzamine | Adrenergic receptor antagonist |
| 5529 | -4.1 | cp | BRD-A04553218 | chlorphenamine | Histamine receptor antagonist |
| 5528 | -4.09 | cp | BRD-K88679075 | methandriol | Androgenic steroid |
| 5522 | -4.06 | cp | BRD-A28856712 | tetryzoline | Adrenergic receptor agonist |
| 5519 | -4.04 | cp | BRD-A94793051 | gestrinone | Progesterone receptor antagonist |
| 5511 | -4.02 | cp | BRD-K40619305 | larixinic-acid | Compound that interacts with metal centers |
| 5512 | -4.02 | cp | BRD-K62810658 | PD-98059 | MEK inhibitor |
| 5506 | -3.98 | cp | BRD-A89175223 | bisoprolol | Adrenergic receptor antagonist |
| 5499 | -3.95 | cp | BRD-K03981224 | ethisterone | Progestogen hormone |
| 5495 | -3.91 | cp | BRD-K09631521 | thiotepa | Cytochrome P450 inhibitor |
| 5489 | -3.89 | cp | BRD-A62434282 | goserelin | Gonadotropin releasing factor hormone receptor agonist |
| 5490 | -3.89 | cp | BRD-K19525698 | ozagrel | Thromboxane synthase inhibitor |
| 5485 | -3.85 | cp | BRD-A71203467 | l-stepholidine | Dopamine receptor antagonist |
| 5468 | -3.77 | cp | BRD-K20197062 | SA-94315 | Caspase inhibitor |
| 5469 | -3.77 | cp | BRD-K20338176 | cefaclor | Bacterial cell wall synthesis inhibitor |
| 5470 | -3.77 | cp | BRD-A98702003 | carbenoxolone | 11-beta-HSD1 inhibitor |
| 5463 | -3.74 | cp | BRD-A62025033 | temsirolimus | MTOR inhibitor |
| 5450 | -3.68 | cp | BRD-K51223576 | AG-99 | Tyrosine kinase inhibitor |
| 5443 | -3.66 | cp | BRD-K88304388 | dextrorphan | Glutamate receptor antagonist |
| 5444 | -3.66 | cp | BRD-K45068323 | W-13 | Calmodulin antagonist |
| 5445 | -3.66 | cp | BRD-K09497549 | kawain | Calcium channel modulator |
| 5440 | -3.65 | cp | BRD-K77771411 | moxonidine | Imidazoline receptor agonist |
| 5436 | -3.63 | cp | BRD-K37561857 | zardaverine | Phosphodiesterase inhibitor |
| 5437 | -3.63 | cp | BRD-A03359064 | ICI-89406 | Adrenergic receptor antagonist |
| 5438 | -3.63 | cp | BRD-A64125466 | dehydrocholic-acid | choleretic agent |
| 5439 | -3.63 | cp | BRD-K39823328 | VU-0366037-2 | Glutamate receptor modulator |
| 5434 | -3.62 | cp | BRD-K76617868 | fasudil | Rho associated kinase inhibitor |
| 5428 | -3.59 | cp | BRD-A12560204 | nitrendipine | Calcium channel blocker |
| 5423 | -3.56 | cp | BRD-A17655518 | ibuprofen | Cyclooxygenase inhibitor |
| 5424 | -3.56 | cp | BRD-K67783091 | haloperidol | Dopamine receptor antagonist |
| 5410 | -3.52 | cp | BRD-K87573634 | propylpyrazole | Estrogen receptor agonist |
| 5403 | -3.49 | cp | BRD-K57718010 | pentylenetetrazol | GABA receptor antagonist |
| 5404 | -3.49 | cp | BRD-A10977446 | carvedilol | Adrenergic receptor antagonist |
| 5400 | -3.48 | cp | BRD-K77390737 | xanthohumol | ATPase inhibitor |
| 5382 | -3.42 | cp | BRD-K61993165 | niacin | NAD precursor with lipid lowering effects |
| 5383 | -3.42 | cp | BRD-K90574421 | ipsapirone | Serotonin receptor agonist |
| 5390 | -3.42 | cp | BRD-K74763371 | bosentan | Endothelin receptor antagonist |
| 5376 | -3.38 | cp | BRD-K23623876 | decafluorobutane | Contrast agent |
| 5372 | -3.35 | cp | BRD-K18135438 | chenodeoxycholic-acid | 11-beta-HSD1 inhibitor |
| 5373 | -3.35 | cp | BRD-K41713976 | E-4031 | Potassium channel blocker |
| 5370 | -3.33 | cp | BRD-K47693913 | evoxine | Furoquinoline alkaloid |
| 5366 | -3.31 | cp | BRD-K08547377 | irinotecan | Topoisomerase inhibitor |
| 5367 | -3.31 | cp | BRD-K51313569 | palbociclib | CDK inhibitor |
| 5364 | -3.29 | cp | BRD-A78322124 | dobutamine | Adrenergic receptor agonist |
| 5359 | -3.28 | cp | BRD-K71731651 | PNU-120596 | Acetylcholine receptor agonist |
| 5349 | -3.24 | cp | BRD-K18757346 | U-46619 | Thromboxane receptor agonist |
| 5350 | -3.24 | cp | BRD-M00539986 | formoterol | Adrenergic receptor agonist |
| 5334 | -3.17 | cp | BRD-K69840642 | ISOX | HDAC inhibitor |
| 5335 | -3.17 | cp | BRD-A09056319 | alfuzosin | Adrenergic receptor antagonist |
| 5331 | -3.14 | cp | BRD-K24538644 | KUC104502N | -666 |
| 5332 | -3.14 | cp | BRD-A30815329 | felodipine | Calcium channel blocker |
| 5326 | -3.1 | cp | BRD-K48300629 | zonisamide | Sodium channel blocker |
| 5323 | -3.07 | cp | BRD-K00603606 | ticlopidine | Purinergic receptor antagonist |
| 5319 | -3.06 | cp | BRD-K42142750 | retrorsine | Antimitotic |
| 5312 | -3.05 | cp | BRD-K56745457 | azauridine | Antiviral |
| 5313 | -3.05 | cp | BRD-A09828896 | SKF-81297 | Dopamine receptor agonist |
| 5306 | -3.03 | cp | BRD-A52172093 | VU-0413807-2 | Calcium channel blocker |
| 5307 | -3.03 | cp | BRD-K62996583 | lidoflazine | Calcium channel blocker |
| 5304 | -3 | cp | BRD-A31800922 | procyclidine | Acetylcholine receptor antagonist |
| 5301 | -2.98 | cp | BRD-K01815685 | indole | aryl hydrocarbon receptor agonist |
| 5300 | -2.96 | cp | BRD-K74430258 | 1,2-dichlorobenzene | Hepatotoxicant |
| 5291 | -2.94 | cp | BRD-K00532621 | midazolam | Benzodiazepine receptor agonist |
| 5290 | -2.93 | cp | BRD-K19227686 | phenolphthalein | Indicator dye |
| 5284 | -2.92 | cp | BRD-K16621777 | enobosarm | Androgen receptor modulator |
| 5285 | -2.92 | cp | BRD-K66019333 | oxantel | Anthelmintic |
| 5286 | -2.92 | cp | BRD-K28307902 | flutamide | Androgen receptor antagonist |
| 5278 | -2.89 | cp | BRD-K21520694 | sulfacetamide | PABA antagonist |
| 5266 | -2.85 | cp | BRD-K34508425 | KUC103898N | -666 |
| 5267 | -2.85 | cp | BRD-A66927094 | nemonapride | Dopamine receptor antagonist |
| 5268 | -2.85 | cp | BRD-K95202259 | ML-3163 | p38 MAPK inhibitor |
| 5271 | -2.85 | cp | BRD-A92585442 | RU-28318 | Cytochrome P450 inhibitor |
| 5259 | -2.83 | cp | BRD-K89274813 | DMP-543 | Acetylcholine release stimulant |
| 5260 | -2.83 | cp | BRD-A28318179 | aminomethyltransferase | Nitric oxide synthase inhibitor |
| 5254 | -2.82 | cp | BRD-K37720887 | SB-525334 | TGF beta receptor inhibitor |
| 5255 | -2.82 | cp | BRD-K73589491 | nizatidine | Histamine receptor antagonist |
| 5258 | -2.82 | cp | BRD-K29458283 | chlorambucil | DNA inhibitor |
| 5240 | -2.79 | cp | BRD-K06753942 | nobiletin | MEK inhibitor |
| 5241 | -2.79 | cp | BRD-K39987650 | bisacodyl | Laxative |
| 5236 | -2.78 | cp | BRD-K64994968 | progesterone | Progesterone receptor agonist |
| 5237 | -2.78 | cp | BRD-K40656405 | L-165041 | PPAR receptor agonist |
| 5234 | -2.76 | cp | BRD-A31195449 | TCB2 | Serotonin receptor agonist |
| 5233 | -2.75 | cp | BRD-K28346421 | rifapentine | RNA polymerase inhibitor |
| 5222 | -2.71 | cp | BRD-K47761761 | PD-168077 | Dopamine receptor agonist |
| 5223 | -2.71 | cp | BRD-K65639003 | icariin | Phosphodiesterase inhibitor |
| 5212 | -2.68 | cp | BRD-K73589401 | corticosterone | mineralocorticoid receptor agonist |
| 5206 | -2.64 | cp | BRD-K98684188 | GSK-0660 | PPAR receptor antagonist |
| 5207 | -2.64 | cp | BRD-A64227845 | SKF-77434 | Dopamine receptor agonist |
| 5201 | -2.62 | cp | BRD-A28970875 | puromycin | Protein synthesis inhibitor |
| 5197 | -2.61 | cp | BRD-K78280988 | anandamide | Cannabinoid receptor agonist |
| 5185 | -2.58 | cp | BRD-K41260949 | valproic-acid | HDAC inhibitor |
| 5182 | -2.57 | cp | BRD-K13927029 | retinol | Retinoid receptor ligand |
| 5183 | -2.57 | cp | BRD-K44067360 | flufenamic-acid | Chloride channel blocker |
| 5184 | -2.57 | cp | BRD-K15563106 | phloretin | Sodium/glucose cotransporter inhibitor |
| 5181 | -2.56 | cp | BRD-K19136521 | indirubin | CDK inhibitor |
| 5171 | -2.54 | cp | BRD-K50018155 | RS-67506 | Serotonin receptor partial agonist |
| 5172 | -2.54 | cp | BRD-K32318651 | acyclovir | DNA polymerase inhibitor |
| 5161 | -2.49 | cp | BRD-K78692225 | leflunomide | Dihydroorotate dehydrogenase inhibitor |
| 5156 | -2.47 | cp | BRD-K00486786 | RO-08-2750 | NGF binding inhibitor |
| 5155 | -2.46 | cp | BRD-A33168282 | sotalol | Adrenergic receptor antagonist |
| 5149 | -2.45 | cp | BRD-A94413429 | NTNCB | Neuropeptide receptor antagonist |
| 5148 | -2.44 | cp | BRD-K32821942 | azathioprine | Dehydrogenase inhibitor |
| 5142 | -2.43 | cp | BRD-K87158025 | benzamil | Sodium channel blocker |
| 5143 | -2.43 | cp | BRD-K60770992 | pergolide | Dopamine receptor agonist |
| 5129 | -2.4 | cp | BRD-K11663430 | pyroxamide | HDAC inhibitor |
| 5130 | -2.4 | cp | BRD-K30020243 | aliskiren | Antihypertensive |
| 5122 | -2.37 | cp | BRD-K65331431 | retinyl | vitamin analog |
| 5115 | -2.36 | cp | BRD-K12260308 | xanthoxyline | Antifungal |
| 5121 | -2.36 | cp | BRD-K29950728 | clomifene | Estrogen receptor antagonist |
| 5104 | -2.33 | cp | BRD-K70557564 | zosuquidar | P-glycoprotein inhibitor |
| 5105 | -2.33 | cp | BRD-K54790157 | trioxsalen | DNA synthesis inhibitor |
| 5106 | -2.33 | cp | BRD-A97479839 | piperidolate | Acetylcholine receptor antagonist |
| 5109 | -2.33 | cp | BRD-K38197229 | bumetanide | Solute carrier family member inhibitor |
| 5100 | -2.32 | cp | BRD-K28143534 | cyproheptadine | Histamine receptor antagonist |
| 5090 | -2.29 | cp | BRD-K93325701 | damnacanthal | SRC inhibitor |
| 5091 | -2.29 | cp | BRD-K42679050 | Y-27152 | Potassium channel activator |
| 5088 | -2.27 | cp | BRD-K86434416 | selegiline | Monoamine oxidase inhibitor |
| 5081 | -2.26 | cp | BRD-K27710560 | splitomycin | SIRT inhibitor |
| 5073 | -2.22 | cp | BRD-K18816859 | L-694247 | Serotonin receptor agonist |
| 5076 | -2.22 | cp | BRD-K68264559 | brimonidine | Adrenergic receptor agonist |
| 5067 | -2.21 | cp | BRD-U08759356 | EI-346-erlotinib-analog | EGFR inhibitor |
| 5065 | -2.18 | cp | BRD-K40901640 | cinanserin | Serotonin receptor antagonist |
| 5051 | -2.15 | cp | BRD-K04196797 | oxcarbazepine | Sodium channel blocker |
| 5052 | -2.15 | cp | BRD-A22684332 | procaterol | Adrenergic receptor agonist |
| 5053 | -2.15 | cp | BRD-A16694057 | bisphenol-a | PPAR receptor antagonist |
| 5054 | -2.15 | cp | BRD-K93080877 | Ala-Ala-Phe-CMK | Tripeptidyl peptidase inhibitor |
| 5055 | -2.15 | cp | BRD-A84481105 | thioridazine | Dopamine receptor antagonist |
| 5041 | -2.13 | cp | BRD-A84134924 | pancuronium | Acetylcholine receptor antagonist |
| 5042 | -2.13 | cp | BRD-A88282067 | delcorine | Antiarrhythmic |
| 5036 | -2.11 | cp | BRD-K68507560 | dicycloverine | Acetylcholine receptor antagonist |
| 5037 | -2.11 | cp | BRD-A81233518 | glycopyrrolate | Acetylcholine receptor antagonist |
| 5038 | -2.11 | cp | BRD-K86600316 | RS-79948 | Adrenergic receptor antagonist |
| 5032 | -2.09 | cp | BRD-A31227688 | kynuramine | Aryl hydrocarbon receptor activator |
| 5025 | -2.08 | cp | BRD-K19462402 | buflomedil | Adrenergic receptor antagonist |
| 5011 | -2.04 | cp | BRD-K37814297 | acepromazine | Dopamine receptor antagonist |
| 5012 | -2.04 | cp | BRD-K89687904 | PKCbeta-inhibitor | PKC inhibitor |
| 5016 | -2.04 | cp | BRD-K31553034 | zibotentan | Endothelin receptor antagonist |
| 5005 | -2.03 | cp | BRD-A43974575 | tranylcypromine | Monoamine oxidase inhibitor |
| 5001 | -2.01 | cp | BRD-A93477898 | PETCM | Caspase activator |
| 5002 | -2.01 | cp | BRD-K20482099 | rutin | Antioxidant |
| 5000 | -1.99 | cp | BRD-K22031190 | diflunisal | Prostanoid receptor antagonist |
| 4989 | -1.97 | cp | BRD-A56245458 | reichstein | Androgen receptor antagonist |
| 4990 | -1.97 | cp | BRD-K85266041 | DNQX | Glutamate receptor antagonist |
| 4991 | -1.97 | cp | BRD-K04976539 | aminogenistein | SRC inhibitor |
| 4992 | -1.97 | cp | BRD-K04210847 | tamoxifen | Estrogen receptor antagonist |
| 4993 | -1.97 | cp | BRD-A91866971 | SQ-29548 | Thromboxane receptor antagonist |
| 4996 | -1.97 | cp | BRD-K77925998 | quipazine | Serotonin receptor agonist |
| 4987 | -1.96 | cp | BRD-K99595596 | salsolinol | Monoamine oxidase inhibitor |
| 4986 | -1.95 | cp | BRD-K41143549 | BRD-K41143549 | Glutamate receptor antagonist |
| 4983 | -1.94 | cp | BRD-K70327191 | benzoxiquine | Anti-infective |
| 4974 | -1.9 | cp | BRD-K05181463 | L-741626 | Dopamine receptor antagonist |
| 4975 | -1.9 | cp | BRD-K95851186 | CGP-13501 | GABA receptor modulator |
| 4958 | -1.87 | cp | BRD-K22947005 | dexbrompheniramine | Histamine receptor antagonist |
| 4959 | -1.87 | cp | BRD-A00520476 | otenzepad | Acetylcholine receptor antagonist |
| 4960 | -1.87 | cp | BRD-A62035778 | scopolamine | Acetylcholine receptor antagonist |
| 4965 | -1.87 | cp | BRD-A30435184 | metergoline | Dopamine receptor agonist |
| 4942 | -1.83 | cp | BRD-K19533706 | tranilast | Angiogenesis inhibitor |
| 4943 | -1.83 | cp | BRD-K25875056 | SC-9 | Protein tyrosine kinase activator |
| 4944 | -1.83 | cp | BRD-K94689771 | pinocembrin | CYP1B1 inhibitor |
| 4937 | -1.82 | cp | BRD-A79903587 | tegafur | Thymidylate synthase inhibitor |
| 4926 | -1.8 | cp | BRD-K64835161 | BRD-K64835161 | -666 |
| 4927 | -1.8 | cp | BRD-K34330170 | rotenonic-acid | Retinoid receptor antagonist |
| 4928 | -1.8 | cp | BRD-A10303790 | talampicillin | Bacterial cell wall synthesis inhibitor |
| 4929 | -1.8 | cp | BRD-K38251852 | paxilline | Potassium channel blocker |
| 4925 | -1.79 | cp | BRD-K54256913 | MK-1775 | WEE1 kinase inhibitor |
| 4922 | -1.77 | cp | BRD-K26134695 | calpeptin | Calpain inhibitor |
| 4917 | -1.76 | cp | BRD-K26674531 | GR-235 | Estrogen receptor agonist |
| 4918 | -1.76 | cp | BRD-K92984783 | melperone | Serotonin receptor antagonist |
| 4919 | -1.76 | cp | BRD-K07888196 | tyrphostin-AG-538 | IGF-1 inhibitor |
| 4920 | -1.76 | cp | BRD-K41707108 | ceramide | Phosphoenolpyruvate carboxylase activator |
| 4908 | -1.74 | cp | BRD-K43796186 | benzyl-quinazolin-4-yl-amine | EGFR inhibitor |
| 4905 | -1.73 | cp | BRD-A02481876 | importazole | Importin-beta transport receptor inhibitor |
| 4906 | -1.73 | cp | BRD-K73109821 | diazoxide | Potassium channel activator |
| 4903 | -1.72 | cp | BRD-K61192372 | capecitabine | DNA synthesis inhibitor |
| 4891 | -1.69 | cp | BRD-A49734948 | foliosidine | Plant alkaloid |
| 4892 | -1.69 | cp | BRD-K27499107 | carbacyclin | IP receptor activator |
| 4893 | -1.69 | cp | BRD-A09467419 | mebeverine | Acetylcholine receptor antagonist |
| 4877 | -1.66 | cp | BRD-A17448384 | beclometasone | Glucocorticoid receptor agonist |
| 4878 | -1.66 | cp | BRD-A26334849 | propafenone | Antiarrhythmic |
| 4879 | -1.66 | cp | BRD-K28806945 | L-750667 | Dopamine receptor antagonist |
| 4875 | -1.65 | cp | BRD-K47943470 | tyrphostin-51 | EGFR inhibitor |
| 4867 | -1.62 | cp | BRD-K07265709 | razoxane | Chelating agent |
| 4868 | -1.62 | cp | BRD-K34437622 | BRD-K34437622 | Thymidylate synthase inhibitor |
| 4869 | -1.62 | cp | BRD-A00267231 | hemado | Adenosine receptor agonist |
| 4871 | -1.62 | cp | BRD-K93918653 | quizartinib | FLT3 inhibitor |
| 4872 | -1.62 | cp | BRD-K50398167 | meclofenamic-acid | Cyclooxygenase inhibitor |
| 4859 | -1.6 | cp | BRD-A99177642 | deltaline | Acetylcholine receptor antagonist |
| 4852 | -1.59 | cp | BRD-K97799481 | theophylline | Adenosine receptor antagonist |
| 4853 | -1.59 | cp | BRD-K73838513 | cinacalcet | Calcium channel activator |
| 4854 | -1.59 | cp | BRD-A63667919 | methylergometrine | Dopamine receptor antagonist |
| 4855 | -1.59 | cp | BRD-K64755930 | etazolate | Phosphodiesterase inhibitor |
| 4856 | -1.59 | cp | BRD-K50464341 | berbamine | Calmodulin antagonist |
| 4857 | -1.59 | cp | BRD-A05186015 | bupropion | Dopamine uptake inhibitor |
| 4858 | -1.59 | cp | BRD-A19500257 | geldanamycin | HSP inhibitor |
| 4839 | -1.56 | cp | BRD-K83972459 | JWE-035 | Aurora kinase inhibitor |
| 4834 | -1.55 | cp | BRD-K70281171 | U-99194 | Dopamine receptor antagonist |
| 4835 | -1.55 | cp | BRD-K69195780 | NAN-190 | Serotonin receptor agonist |
| 4836 | -1.55 | cp | BRD-A09062839 | amylocaine | Local anesthetic |
| 4832 | -1.54 | cp | BRD-K95739795 | tetrabenazine | Vesicular monoamine transporter inhibitor |
| 4828 | -1.52 | cp | BRD-K40902647 | vincamine | Adrenergic receptor antagonist |
| 4827 | -1.51 | cp | BRD-K28470988 | L-690330 | Inositol monophosphatase inhibitor |
| 4825 | -1.48 | cp | BRD-K01567962 | pyrazolanthrone | JNK inhibitor |
| 4826 | -1.48 | cp | BRD-K00662280 | CL-218872 | GABA receptor agonist |
| 4813 | -1.44 | cp | BRD-A60294240 | tribenoside | Anti-inflammatory |
| 4815 | -1.44 | cp | BRD-K20655524 | mefexamide | Psychoactive drug |
| 4806 | -1.43 | cp | BRD-A70514680 | articaine | Local anesthetic |
| 4807 | -1.43 | cp | BRD-K70976396 | cefoxitin | Bacterial cell wall synthesis inhibitor |
| 4797 | -1.41 | cp | BRD-K90789829 | nefazodone | Adrenergic inhibitor |
| 4798 | -1.41 | cp | BRD-K94441233 | mevastatin | HMGCR inhibitor |
| 4800 | -1.41 | cp | BRD-A86044036 | flurbiprofen | Cyclooxygenase inhibitor |
| 4801 | -1.41 | cp | BRD-K72703948 | ZM-447439 | Aurora kinase inhibitor |
| 4802 | -1.41 | cp | BRD-K95609758 | BMS-191011 | Potassium channel activator |
| 4796 | -1.4 | cp | BRD-A51820102 | econazole | Bacterial cell wall synthesis inhibitor |
| 4786 | -1.38 | cp | BRD-K36864847 | BD-1047 | Adrenergic receptor antagonist |
| 4781 | -1.37 | cp | BRD-A39052811 | mosapride | Serotonin receptor agonist |
| 4782 | -1.37 | cp | BRD-K31471398 | dihydrexidine | Dopamine receptor agonist |
| 4783 | -1.37 | cp | BRD-K02275692 | cefotiam | Bacterial cell wall synthesis inhibitor |
| 4784 | -1.37 | cp | BRD-K62929068 | 6-benzylaminopurine | Purinergic receptor activator |
| 4779 | -1.35 | cp | BRD-K05977355 | fluconazole | Sterol demethylase inhibitor |
| 4767 | -1.34 | cp | BRD-A91452556 | estradiol-cypionate | Estrogen receptor agonist |
| 4768 | -1.34 | cp | BRD-K45252063 | clofibrate | PPAR receptor agonist |
| 4762 | -1.33 | cp | BRD-K16977723 | PP-3 | EGFR inhibitor |
| 4757 | -1.32 | cp | BRD-K23566484 | nilutamide | Androgen receptor antagonist |
| 4755 | -1.31 | cp | BRD-K14329163 | BAY-K8644 | Calcium channel activator |
| 4749 | -1.3 | cp | BRD-A59174698 | ritodrine | Adrenergic receptor agonist |
| 4750 | -1.3 | cp | BRD-K74141488 | naftifine | Fungal squalene epoxidase inhibitor |
| 4751 | -1.3 | cp | BRD-K59851896 | calycanthine | GABA release inhibitor |
| 4754 | -1.3 | cp | BRD-K37289225 | clozapine | Dopamine receptor antagonist |
| 4745 | -1.29 | cp | BRD-K43405658 | tyrphostin-AG-527 | Protein tyrosine kinase inhibitor |
| 4743 | -1.28 | cp | BRD-K74112339 | acetohydroxamic-acid | Urease inhibitor |
| 4737 | -1.27 | cp | BRD-K92093830 | doxorubicin | Topoisomerase inhibitor |
| 4738 | -1.27 | cp | BRD-K38003476 | clocortolone | Glucocorticoid receptor agonist |
| 4739 | -1.27 | cp | BRD-K76304753 | phenazopyridine | Local anesthetic |
| 4740 | -1.27 | cp | BRD-K06335600 | tizanidine | Adrenergic receptor agonist |
| 4741 | -1.27 | cp | BRD-K85119730 | tolbutamide | ATP channel blocker |
| 4742 | -1.27 | cp | BRD-K09778810 | FGIN-1-27 | Inositol monophosphatase inhibitor |
| 4731 | -1.26 | cp | BRD-A06726973 | dibutyrylcyclic-gmp | cGMP analog |
| 4728 | -1.25 | cp | BRD-K70778732 | trazodone | Adrenergic receptor antagonist |
| 4715 | -1.23 | cp | BRD-K24859147 | KIN001-242 | Protein kinase inhibitor |
| 4716 | -1.23 | cp | BRD-K39462424 | dexchlorpheniramine | Histamine receptor antagonist |
| 4717 | -1.23 | cp | BRD-K99063460 | didanosine | Nucleoside reverse transcriptase inhibitor |
| 4718 | -1.23 | cp | BRD-K67277431 | picotamide | Thromboxane receptor antagonist |
| 4719 | -1.23 | cp | BRD-K08998509 | fananserin | Dopamine receptor antagonist |
| 4720 | -1.23 | cp | BRD-K49945136 | GR-113808 | Serotonin receptor antagonist |
| 4721 | -1.23 | cp | BRD-A02180903 | betamethasone | Glucocorticoid receptor agonist |
| 4702 | -1.2 | cp | BRD-A79465854 | auranofin | NFkB pathway inhibitor |
| 4703 | -1.2 | cp | BRD-K12932420 | YM-976 | Phosphodiesterase inhibitor |
| 4704 | -1.2 | cp | BRD-K36638830 | anabasine | Acetylcholine receptor agonist |
| 4705 | -1.2 | cp | BRD-A54880345 | etomidate | GABA receptor modulator |
| 4706 | -1.2 | cp | BRD-K49049886 | CGS-15943 | Adenosine receptor antagonist |
| 4709 | -1.2 | cp | BRD-K32164935 | tolazamide | ATP channel blocker |
| 4710 | -1.2 | cp | BRD-K82746043 | navitoclax | BCL inhibitor |
| 4688 | -1.16 | cp | BRD-K90733503 | cefalexin | Bacterial cell wall synthesis inhibitor |
| 4689 | -1.16 | cp | BRD-K87024524 | phenelzine | Monoamine oxidase inhibitor |
| 4690 | -1.16 | cp | BRD-K29555132 | arachidonamide | Cannabinoid receptor agonist |
| 4691 | -1.16 | cp | BRD-K91904471 | SD-169 | p38 MAPK inhibitor |
| 4692 | -1.16 | cp | BRD-K28936863 | ketotifen | Histamine receptor agonist |
| 4698 | -1.16 | cp | BRD-K07691486 | roscovitine | CDK inhibitor |
| 4678 | -1.13 | cp | BRD-K71003802 | hippeastrine | Plant alkaloid |
| 4679 | -1.13 | cp | BRD-K79353516 | indolophenanthridine | CALY activator |
| 4680 | -1.13 | cp | BRD-K81876028 | CP-93129 | Serotonin receptor agonist |
| 4684 | -1.13 | cp | BRD-K12102668 | nialamide | Monoamine oxidase inhibitor |
| 4685 | -1.13 | cp | BRD-K47983010 | BX-795 | IKK inhibitor |
| 4677 | -1.12 | cp | BRD-K00615600 | AG-14361 | PARP inhibitor |
| 4667 | -1.1 | cp | BRD-K79404599 | enzastaurin | PKC inhibitor |
| 4668 | -1.1 | cp | BRD-K05395900 | nicotine | Acetylcholine receptor agonist |
| 4660 | -1.09 | cp | BRD-K52721684 | PCO-400 | Potassium channel activator |
| 4661 | -1.09 | cp | BRD-A75455249 | kavain | Calcium channel modulator |
| 4662 | -1.09 | cp | BRD-K85013741 | auraptene | Nitric oxide production inhibitor |
| 4663 | -1.09 | cp | BRD-K93441486 | diphemanil | Acetylcholine receptor antagonist |
| 4664 | -1.09 | cp | BRD-K98530306 | clonidine | Adrenergic receptor agonist |
| 4651 | -1.06 | cp | BRD-K96471533 | nitazoxanide | Pyruvate ferredoxin oxidoreductase inhibitor |
| 4652 | -1.06 | cp | BRD-K47278471 | diphenhydramine | Histamine receptor antagonist |
| 4653 | -1.06 | cp | BRD-K54094468 | remoxipride | Dopamine receptor antagonist |
| 4654 | -1.06 | cp | BRD-K87048468 | RS-102221 | Serotonin receptor antagonist |
| 4655 | -1.06 | cp | BRD-K02283807 | GR-32191 | Thromboxane receptor antagonist |
| 4656 | -1.06 | cp | BRD-K30480208 | torasemide | Electrolyte reabsorption inhibitor |
| 4657 | -1.06 | cp | BRD-A22769835 | homochlorcyclizine | Antihistamine |
| 4658 | -1.06 | cp | BRD-K83023055 | GR-135531 | Melatonin receptor agonist |
| 4647 | -1.04 | cp | BRD-K71534238 | GW-9508 | Free fatty acid receptor agonist |
| 4639 | -1.02 | cp | BRD-K85503079 | perospirone | Dopamine receptor antagonist |
| 4640 | -1.02 | cp | BRD-K81169441 | cerivastatin | HMGCR inhibitor |
| 4641 | -1.02 | cp | BRD-K63195589 | tipifarnib | Farnesyltransferase inhibitor |
| 4642 | -1.02 | cp | BRD-K54142781 | cirazoline | Adrenergic receptor agonist |
| 4645 | -1.02 | cp | BRD-A24514565 | warfarin | Vitamin K antagonist |
| 4630 | -0.99 | cp | BRD-K32795028 | 1-benzylimidazole | Thromboxane synthase inhibitor |
| 4631 | -0.99 | cp | BRD-A19633847 | perhexiline | Carnitine palmitoyltransferase inhibitor |
| 4632 | -0.99 | cp | BRD-A63043573 | cabergoline | Dopamine receptor agonist |
| 4628 | -0.98 | cp | BRD-K99447003 | enalaprilat | ACE inhibitor |
| 4629 | -0.98 | cp | BRD-K33818169 | GW-3965 | LXR agonist |
| 4616 | -0.95 | cp | BRD-K67174588 | toremifene | Estrogen receptor antagonist |
| 4617 | -0.95 | cp | BRD-A18202423 | CPCCOEt | Glutamate receptor antagonist |
| 4618 | -0.95 | cp | BRD-K51662849 | ilomastat | Matrix metalloprotease inhibitor |
| 4619 | -0.95 | cp | BRD-K77947974 | fluspirilene | Dopamine receptor antagonist |
| 4609 | -0.92 | cp | BRD-K53570330 | carbofuran | Cholinesterase inhibitor |
| 4596 | -0.88 | cp | BRD-K68065987 | MK-2206 | AKT inhibitor |
| 4597 | -0.88 | cp | BRD-A71262238 | nafadotride | Dopamine receptor antagonist |
| 4598 | -0.88 | cp | BRD-K39670393 | amthamine | Histamine receptor agonist |
| 4599 | -0.88 | cp | BRD-A11678676 | wortmannin | PI3K inhibitor |
| 4600 | -0.88 | cp | BRD-A24817035 | laudanosine | Central nervous system agent |
| 4604 | -0.88 | cp | BRD-K38055836 | etamivan | Respiratory stimulant |
| 4587 | -0.85 | cp | BRD-K50938786 | ropivacaine | Sodium channel blocker |
| 4588 | -0.85 | cp | BRD-A42423104 | benproperine | Antitussive |
| 4589 | -0.85 | cp | BRD-K50325075 | UCL-2077 | Slow after hyperpolarization channel blocker |
| 4590 | -0.85 | cp | BRD-K68873215 | phosphodiesterase-V-inhibitor-II | Phosphodiesterase inhibitor |
| 4591 | -0.85 | cp | BRD-A32949107 | MRS-1845 | Calcium channel blocker |
| 4592 | -0.85 | cp | BRD-A95939040 | sertaconazole | Sterol demethylase inhibitor |
| 4574 | -0.81 | cp | BRD-A93659613 | GR-89696 | Opioid receptor agonist |
| 4575 | -0.81 | cp | BRD-K86509404 | iso-olomoucine | CDK inhibitor |
| 4573 | -0.8 | cp | BRD-K15164005 | apoptosis-activator-II | Carboxylesterase inhibitor |
| 4567 | -0.79 | cp | BRD-K64610608 | EMF-bca1-57 | caspase inhibitor |
| 4563 | -0.78 | cp | BRD-K68407802 | KIN001-055 | EGFR inhibitor |
| 4564 | -0.78 | cp | BRD-K80738081 | resveratrol | Cytochrome P450 inhibitor |
| 4555 | -0.75 | cp | BRD-K86191271 | cytosporone-b | NUR77 receptor agonist |
| 4542 | -0.74 | cp | BRD-K56957086 | dacinostat | HDAC inhibitor |
| 4543 | -0.74 | cp | BRD-K61314889 | IWR-1-ENDO | PARP inhibitor |
| 4544 | -0.74 | cp | BRD-K70487031 | flupentixol | Dopamine receptor antagonist |
| 4545 | -0.74 | cp | BRD-A44133049 | azasetron | Serotonin receptor antagonist |
| 4546 | -0.74 | cp | BRD-A41833852 | naloxone | Opioid receptor antagonist |
| 4547 | -0.74 | cp | BRD-K43860855 | iobenguane | Antineoplastic |
| 4548 | -0.74 | cp | BRD-K18909381 | CGS-12066B | Serotonin receptor agonist |
| 4549 | -0.74 | cp | BRD-K10870738 | CDC | Lipoxygenase inhibitor |
| 4550 | -0.74 | cp | BRD-K25905511 | buddleoflavonoloside | Acetylcholinesterase inhibitor |
| 4551 | -0.74 | cp | BRD-K86301799 | dipyridamole | Phosphodiesterase inhibitor |
| 4532 | -0.7 | cp | BRD-K46742498 | alosetron | Serotonin receptor antagonist |
| 4533 | -0.7 | cp | BRD-K15791587 | L-733060 | Tachykinin antagonist |
| 4534 | -0.7 | cp | BRD-K89402695 | L-655240 | Thromboxane receptor antagonist |
| 4535 | -0.7 | cp | BRD-K84663978 | trequinsin | Phosphodiesterase inhibitor |
| 4536 | -0.7 | cp | BRD-A31204924 | mitotane | Antineoplastic |
| 4524 | -0.67 | cp | BRD-A31007383 | SDZ-WAG-994 | Adenosine receptor agonist |
| 4525 | -0.67 | cp | BRD-K32696739 | noreleagnine | Monoamine oxidase inhibitor |
| 4526 | -0.67 | cp | BRD-A16332958 | modafinil | Adrenergic receptor agonist |
| 4527 | -0.67 | cp | BRD-K39746403 | erythromycin | NFkB pathway inhibitor |
| 4528 | -0.67 | cp | BRD-K99922388 | DPO-1 | Potassium channel blocker |
| 4529 | -0.67 | cp | BRD-A75769826 | SDM25N | Opioid receptor antagonist |
| 4518 | -0.63 | cp | BRD-K94270326 | ecopipam | Dopamine receptor antagonist |
| 4519 | -0.63 | cp | BRD-K77171813 | proxyfan | Histamine receptor modulator |
| 4520 | -0.63 | cp | BRD-K13261168 | LY-16350 | Dopamine receptor agonist |
| 4521 | -0.63 | cp | BRD-K32292990 | CGP-53353 | EGFR inhibitor |
| 4522 | -0.63 | cp | BRD-K48735772 | PD-158780 | EGFR inhibitor |
| 4516 | -0.62 | cp | BRD-A43940795 | tetrahydropalmatine | Serotonin release inhibitor |
| 4517 | -0.62 | cp | BRD-K53561341 | KIN001-220 | Aurora kinase inhibitor |
| 4515 | -0.61 | cp | BRD-A08877921 | cephalotaxine | Protein synthesis inhibitor |
| 4509 | -0.6 | cp | BRD-K39965020 | doconexent | PPAR receptor agonist |
| 4510 | -0.6 | cp | BRD-K43068349 | AMG-9810 | TRPV antagonist |
| 4514 | -0.6 | cp | BRD-A10523515 | GSK-429286A | Rho associated kinase inhibitor |
| 4507 | -0.59 | cp | BRD-A50684349 | fenoldopam | Dopamine receptor agonist |
| 4502 | -0.56 | cp | BRD-A10903566 | imiloxan | Adrenergic receptor antagonist |
| 4503 | -0.56 | cp | BRD-K53737926 | amitriptyline | Norepinephrine inhibitor |
| 4490 | -0.53 | cp | BRD-K68202742 | trichostatin-a | HDAC inhibitor |
| 4491 | -0.53 | cp | BRD-K02227374 | milnacipran | Serotonin reuptake inhibitor |
| 4492 | -0.53 | cp | BRD-K76908866 | CP-724714 | EGFR inhibitor |
| 4493 | -0.53 | cp | BRD-K32107296 | temozolomide | DNA alkylating agent |
| 4494 | -0.53 | cp | BRD-K13646352 | midostaurin | FLT3 inhibitor |
| 4495 | -0.53 | cp | BRD-A99449986 | MT-21 | Caspase activator |
| 4496 | -0.53 | cp | BRD-K80451230 | zamifenacin | Acetylcholine receptor antagonist |
| 4500 | -0.53 | cp | BRD-A48430263 | pioglitazone | Insulin sensitizer |
| 4501 | -0.53 | cp | BRD-A35588707 | teniposide | Topoisomerase inhibitor |
| 4473 | -0.49 | cp | BRD-A11605036 | thiocolchicoside | GABA receptor antagonist |
| 4474 | -0.49 | cp | BRD-A21723284 | naltriben | Opioid receptor antagonist |
| 4475 | -0.49 | cp | BRD-K06014311 | DH-97 | Melatonin receptor antagonist |
| 4476 | -0.49 | cp | BRD-A46393198 | tetramisole | Immunostimulant |
| 4477 | -0.49 | cp | BRD-K36862742 | hydroflumethiazide | Sodium/potassium/chloride transporter inhibitor |
| 4478 | -0.49 | cp | BRD-A29734509 | disopyramide | Sodium channel blocker |
| 4460 | -0.46 | cp | BRD-K63343048 | orlistat | Lipase inhibitor |
| 4461 | -0.46 | cp | BRD-K23383398 | T-0901317 | LXR agonist |
| 4462 | -0.46 | cp | BRD-K04414442 | SB-222200 | Tachykinin antagonist |
| 4463 | -0.46 | cp | BRD-A41451487 | PK-11195 | Benzodiazepine receptor antagonist |
| 4464 | -0.46 | cp | BRD-K66707493 | lawsone | Coloring agent |
| 4465 | -0.46 | cp | BRD-K93480852 | KN-93 | Calcium-calmodulin dependent protein kinase inhibitor |
| 4466 | -0.46 | cp | BRD-A92537424 | danazol | Estrogen receptor antagonist |
| 4467 | -0.46 | cp | BRD-K49448285 | bisindolylmaleimide | CDK inhibitor |
| 4469 | -0.46 | cp | BRD-K10995081 | perphenazine | Dopamine receptor antagonist |
| 4470 | -0.46 | cp | BRD-K79254416 | decitabine | DNA methyltransferase inhibitor |
| 4471 | -0.46 | cp | BRD-K78126613 | menadione | Mitochondrial DNA polymerase inhibitor |
| 4446 | -0.42 | cp | BRD-K66874953 | pifithrin-alpha | TP53 inhibitor |
| 4447 | -0.42 | cp | BRD-A44780397 | mifepristone | Glucocorticoid receptor antagonist |
| 4448 | -0.42 | cp | BRD-K68558722 | deracoxib | Cyclooxygenase inhibitor |
| 4450 | -0.42 | cp | BRD-K53857191 | risperidone | Dopamine receptor antagonist |
| 4451 | -0.42 | cp | BRD-A31159102 | fluoxetine | Selective serotonin reuptake inhibitor (SSRI) |
| 4452 | -0.42 | cp | BRD-K61250553 | loperamide | Opioid receptor agonist |
| 4438 | -0.39 | cp | BRD-K68437527 | EMF-bca1-60 | caspase inhibitor |
| 4439 | -0.39 | cp | BRD-K11163873 | phenanthridone | PARP inhibitor |
| 4440 | -0.39 | cp | BRD-K98769987 | flumazenil | Benzodiazepine receptor antagonist |
| 4441 | -0.39 | cp | BRD-K32645441 | dipropyl-5ct | Serotonin receptor agonist |
| 4442 | -0.39 | cp | BRD-K17561142 | amiodarone | Potassium channel blocker |
| 4443 | -0.39 | cp | BRD-K19540840 | saracatinib | SRC inhibitor |
| 4428 | -0.35 | cp | BRD-A92630576 | trimebutine | Opioid receptor agonist |
| 4429 | -0.35 | cp | BRD-K84955386 | hyperoside | Glucosidase inhibitor |
| 4430 | -0.35 | cp | BRD-K47717570 | NBQX | Glutamate receptor antagonist |
| 4431 | -0.35 | cp | BRD-K57222227 | indometacin | Cyclooxygenase inhibitor |
| 4432 | -0.35 | cp | BRD-A29485665 | bicalutamide | Androgen receptor antagonist |
| 4433 | -0.35 | cp | BRD-K77641333 | naphazoline | Adrenergic receptor agonist |
| 4420 | -0.32 | cp | BRD-A24381660 | zeranol | Estrogen receptor agonist |
| 4421 | -0.32 | cp | BRD-K62858456 | lomerizine | Calcium channel blocker |
| 4422 | -0.32 | cp | BRD-K09963420 | saquinavir | HIV protease inhibitor |
| 4425 | -0.32 | cp | BRD-K93034159 | cladribine | Adenosine deaminase inhibitor |
| 4407 | -0.28 | cp | BRD-K80725632 | lavendustin-c | EGFR inhibitor |
| 4408 | -0.28 | cp | BRD-K96402602 | farnesylthiotriazole | PPMTase inhibitor |
| 4409 | -0.28 | cp | BRD-K59522102 | piperine | Monoamine oxidase inhibitor |
| 4410 | -0.28 | cp | BRD-K59332007 | linopirdine | Potassium channel blocker |
| 4411 | -0.28 | cp | BRD-K39915878 | loxapine | Dopamine receptor antagonist |
| 4412 | -0.28 | cp | BRD-K08287586 | butylparaben | DNA synthesis inhibitor |
| 4415 | -0.28 | cp | BRD-K92049597 | triamterene | Sodium channel blocker |
| 4402 | -0.25 | cp | BRD-K09416995 | lovastatin | HMGCR inhibitor |
| 4403 | -0.25 | cp | BRD-A01643550 | prednisolone | Glucocorticoid receptor agonist |
| 4404 | -0.25 | cp | BRD-A64092382 | mexiletine | Sodium channel blocker |
| 4392 | -0.21 | cp | BRD-K23335153 | AMN-082 | Glutamate receptor modulator |
| 4393 | -0.21 | cp | BRD-K68867920 | quetiapine | Dopamine receptor antagonist |
| 4394 | -0.21 | cp | BRD-K52219182 | BRD-K52219182 | Phosphodiesterase inhibitor |
| 4395 | -0.21 | cp | BRD-K67017579 | cilostazol | Phosphodiesterase inhibitor |
| 4396 | -0.21 | cp | BRD-K12994359 | valdecoxib | Cyclooxygenase inhibitor |
| 4380 | -0.14 | cp | BRD-A01787639 | naftopidil | Adrenergic receptor antagonist |
| 4381 | -0.14 | cp | BRD-K18523449 | mestanolone | Androgenic steroid |
| 4382 | -0.14 | cp | BRD-K94920105 | DR-2313 | PARP inhibitor |
| 4383 | -0.14 | cp | BRD-K82036761 | sertraline | Serotonin receptor antagonist |
| 4385 | -0.14 | cp | BRD-K60762818 | desipramine | Tricyclic antidepressant |
| 4386 | -0.14 | cp | BRD-K89732114 | trifluoperazine | Dopamine receptor antagonist |
| 4372 | -0.11 | cp | BRD-K81528515 | nilotinib | ABL inhibitor |
| 4373 | -0.11 | cp | BRD-K33312228 | halometasone | Glucocorticoid receptor agonist |
| 4374 | -0.11 | cp | BRD-A47829399 | artesunate | DNA synthesis inhibitor |
| 4375 | -0.11 | cp | BRD-K45033733 | famciclovir | DNA polymerase inhibitor |
| 4376 | -0.11 | cp | BRD-K89839824 | raltitrexed | Thymidylate synthase inhibitor |
| 4377 | -0.11 | cp | BRD-A01320529 | salmeterol | Adrenergic receptor agonist |
| 4359 | -0.07 | cp | BRD-K99616396 | motesanib | KIT inhibitor |
| 4360 | -0.07 | cp | BRD-A45140972 | meclocycline | Bacterial 30S ribosomal subunit inhibitor |
| 4361 | -0.07 | cp | BRD-K34533029 | tyrphostin-AG-494 | EGFR inhibitor |
| 4362 | -0.07 | cp | BRD-K06712146 | YM-90709 | IL5 inhibitor |
| 4363 | -0.07 | cp | BRD-K13544237 | r(-)-propylnorapomorphine | Dopamine receptor agonist |
| 4364 | -0.07 | cp | BRD-A25234499 | aminoglutethimide | Glucocorticoid receptor antagonist |
| 4365 | -0.07 | cp | BRD-K91699951 | benzonatate | Local anesthetic |
| 4358 | -0.04 | cp | BRD-K00627859 | tubastatin-a | HDAC inhibitor |
| 4354 | -0.03 | cp | BRD-K28849549 | mesalazine | Cyclooxygenase inhibitor |
| 4355 | -0.03 | cp | BRD-K60511616 | pravastatin | HMGCR inhibitor |
| 4350 | -0.02 | cp | BRD-A34817987 | itraconazole | Cytochrome P450 inhibitor |
| 4351 | -0.02 | cp | BRD-K49111258 | prazosin | Adrenergic receptor antagonist |
| 4347 | -0.01 | cp | BRD-K13032584 | procarbazine | Monoamine oxidase inhibitor |
| 2409 | 0 | cp | BRD-K42573370 | avrainvillamide-analog-2 | nucleophosmin inhibitor |
| 2777 | 0 | cp | BRD-K94070024 | depomedrol | Glucocorticoid receptor agonist |
| 2778 | 0 | cp | BRD-K83636919 | entacapone | Catechol O methyltransferase inhibitor |
| 2779 | 0 | cp | BRD-K56450366 | NSC-94258 | Antineoplastic |
| 2780 | 0 | cp | BRD-A20589515 | dihydroxyphenylglycine | Glutamate receptor agonist |
| 2781 | 0 | cp | BRD-K54472332 | elvitegravir | HIV integrase inhibitor |
| 2782 | 0 | cp | BRD-K89014967 | AS-703026 | MEK inhibitor |
| 2783 | 0 | cp | BRD-K91900765 | VX-745 | p38 MAPK inhibitor |
| 2784 | 0 | cp | BRD-K99749624 | linifanib | PDGFR receptor inhibitor |
| 2785 | 0 | cp | BRD-K83794624 | pirarubicin | Topoisomerase inhibitor |
| 2786 | 0 | cp | BRD-K99451608 | lopinavir | HIV protease inhibitor |
| 2787 | 0 | cp | BRD-K00184207 | GR-206 | Aryl hydrocarbon receptor ligand |
| 2788 | 0 | cp | BRD-K95402279 | geranylgeraniol | Farnesyltransferase inhibitor |
| 2789 | 0 | cp | BRD-A18497530 | 5-iodotubercidin | Adenosine kinase inhibitor |
| 2790 | 0 | cp | BRD-K35128472 | 2-aminopurine | Serine/threonine kinase inhibitor |
| 2791 | 0 | cp | BRD-K32610195 | androstenedione | Cytochrome P450 inhibitor |
| 2792 | 0 | cp | BRD-K74733595 | APHA-compound-8 | HDAC inhibitor |
| 2793 | 0 | cp | BRD-A11702965 | chromomycin-a3 | DNA binding agent |
| 2794 | 0 | cp | BRD-K63915849 | AS-604850 | PI3K inhibitor |
| 2795 | 0 | cp | BRD-U86922168 | QL-XII-47 | BTK inhibitor |
| 2796 | 0 | cp | BRD-U51951544 | ZG-10 | JNK inhibitor |
| 2797 | 0 | cp | BRD-U25771771 | WZ-4-145 | EGFR inhibitor |
| 2798 | 0 | cp | BRD-K38615104 | A-443644 | AKT inhibitor |
| 2799 | 0 | cp | BRD-K19220233 | JNK-9L | JNK inhibitor |
| 2800 | 0 | cp | BRD-A60245366 | AS-601245 | JNK inhibitor |
| 2801 | 0 | cp | BRD-K04412738 | tramadol | Norepinephrine reuptake inhibitor |
| 2802 | 0 | cp | BRD-K30563334 | rifabutin | Protein synthesis inhibitor |
| 2803 | 0 | cp | BRD-K08924299 | palonosetron | Serotonin receptor antagonist |
| 2804 | 0 | cp | BRD-A14208071 | oxyphenonium | Cholinergic receptor antagonist |
| 2805 | 0 | cp | BRD-K93618743 | ipriflavone | Bone resorption inhibitor |
| 2806 | 0 | cp | BRD-K59570838 | homoveratrylamine | Dopamine analog |
| 2807 | 0 | cp | BRD-K48932581 | cetraxate | Mucus protecting agent |
| 2808 | 0 | cp | BRD-K05658747 | raltegravir | HIV integrase inhibitor |
| 2809 | 0 | cp | BRD-K62982419 | cilomilast | Phosphodiesterase inhibitor |
| 2810 | 0 | cp | BRD-K11636097 | JNJ-7706621 | CDK inhibitor |
| 2811 | 0 | cp | BRD-K66175015 | afatinib | EGFR inhibitor |
| 2812 | 0 | cp | BRD-K29905972 | axitinib | PDGFR receptor inhibitor |
| 2813 | 0 | cp | BRD-A19248578 | latrunculin-b | Actin polymerization inhibitor |
| 2814 | 0 | cp | BRD-K13390322 | AT-7519 | CDK inhibitor |
| 2815 | 0 | cp | BRD-K79090631 | CGP-60474 | CDK inhibitor |
| 2816 | 0 | cp | BRD-K15402119 | huperzine-a | Acetylcholinesterase inhibitor |
| 2817 | 0 | cp | BRD-A47706533 | L-BSO | Glutathione transferase inhibitor |
| 2818 | 0 | cp | BRD-A61858259 | CAY-10415 | Insulin sensitizer |
| 2819 | 0 | cp | BRD-K89626439 | sirolimus | MTOR inhibitor |
| 2820 | 0 | cp | BRD-K09485525 | GANT-61 | GLI antagonist |
| 2821 | 0 | cp | BRD-K66896231 | BRD-K66896231 | Acetylcholinesterase inhibitor |
| 2822 | 0 | cp | BRD-K54987996 | CAY-10578 | Casein kinase inhibitor |
| 2823 | 0 | cp | BRD-A15010982 | HU-211 | Glutamate receptor antagonist |
| 2824 | 0 | cp | BRD-K78278890 | NM-PP1 | Mutant kinase inhibitor |
| 2825 | 0 | cp | BRD-A43331270 | niguldipine | Calcium channel blocker |
| 2826 | 0 | cp | BRD-K12502280 | TG-101348 | FLT3 inhibitor |
| 2827 | 0 | cp | BRD-A73680854 | PT-630 | Dipeptidyl peptidase inhibitor |
| 2828 | 0 | cp | BRD-K40892394 | AR-C133057XX | Nitric oxide synthase inhibitor |
| 2829 | 0 | cp | BRD-K74402642 | NSC-632839 | Ubiquitin specific protease inhibitor |
| 2830 | 0 | cp | BRD-K12516989 | zaprinast | Phosphodiesterase inhibitor |
| 2831 | 0 | cp | BRD-K53318339 | vinpocetine | Phosphodiesterase inhibitor |
| 2832 | 0 | cp | BRD-A55594068 | vinblastine | Microtubule inhibitor |
| 2833 | 0 | cp | BRD-A01295252 | trans-7-hydroxy-pipat | Dopamine receptor ligand |
| 2834 | 0 | cp | BRD-K45988865 | tetramethylsilane | Internal standard for NMR spectroscopy |
| 2835 | 0 | cp | BRD-K12867552 | THM-I-94 | HDAC inhibitor |
| 2836 | 0 | cp | BRD-K97330509 | SRC-kinase-inhibitor-II | SRC inhibitor |
| 2837 | 0 | cp | BRD-K22503835 | scriptaid | HDAC inhibitor |
| 2838 | 0 | cp | BRD-K96809896 | SKF-86002 | p38 MAPK inhibitor |
| 2839 | 0 | cp | BRD-K76805682 | SB-415286 | Glycogen synthase kinase inhibitor |
| 2840 | 0 | cp | BRD-K31627533 | rimexolone | Glucocorticoid receptor agonist |
| 2841 | 0 | cp | BRD-K82688027 | RG-13022 | PDGFR receptor inhibitor |
| 2842 | 0 | cp | BRD-K96862998 | pirfenidone | TGF beta receptor inhibitor |
| 2843 | 0 | cp | BRD-A37817666 | picrotoxin | GABA receptor antagonist |
| 2844 | 0 | cp | BRD-K95785537 | PP-2 | SRC inhibitor |
| 2845 | 0 | cp | BRD-A47633927 | NPC-15199 | ICAM1 antagonist |
| 2846 | 0 | cp | BRD-K78122587 | NNC-55-0396 | T-type calcium channel blocker |
| 2847 | 0 | cp | BRD-K60060639 | methyllidocaine | antiarrhythmic medication |
| 2848 | 0 | cp | BRD-K34441861 | moexipril | ACE inhibitor |
| 2849 | 0 | cp | BRD-K93201660 | ML-7 | Myosin light chain kinase inhibitor |
| 2850 | 0 | cp | BRD-K60230970 | MG-132 | Proteasome inhibitor |
| 2851 | 0 | cp | BRD-A41145729 | methoprene-acid | Retinoid receptor agonist |
| 2852 | 0 | cp | BRD-K96144918 | mead-acid | KPL-1 tumor suppressor |
| 2853 | 0 | cp | BRD-A85587465 | bemesetron | Serotonin receptor antagonist |
| 2854 | 0 | cp | BRD-K09635134 | l-erythro-MAPP | negative control for D-erythro-MAPP |
| 2855 | 0 | cp | BRD-K64044582 | linoleamide | ACAT inhibitor |
| 2856 | 0 | cp | BRD-K14618467 | IKK-16 | IKK inhibitor |
| 2857 | 0 | cp | BRD-A77722753 | hydralazine | Vasodilator |
| 2858 | 0 | cp | BRD-K07325606 | hispidin | PKC inhibitor |
| 2859 | 0 | cp | BRD-K81209159 | herniarin | Acetylcholinesterase inhibitor |
| 2860 | 0 | cp | BRD-K27737647 | H-89 | PKA inhibitor |
| 2861 | 0 | cp | BRD-K79930101 | GW-583340 | EGFR inhibitor |
| 2862 | 0 | cp | BRD-K16664969 | GTP-14564 | FLT3 inhibitor |
| 2863 | 0 | cp | BRD-K11911061 | GR-127935 | Serotonin receptor antagonist |
| 2864 | 0 | cp | BRD-K66944906 | fraxidin | Carbonic anhydrase inhibitor |
| 2865 | 0 | cp | BRD-K19360254 | ergocornine | Dopamine receptor agonist |
| 2866 | 0 | cp | BRD-K65814004 | diphenyleneiodonium | Nitric oxide synthase inhibitor |
| 2867 | 0 | cp | BRD-K18619710 | digoxigenin | Steroid |
| 2868 | 0 | cp | BRD-K33459542 | ditolylguanidine | Sigma receptor agonist |
| 2869 | 0 | cp | BRD-A73909368 | dactinomycin | RNA polymerase inhibitor |
| 2870 | 0 | cp | BRD-A10420615 | cyclopiazonic-acid | ATPase inhibitor |
| 2871 | 0 | cp | BRD-A82371568 | clofarabine | Ribonucleoside reductase inhibitor |
| 2872 | 0 | cp | BRD-A49358627 | ciprofibrate | PPAR receptor agonist |
| 2873 | 0 | cp | BRD-K65786282 | CGP-7930 | GABA receptor modulator |
| 2874 | 0 | cp | BRD-K31912990 | CGP-71683 | Neuropeptide receptor antagonist |
| 2875 | 0 | cp | BRD-K65503129 | HSP90-inhibitor | HSP inhibitor |
| 2876 | 0 | cp | BRD-K15616905 | CCCP | Mitochondrial oxidative phosphorylation uncoupler |
| 2877 | 0 | cp | BRD-A17428743 | BW-723C86 | Serotonin receptor agonist |
| 2878 | 0 | cp | BRD-K28075147 | biochanin-a | Estrogen receptor agonist |
| 2879 | 0 | cp | BRD-K91315211 | betahistine | Histamine receptor agonist |
| 2880 | 0 | cp | BRD-A84189516 | baccatin-III | Paclitaxel precursor |
| 2881 | 0 | cp | BRD-K33204703 | AG-370 | PDGFR receptor inhibitor |
| 2882 | 0 | cp | BRD-K14441456 | tyrphostin-AG-556 | EGFR inhibitor |
| 2883 | 0 | cp | BRD-K78294846 | osthol | Calcium channel blocker |
| 2884 | 0 | cp | BRD-A95445494 | maackiain | Sodium/glucose cotransporter inhibitor |
| 2885 | 0 | cp | BRD-K51018020 | VAMA-37 | DNA dependent protein kinase inhibitor |
| 2886 | 0 | cp | BRD-A52660433 | tetrindole | Monoamine oxidase inhibitor |
| 2887 | 0 | cp | BRD-K82562631 | tolmetin | Cyclooxygenase inhibitor |
| 2888 | 0 | cp | BRD-A42831637 | tetrahydrocannabinol-7-oic-acid | Anti-inflammatory |
| 2889 | 0 | cp | BRD-A22844106 | tenoxicam | Cyclooxygenase inhibitor |
| 2890 | 0 | cp | BRD-A72483914 | spiroxatrine | Serotonin receptor antagonist |
| 2891 | 0 | cp | BRD-A72066420 | mifobate | PPAR receptor antagonist |
| 2892 | 0 | cp | BRD-K51541829 | RO-25-6981 | Ionotropic glutamate receptor antagonist |
| 2893 | 0 | cp | BRD-A08003242 | rhodomyrtoxin-b | sodium fluorescein uptake inhibitor |
| 2894 | 0 | cp | BRD-K10098805 | rhapontin | Apoptosis stimulant |
| 2895 | 0 | cp | BRD-K49027941 | PSB-1115 | Adenosine receptor antagonist |
| 2896 | 0 | cp | BRD-A61470182 | n-formylmethionylalanine | macrophage activator |
| 2897 | 0 | cp | BRD-K35941380 | methysergide | Serotonin receptor antagonist |
| 2898 | 0 | cp | BRD-K36377456 | marmesin | Angiogenesis inhibitor |
| 2899 | 0 | cp | BRD-K47150025 | KI-8751 | VEGFR inhibitor |
| 2900 | 0 | cp | BRD-K49519092 | immethridine | Histamine receptor agonist |
| 2901 | 0 | cp | BRD-A48809242 | IB-MECA | Adenosine receptor agonist |
| 2902 | 0 | cp | BRD-A71157293 | fursultiamine | Vitamin B |
| 2903 | 0 | cp | BRD-A22143024 | estropipate | Estrogen receptor agonist |
| 2904 | 0 | cp | BRD-K17294426 | clebopride | Dopamine receptor antagonist |
| 4301 | 0 | cp | BRD-K49669041 | BX-912 | Pyruvate dehydrogenase kinase inhibitor |
| 4302 | 0 | cp | BRD-K87909389 | alvocidib | CDK inhibitor |
| 4303 | 0 | cp | BRD-K09991945 | GSK-3-inhibitor-II | PKC inhibitor |
| 4304 | 0 | cp | BRD-K77908580 | entinostat | HDAC inhibitor |
| 4305 | 0 | cp | BRD-K50417881 | eticlopride | Dopamine receptor antagonist |
| 4306 | 0 | cp | BRD-K97061094 | azacyclonol | Histamine receptor antagonist |
| 4307 | 0 | cp | BRD-K55127134 | fluphenazine | Dopamine receptor antagonist |
| 4308 | 0 | cp | BRD-K15262564 | mupirocin | Isoleucyl-tRNA synthetase inhibitor |
| 4309 | 0 | cp | BRD-A75368507 | demeclocycline | Bacterial 30S ribosomal subunit inhibitor |
| 4310 | 0 | cp | BRD-A24228527 | ofloxacin | Bacterial DNA gyrase inhibitor |
| 4311 | 0 | cp | BRD-A80638690 | floxuridine | DNA synthesis inhibitor |
| 4312 | 0 | cp | BRD-K02123250 | JNJ-38877605 | Tyrosine kinase inhibitor |
| 4313 | 0 | cp | BRD-A25067867 | benzatropine | Acetylcholine receptor antagonist |
| 4314 | 0 | cp | BRD-K52522949 | NCH-51 | HDAC inhibitor |
| 4315 | 0 | cp | BRD-K89348303 | ramipril | ACE inhibitor |
| 4316 | 0 | cp | BRD-K44497846 | enalapril | ACE inhibitor |
| 4317 | 0 | cp | BRD-K96037667 | norethindrone | Progesterone receptor agonist |
| 4318 | 0 | cp | BRD-A79237180 | ascorbic-acid | Antioxidant |
| 4319 | 0 | cp | BRD-A67605442 | tetrahydrobiopterin | Nitric oxide stimulant |
| 4320 | 0 | cp | BRD-K95237249 | probenecid | Uricosuric blocker |
| 4321 | 0 | cp | BRD-K10016611 | pyridine-2-aldoxime | Acetylcholinesterase inhibitor |
| 4322 | 0 | cp | BRD-M40783228 | mesna | Antioxidant |
| 4323 | 0 | cp | BRD-K62363391 | dapsone | Bacterial antifolate |
| 4324 | 0 | cp | BRD-K37194137 | III606050 | Cytochrome P450 inhibitor |
| 4325 | 0 | cp | BRD-K17823458 | danoprevir | HCV inhibitor |
| 4326 | 0 | cp | BRD-K71512533 | SNS-314 | Aurora kinase inhibitor |
| 4327 | 0 | cp | BRD-K17743125 | belinostat | HDAC inhibitor |
| 4328 | 0 | cp | BRD-A32161980 | carbetocin | Oxytocin receptor agonist |
| 4329 | 0 | cp | BRD-K07888107 | depudecin | HDAC inhibitor |
| 4330 | 0 | cp | BRD-A02333338 | cyclopamine | Smoothened receptor antagonist |
| 4331 | 0 | cp | BRD-K74501079 | azithromycin | Bacterial 50S ribosomal subunit inhibitor |
| 4332 | 0 | cp | BRD-K69328504 | L-690488 | Inositol monophosphatase inhibitor |
| 4333 | 0 | cp | BRD-K09499853 | KU-0060648 | DNA dependent protein kinase inhibitor |
| 4334 | 0 | cp | BRD-K91623615 | ABT-751 | Tubulin inhibitor |
| 4335 | 0 | cp | BRD-K81418486 | vorinostat | HDAC inhibitor |
| 4336 | 0 | cp | BRD-K74236984 | UNC-0321 | Histone lysine methyltransferase inhibitor |
| 4337 | 0 | cp | BRD-K55748775 | SCH-28080 | ATPase inhibitor |
| 4338 | 0 | cp | BRD-K26979635 | NS-3694 | Glutamate receptor antagonist |
| 4339 | 0 | cp | BRD-A63998256 | helveticoside | ATPase inhibitor |
| 4340 | 0 | cp | BRD-A73859745 | glycodeoxycholic-acid | Apoptosis stimulant |
| 4341 | 0 | cp | BRD-A13133631 | fluorometholone | Glucocorticoid receptor agonist |
| 4342 | 0 | cp | BRD-K61269089 | daphnetin | Protein kinase inhibitor |
| 2394 | 0.02 | cp | BRD-K04466929 | Merck60 | HDAC inhibitor |
| 2395 | 0.02 | cp | BRD-A91008255 | bepridil | Calcium channel blocker |
| 2396 | 0.02 | cp | BRD-K20755323 | SA-792728 | Sphingosine kinase inhibitor |
| 2390 | 0.03 | cp | BRD-K04710043 | hexamethylenebisacetamide | AKT inhibitor |
| 2391 | 0.03 | cp | BRD-K11717138 | benzbromarone | Chloride channel blocker |
| 2392 | 0.03 | cp | BRD-A22380646 | pantoprazole | ATPase inhibitor |
| 2393 | 0.03 | cp | BRD-K75958195 | pizotifen | Serotonin receptor antagonist |
| 2386 | 0.04 | cp | BRD-K63923597 | barasertib | Aurora kinase inhibitor |
| 2387 | 0.04 | cp | BRD-A36066264 | estradiol-benzoate | Estrogen receptor agonist |
| 2388 | 0.04 | cp | BRD-K02265150 | amoxapine | Norepinephrine reuptake inhibitor |
| 2377 | 0.07 | cp | BRD-K62374253 | rufloxacin | Bacterial DNA gyrase inhibitor |
| 2378 | 0.07 | cp | BRD-A49160188 | donepezil | Acetylcholinesterase inhibitor |
| 2379 | 0.07 | cp | BRD-A93424738 | dexamethasone | Glucocorticoid receptor agonist |
| 2380 | 0.07 | cp | BRD-K37991163 | paroxetine | Selective serotonin reuptake inhibitor (SSRI) |
| 2381 | 0.07 | cp | BRD-K35960502 | niclosamide | DNA replication inhibitor |
| 2382 | 0.07 | cp | BRD-K46211610 | tolazoline | Adrenergic receptor antagonist |
| 2383 | 0.07 | cp | BRD-A19736161 | ondansetron | Serotonin receptor antagonist |
| 2384 | 0.07 | cp | BRD-K38436528 | imipramine | Norepinephrine reuptake inhibitor |
| 2372 | 0.11 | cp | BRD-K08417745 | SID-26681509 | Cathepsin inhibitor |
| 2373 | 0.11 | cp | BRD-A40639672 | ketorolac | Cyclooxygenase inhibitor |
| 2374 | 0.11 | cp | BRD-K29313308 | HDAC3-selective | HDAC inhibitor |
| 2375 | 0.11 | cp | BRD-K01292756 | pimozide | Dopamine receptor antagonist |
| 2367 | 0.14 | cp | BRD-A07875874 | cilnidipine | Calcium channel blocker |
| 2368 | 0.14 | cp | BRD-K30240666 | clemastine | Histamine receptor antagonist |
| 2369 | 0.14 | cp | BRD-K92723993 | imatinib | BCR-ABL kinase inhibitor |
| 2364 | 0.16 | cp | BRD-K77695569 | tiabendazole | Angiogenesis inhibitor |
| 2357 | 0.18 | cp | BRD-A34255068 | rolipram | Phosphodiesterase inhibitor |
| 2358 | 0.18 | cp | BRD-A67862938 | naftidrofuryl | Adrenergic receptor antagonist |
| 2359 | 0.18 | cp | BRD-K42500029 | CGP-57380 | MAP kinase inhibitor |
| 2360 | 0.18 | cp | BRD-A80017228 | bendroflumethiazide | Sodium/potassium/chloride transporter inhibitor |
| 2361 | 0.18 | cp | BRD-K79425933 | benperidol | Dopamine receptor antagonist |
| 2362 | 0.18 | cp | BRD-A09533288 | verapamil | Calcium channel blocker |
| 2351 | 0.21 | cp | BRD-K88429204 | pyrimethamine | Dihydrofolate reductase inhibitor |
| 2352 | 0.21 | cp | BRD-K71499074 | diclofenamide | Carbonic anhydrase inhibitor |
| 2353 | 0.21 | cp | BRD-K96084870 | DMBI | PDGFR receptor inhibitor |
| 2354 | 0.21 | cp | BRD-K03670461 | tyrphostin-AG-82 | EGFR inhibitor |
| 2355 | 0.21 | cp | BRD-K71289571 | zafirlukast | Leukotriene receptor antagonist |
| 2356 | 0.21 | cp | BRD-K68132782 | terbinafine | Fungal squalene epoxidase inhibitor |
| 2350 | 0.23 | cp | BRD-K92000912 | AM-251 | Cannabinoid receptor antagonist |
| 2347 | 0.25 | cp | BRD-K14765469 | vesamicol | Acetylcholinesterase inhibitor |
| 2348 | 0.25 | cp | BRD-K12219985 | glipizide | Sulfonylurea |
| 2342 | 0.28 | cp | BRD-K21667562 | AM-404 | Cyclooxygenase inhibitor |
| 2344 | 0.28 | cp | BRD-A09722536 | cyclophosphamide | DNA alkylating agent |
| 2333 | 0.32 | cp | BRD-A81177136 | KN-62 | Calcium-calmodulin dependent protein kinase inhibitor |
| 2334 | 0.32 | cp | BRD-K88759641 | EMD-66684 | Angiotensin receptor antagonist |
| 2335 | 0.32 | cp | BRD-A58564983 | selamectin | Nematocide |
| 2336 | 0.32 | cp | BRD-K46317332 | proadifen | Nitric oxide synthase inhibitor |
| 2337 | 0.32 | cp | BRD-K76810206 | nicergoline | Adrenergic receptor antagonist |
| 2340 | 0.32 | cp | BRD-A08187463 | racecadotril | Enkephalinase inhibitor |
| 2341 | 0.32 | cp | BRD-A26384407 | chlortalidone | Carbonic anhydrase inhibitor |
| 2328 | 0.35 | cp | BRD-K90524085 | MY-5445 | Phosphodiesterase inhibitor |
| 2329 | 0.35 | cp | BRD-K59256312 | gabexate | Serine protease inhibitor |
| 2330 | 0.35 | cp | BRD-K64245000 | GW-4064 | FXR agonist |
| 2324 | 0.39 | cp | BRD-K32526544 | DCEBIO | Potassium channel activator |
| 2325 | 0.39 | cp | BRD-K09132007 | D-4476 | TGF beta receptor inhibitor |
| 2326 | 0.39 | cp | BRD-K28360340 | TW-37 | BCL inhibitor |
| 2318 | 0.42 | cp | BRD-K45158365 | valsartan | Angiotensin receptor antagonist |
| 2319 | 0.42 | cp | BRD-K37865504 | LY-2183240 | FAAH inhibitor |
| 2320 | 0.42 | cp | BRD-K53414658 | tivozanib | VEGFR inhibitor |
| 2309 | 0.46 | cp | BRD-K13049116 | BMS-754807 | IGF-1 inhibitor |
| 2310 | 0.46 | cp | BRD-K59962020 | CHEMBL-374350 | NFkB pathway inhibitor |
| 2311 | 0.46 | cp | BRD-A70649075 | sulconazole | Sterol demethylase inhibitor |
| 2312 | 0.46 | cp | BRD-K15715913 | fluperlapine | Serotonin receptor antagonist |
| 2313 | 0.46 | cp | BRD-A69917777 | aminopentamide | Acetylcholine receptor antagonist |
| 2306 | 0.49 | cp | BRD-K52080565 | rilmenidine | Imidazoline receptor agonist |
| 2307 | 0.49 | cp | BRD-K55454768 | TAS-301 | Calcium-calmodulin dependent protein kinase inhibitor |
| 2297 | 0.53 | cp | BRD-K28296557 | AKT-inhibitor-IV | AKT inhibitor |
| 2298 | 0.53 | cp | BRD-K13926615 | vardenafil | Phosphodiesterase inhibitor |
| 2299 | 0.53 | cp | BRD-K88741031 | methyl-2,5-dihydroxycinnamate | EGFR inhibitor |
| 2301 | 0.53 | cp | BRD-A70407468 | PSB-36 | Adenosine receptor antagonist |
| 2295 | 0.54 | cp | BRD-A17535965 | gelsemine | Acetylcholine receptor antagonist |
| 2291 | 0.56 | cp | BRD-A49172652 | lansoprazole | ATPase inhibitor |
| 2292 | 0.56 | cp | BRD-K00959089 | thenoyltrifluoroacetone | Chelating agent |
| 2289 | 0.57 | cp | BRD-K15935639 | z-leu3-VS | Proteasome inhibitor |
| 2283 | 0.6 | cp | BRD-K47936004 | piribedil | Dopamine receptor agonist |
| 2284 | 0.6 | cp | BRD-K60274257 | dephostatin | Tyrosine phosphatase inhibitor |
| 2287 | 0.6 | cp | BRD-K63675182 | triflupromazine | Dopamine receptor antagonist |
| 2277 | 0.63 | cp | BRD-K67831364 | ZM-323881 | VEGFR inhibitor |
| 2278 | 0.63 | cp | BRD-A15131297 | benazepril | ACE inhibitor |
| 2281 | 0.63 | cp | BRD-K97810537 | beclometasone | Glucocorticoid receptor agonist |
| 2274 | 0.65 | cp | BRD-A97437073 | rosiglitazone | Insulin sensitizer |
| 2269 | 0.66 | cp | BRD-K74765201 | tomelukast | Leukotriene receptor antagonist |
| 2259 | 0.7 | cp | BRD-K71035033 | masitinib | KIT inhibitor |
| 2260 | 0.7 | cp | BRD-A31801025 | formestane | Aromatase inhibitor |
| 2261 | 0.7 | cp | BRD-K37206356 | rhamnetin | HDAC inhibitor |
| 2262 | 0.7 | cp | BRD-A36267905 | buphenine | Adrenergic receptor agonist |
| 2263 | 0.7 | cp | BRD-A21858158 | praziquantel | Anthelmintic |
| 2251 | 0.74 | cp | BRD-A41692738 | TGX-221 | PI3K inhibitor |
| 2255 | 0.74 | cp | BRD-K21450440 | benzthiazide | Carbonic anhydrase inhibitor |
| 2256 | 0.74 | cp | BRD-A93255169 | thalidomide | TNF production inhibitor |
| 2245 | 0.77 | cp | BRD-K78633253 | EXO-1 | ARF inhibitor |
| 2241 | 0.78 | cp | BRD-A98283014 | calmidazolium | Calcium channel blocker |
| 2242 | 0.78 | cp | BRD-K50168500 | canertinib | EGFR inhibitor |
| 2243 | 0.78 | cp | BRD-K92138166 | mammea-a | other antibiotic |
| 2244 | 0.78 | cp | BRD-K89997465 | chlorpromazine | Dopamine receptor antagonist |
| 2232 | 0.81 | cp | BRD-K89125793 | tinidazole | Antiprotozoal |
| 2233 | 0.81 | cp | BRD-K39621635 | artemether | Antimalarial |
| 2234 | 0.81 | cp | BRD-K51302260 | KU-C103871 | GSP agonist |
| 2235 | 0.81 | cp | BRD-K68392338 | ZK-93426 | Benzodiazepine receptor antagonist |
| 2236 | 0.81 | cp | BRD-K67847053 | guanabenz | Adrenergic receptor agonist |
| 2238 | 0.81 | cp | BRD-A70083328 | secnidazole | Acetylcholinesterase inhibitor |
| 2225 | 0.85 | cp | BRD-K36627727 | tamibarotene | Retinoid receptor agonist |
| 2226 | 0.85 | cp | BRD-K46435977 | valaciclovir | DNA polymerase inhibitor |
| 2227 | 0.85 | cp | BRD-K84566043 | fenpiverinium | Acetylcholine receptor antagonist |
| 2228 | 0.85 | cp | BRD-K64402243 | ivachtin | Caspase inhibitor |
| 2229 | 0.85 | cp | BRD-A93236127 | digitoxin | ATPase inhibitor |
| 2223 | 0.87 | cp | BRD-K34776109 | glimepiride | Insulin secretagogue |
| 2220 | 0.88 | cp | BRD-K91263825 | nortriptyline | Tricyclic antidepressant |
| 2221 | 0.88 | cp | BRD-K16478699 | PLX-4720 | RAF inhibitor |
| 2216 | 0.92 | cp | BRD-K29530284 | amlexanox | Histamine receptor modulator |
| 2217 | 0.92 | cp | BRD-K99696746 | fatostatin | SREBP inhibitor |
| 2218 | 0.92 | cp | BRD-K75699339 | rizatriptan | Serotonin receptor agonist |
| 2214 | 0.93 | cp | BRD-K10065684 | dantron | Laxative |
| 2208 | 0.95 | cp | BRD-K92870997 | pterostilbene | Cyclooxygenase inhibitor |
| 2211 | 0.95 | cp | BRD-A65280694 | molindone | Dopamine receptor antagonist |
| 2198 | 1 | cp | BRD-K78959463 | FPL-64176 | Calcium channel activator |
| 2192 | 1.02 | cp | BRD-K60460488 | nelfinavir | HIV protease inhibitor |
| 2193 | 1.02 | cp | BRD-K95435023 | PHA-665752 | c-Met inhibitor |
| 2194 | 1.02 | cp | BRD-K23583188 | lavendustin-a | EGFR inhibitor |
| 2195 | 1.02 | cp | BRD-K17008822 | BD-1008 | Sigma receptor antagonist |
| 2184 | 1.06 | cp | BRD-K86930074 | cediranib | KIT inhibitor |
| 2185 | 1.06 | cp | BRD-A65550283 | ginsenoside | Steroid hormone receptor agonist |
| 2186 | 1.06 | cp | BRD-K72895815 | SSR-69071 | Leukocyte elastase inhibitor |
| 2182 | 1.07 | cp | BRD-K09471561 | levofloxacin | Bacterial DNA gyrase inhibitor |
| 2179 | 1.09 | cp | BRD-K72420232 | WZ-4002 | EGFR inhibitor |
| 2169 | 1.13 | cp | BRD-A20968261 | WAY-213613 | Glutamate inhibitor |
| 2170 | 1.13 | cp | BRD-K35430135 | SR-59230A | Adrenergic receptor antagonist |
| 2171 | 1.13 | cp | BRD-A43882281 | pinacidil | ATP channel activator |
| 2172 | 1.13 | cp | BRD-K93332168 | isocarboxazid | Monoamine oxidase inhibitor |
| 2173 | 1.13 | cp | BRD-K37080523 | isoreserpine | Vesicular monoamine transporter inhibitor |
| 2168 | 1.14 | cp | BRD-K02590140 | O-2050 | Cannabinoid receptor antagonist |
| 2161 | 1.16 | cp | BRD-A25569250 | KI-16425 | Lysophosphatidic acid receptor antagonist |
| 2163 | 1.16 | cp | BRD-K47869605 | podophyllotoxin | Microtubule inhibitor |
| 2164 | 1.16 | cp | BRD-K63828191 | raloxifene | Estrogen receptor antagonist |
| 2157 | 1.18 | cp | BRD-K16485616 | mocetinostat | HDAC inhibitor |
| 2156 | 1.19 | cp | BRD-K13642330 | cosmosiin | Cytochrome P450 inhibitor |
| 2152 | 1.2 | cp | BRD-K96740444 | itopride | Dopamine receptor antagonist |
| 2153 | 1.2 | cp | BRD-K07220430 | cinnarizine | Calcium channel blocker |
| 2145 | 1.23 | cp | BRD-K22385716 | LY-303511 | Casein kinase inhibitor |
| 2146 | 1.23 | cp | BRD-A26095496 | clobetasol | Glucocorticoid receptor agonist |
| 2147 | 1.23 | cp | BRD-K08806317 | timolol | Adrenergic receptor antagonist |
| 2148 | 1.23 | cp | BRD-K59184148 | SB-216763 | Glycogen synthase kinase inhibitor |
| 2134 | 1.27 | cp | BRD-K44779798 | miglitol | Glucosidase inhibitor |
| 2135 | 1.27 | cp | BRD-A48720949 | testosterone | androgen receptor agonist |
| 2136 | 1.27 | cp | BRD-A49046702 | SKF-89976A | GABA uptake inhibitor |
| 2137 | 1.27 | cp | BRD-A17453586 | MDL-72832 | Serotonin receptor agonist |
| 2138 | 1.27 | cp | BRD-K73391359 | quinisocaine | Local anesthetic |
| 2139 | 1.27 | cp | BRD-K98490050 | amsacrine | Topoisomerase inhibitor |
| 2140 | 1.27 | cp | BRD-K25311561 | KU-55933 | ATM kinase inhibitor |
| 2141 | 1.27 | cp | BRD-A58048407 | nimodipine | Calcium channel blocker |
| 2133 | 1.28 | cp | BRD-K08619838 | tremorine | Acetylcholine receptor agonist |
| 2120 | 1.33 | cp | BRD-K92413528 | thiazolidinecarboxylic-acid | Reducing agent |
| 2121 | 1.33 | cp | BRD-K88742110 | BRD-K88742110 | HDAC inhibitor |
| 2118 | 1.34 | cp | BRD-K82357231 | desloratadine | Histamine receptor antagonist |
| 2119 | 1.34 | cp | BRD-K40578143 | GR-79236 | Adenosine receptor agonist |
| 2116 | 1.37 | cp | BRD-A36318220 | necrostatin-1 | RIPK inhibitor |
| 2117 | 1.37 | cp | BRD-K81709173 | halcinonide | Glucocorticoid receptor agonist |
| 2110 | 1.39 | cp | BRD-K06467078 | corynanthine | Adrenergic receptor antagonist |
| 2101 | 1.41 | cp | BRD-K15916496 | clotrimazole | Cytochrome P450 inhibitor |
| 2102 | 1.41 | cp | BRD-A06390036 | hydroquinidine | Antiarrhythmic |
| 2103 | 1.41 | cp | BRD-K97181089 | amiloride | Sodium channel blocker |
| 2099 | 1.42 | cp | BRD-K16444452 | ibudilast | Leukotriene receptor antagonist |
| 2094 | 1.44 | cp | BRD-K95885906 | quercetagetin | PIM inhibitor |
| 2095 | 1.44 | cp | BRD-K86727142 | embelin | HCV inhibitor |
| 2096 | 1.44 | cp | BRD-K62353524 | DY-131 | Estrogen receptor agonist |
| 2080 | 1.48 | cp | BRD-K95901403 | XL-147 | PI3K inhibitor |
| 2081 | 1.48 | cp | BRD-K15588452 | R-96544 | Serotonin receptor antagonist |
| 2082 | 1.48 | cp | BRD-K49671696 | ketanserin | Serotonin receptor antagonist |
| 2083 | 1.48 | cp | BRD-A02759312 | betaxolol | Adrenergic receptor antagonist |
| 2084 | 1.48 | cp | BRD-K63792901 | arecaidine | Acetylcholine receptor agonist |
| 2088 | 1.48 | cp | BRD-K65417056 | meprylcaine | Local anesthetic |
| 2089 | 1.48 | cp | BRD-A09472452 | flecainide | Sodium channel blocker |
| 2066 | 1.52 | cp | BRD-A84327315 | calcitriol | Vitamin D receptor agonist |
| 2067 | 1.52 | cp | BRD-K04833372 | GSK-1904529A | IGF-1 inhibitor |
| 2068 | 1.52 | cp | BRD-K38449220 | seneciphylline | Cytochrome P450 inhibitor |
| 2069 | 1.52 | cp | BRD-K04111260 | raclopride | Dopamine receptor antagonist |
| 2070 | 1.52 | cp | BRD-K17868609 | BRL-54443 | Serotonin receptor agonist |
| 2056 | 1.59 | cp | BRD-K91243525 | SR-142948 | Neurotensin receptor antagonist |
| 2057 | 1.59 | cp | BRD-A89337244 | PD-102807 | Acetylcholine receptor antagonist |
| 2058 | 1.59 | cp | BRD-K66782112 | BRD-K66782112 | Histamine receptor antagonist |
| 2049 | 1.62 | cp | BRD-A24191444 | ifenprodil | Adrenergic receptor antagonist |
| 2043 | 1.66 | cp | BRD-K70330367 | amantadine | Glutamate receptor antagonist |
| 2044 | 1.66 | cp | BRD-K74133369 | oligomycin-a | ATP synthase inhibitor |
| 2034 | 1.68 | cp | BRD-A27554692 | altrenogest | Progestogen hormone |
| 2033 | 1.69 | cp | BRD-K61323504 | SB-225002 | CC chemokine receptor antagonist |
| 2027 | 1.72 | cp | BRD-K29668683 | BD-1063 | Sigma receptor antagonist |
| 2025 | 1.73 | cp | BRD-K44094599 | tacrolimus | Calcineurin inhibitor |
| 2026 | 1.73 | cp | BRD-K08252256 | diclofenac | Cyclooxygenase inhibitor |
| 2017 | 1.76 | cp | BRD-K28137194 | loreclezole | GABA receptor agonist |
| 2018 | 1.76 | cp | BRD-K82983861 | GW-0742 | PPAR receptor agonist |
| 2011 | 1.8 | cp | BRD-K67860401 | AR-A014418 | Glycogen synthase kinase inhibitor |
| 2010 | 1.81 | cp | BRD-K05528470 | L-745870 | Dopamine receptor antagonist |
| 2006 | 1.83 | cp | BRD-K05151076 | ZK-164015 | Estrogen receptor antagonist |
| 2007 | 1.83 | cp | BRD-K35559145 | levomepromazine | Dopamine receptor antagonist |
| 2008 | 1.83 | cp | BRD-A30205217 | ethotoin | Hydantoin antiepileptic |
| 2002 | 1.86 | cp | BRD-A89434049 | sarmentogenin | ATPase inhibitor |
| 1996 | 1.87 | cp | BRD-K32744045 | disulfiram | Aldehyde dehydrogenase inhibitor |
| 1997 | 1.87 | cp | BRD-K10670311 | sulfasalazine | Antirheumatic |
| 1998 | 1.87 | cp | BRD-K56047318 | RHC-80267 | Triacylglycerol lipase inhibitor |
| 1999 | 1.87 | cp | BRD-K82561139 | ricinine | Casein kinase inhibitor |
| 2000 | 1.87 | cp | BRD-K36616567 | doxepin | Histamine receptor antagonist |
| 2001 | 1.87 | cp | BRD-A37837077 | cyclazosin | Adrenergic receptor antagonist |
| 1995 | 1.88 | cp | BRD-A01826957 | xanthinol | Vasodilator |
| 1994 | 1.89 | cp | BRD-A95869247 | indapamide | Thiazide diuretic |
| 1988 | 1.9 | cp | BRD-A49765801 | fludroxycortide | Glucocorticoid receptor agonist |
| 1989 | 1.9 | cp | BRD-K31054881 | BMY-7378 | Adrenergic receptor antagonist |
| 1990 | 1.9 | cp | BRD-A42759514 | ornidazole | Antiprotozoal |
| 1979 | 1.94 | cp | BRD-K71430621 | clobenpropit | Histamine receptor antagonist |
| 1980 | 1.94 | cp | BRD-A16665823 | butoconazole | Bacterial cell wall synthesis inhibitor |
| 1973 | 1.97 | cp | BRD-K57930253 | nitrazepam | Benzodiazepine receptor agonist |
| 1974 | 1.97 | cp | BRD-K01638814 | rilmenidine | Adrenergic receptor agonist |
| 1975 | 1.97 | cp | BRD-A56012032 | thiorphan | Membrane metalloendopeptidase inhibitor |
| 1976 | 1.97 | cp | BRD-K65146499 | nabumetone | Cyclooxygenase inhibitor |
| 1964 | 2 | cp | BRD-K74514084 | pazopanib | KIT inhibitor |
| 1960 | 2.01 | cp | BRD-A20697603 | thiostrepton | FOXM1 inhibitor |
| 1961 | 2.01 | cp | BRD-K51918615 | iodophenpropit | Histamine receptor antagonist |
| 1963 | 2.01 | cp | BRD-K85133207 | HDAC1-selective | HDAC inhibitor |
| 1955 | 2.04 | cp | BRD-K35629949 | SR-27897 | CCK receptor antagonist |
| 1943 | 2.08 | cp | BRD-K59469039 | AG-879 | Angiogenesis inhibitor |
| 1941 | 2.09 | cp | BRD-K50866992 | tropisetron | Serotonin receptor antagonist |
| 1940 | 2.1 | cp | BRD-A90131694 | alclometasone | Glucocorticoid receptor agonist |
| 1938 | 2.11 | cp | BRD-K74913225 | brinzolamide | Carbonic anhydrase inhibitor |
| 1925 | 2.15 | cp | BRD-K79684402 | RO-10-5824 | Dopamine receptor agonist |
| 1926 | 2.15 | cp | BRD-K09549677 | mibefradil | T-type calcium channel blocker |
| 1928 | 2.15 | cp | BRD-K67261995 | adipiodone | Contrast agent |
| 1924 | 2.16 | cp | BRD-A13084692 | troglitazone | Insulin sensitizer |
| 1921 | 2.18 | cp | BRD-K14681867 | somatostatin | Somatostatin receptor agonist |
| 1923 | 2.18 | cp | BRD-K66876909 | linezolid | Bacterial 50S ribosomal subunit inhibitor |
| 1917 | 2.19 | cp | BRD-K11399644 | phenformin | AMPK activator |
| 1915 | 2.2 | cp | BRD-A92161634 | scopoline | Acetylcholine receptor antagonist |
| 1914 | 2.21 | cp | BRD-K41895714 | AS-605240 | PI3K inhibitor |
| 1899 | 2.26 | cp | BRD-K09397065 | SR-57227A | Serotonin receptor agonist |
| 1900 | 2.26 | cp | BRD-A32164164 | methyllycaconitine | Acetylcholine receptor antagonist |
| 1901 | 2.26 | cp | BRD-A30437061 | camptothecin | Topoisomerase inhibitor |
| 1904 | 2.26 | cp | BRD-K15592317 | CP466722 | ATM kinase inhibitor |
| 1893 | 2.29 | cp | BRD-K37312348 | kenpaullone | CDK inhibitor |
| 1885 | 2.33 | cp | BRD-K88358234 | xaliproden | Serotonin receptor agonist |
| 1882 | 2.34 | cp | BRD-K53263234 | CITCO | CAR agonist |
| 1880 | 2.36 | cp | BRD-K36258877 | AZ-10417808 | Caspase inhibitor |
| 1874 | 2.4 | cp | BRD-K62289640 | lylamine | Cannabinoid receptor agonist |
| 1875 | 2.4 | cp | BRD-K51318897 | fenbendazole | Tubulin inhibitor |
| 1872 | 2.41 | cp | BRD-K15025317 | BAY-11-7821 | NFkB pathway inhibitor |
| 1865 | 2.42 | cp | BRD-K70401845 | erlotinib | EGFR inhibitor |
| 1858 | 2.47 | cp | BRD-K34415467 | trimethobenzamide | Histamine receptor antagonist |
| 1857 | 2.48 | cp | BRD-K48722833 | iloperidone | Dopamine receptor antagonist |
| 1849 | 2.5 | cp | BRD-A83892713 | rifampicin | RNA polymerase inhibitor |
| 1850 | 2.5 | cp | BRD-K54095730 | CMPD-1 | p38 MAPK inhibitor |
| 1854 | 2.5 | cp | BRD-K07237224 | moclobemide | Monoamine oxidase inhibitor |
| 1855 | 2.5 | cp | BRD-K77175907 | calcifediol | Vitamin D receptor agonist |
| 1844 | 2.54 | cp | BRD-K79116891 | proxymetacaine | Sodium channel blocker |
| 1836 | 2.56 | cp | BRD-A27143604 | DPN | Estrogen receptor agonist |
| 1834 | 2.57 | cp | BRD-K07762753 | aminopurvalanol-a | Tyrosine kinase inhibitor |
| 1835 | 2.57 | cp | BRD-K76205745 | losartan | Angiotensin receptor antagonist |
| 1832 | 2.58 | cp | BRD-K45542189 | diethylcarbamazine | Lipoxygenase inhibitor |
| 1827 | 2.6 | cp | BRD-K84709232 | caffeic-acid | Lipoxygenase inhibitor |
| 1818 | 2.64 | cp | BRD-A38913120 | BH3I-1 | BCL inhibitor |
| 1820 | 2.64 | cp | BRD-K63630713 | etacrynic-acid | Sodium/potassium/chloride transporter inhibitor |
| 1811 | 2.68 | cp | BRD-K41170226 | deoxycholic-acid | G protein-coupled receptor agonist |
| 1812 | 2.68 | cp | BRD-A33833419 | TER-14687 | Inhibitor of translocation of PKCq in T cells |
| 1813 | 2.68 | cp | BRD-K97564742 | mepyramine | Histamine receptor antagonist |
| 1806 | 2.72 | cp | BRD-A87606379 | nadolol | Adrenergic receptor antagonist |
| 1803 | 2.75 | cp | BRD-K52620403 | STO-609 | Calmodulin antagonist |
| 1804 | 2.75 | cp | BRD-A44008656 | doxylamine | Histamine receptor antagonist |
| 1795 | 2.78 | cp | BRD-A27489425 | rolitetracycline | Bacterial 30S ribosomal subunit inhibitor |
| 1796 | 2.78 | cp | BRD-K76587808 | fraxetin | Antioxidant |
| 1786 | 2.82 | cp | BRD-A03816571 | CP-55940 | Cannabinoid receptor agonist |
| 1785 | 2.84 | cp | BRD-K07403598 | CAY-10470 | NFkB pathway inhibitor |
| 1777 | 2.89 | cp | BRD-K15409150 | penfluridol | T-type calcium channel blocker |
| 1780 | 2.89 | cp | BRD-K81272440 | dantrolene | Calcium channel blocker |
| 1776 | 2.91 | cp | BRD-K74305673 | IKK-2-inhibitor-V | IKK inhibitor |
| 1773 | 2.92 | cp | BRD-A68930007 | ouabain | ATPase inhibitor |
| 1775 | 2.92 | cp | BRD-A34208323 | VU-0404997-2 | Glutamate receptor modulator |
| 1766 | 2.96 | cp | BRD-K06221026 | DUP-697 | Cyclooxygenase inhibitor |
| 1770 | 2.96 | cp | BRD-K25504083 | cytochalasin-d | Actin polymerization inhibitor |
| 1765 | 2.97 | cp | BRD-K02637541 | celecoxib | Cyclooxygenase inhibitor |
| 1761 | 3.01 | cp | BRD-A38030642 | cyclosporin-a | Calcineurin inhibitor |
| 1759 | 3.03 | cp | BRD-A06352418 | terfenadine | Histamine receptor antagonist |
| 1760 | 3.03 | cp | BRD-K35498378 | alrestatin | Aldose reductase inhibitor |
| 1758 | 3.04 | cp | BRD-K52394958 | GR-159897 | Tachykinin antagonist |
| 1754 | 3.05 | cp | BRD-K76534306 | enrofloxacin | Bacterial DNA gyrase inhibitor |
| 1757 | 3.05 | cp | BRD-A37492983 | iocetamic-acid | Radiopaque medium |
| 1750 | 3.07 | cp | BRD-K53545112 | CNQX | Glutamate receptor antagonist |
| 1751 | 3.07 | cp | BRD-K41410256 | balsalazide | Cyclooxygenase inhibitor |
| 1749 | 3.08 | cp | BRD-A37347161 | BRL-52537 | Opioid receptor agonist |
| 1747 | 3.1 | cp | BRD-K03384561 | roquinimex | Angiogenesis inhibitor |
| 1739 | 3.12 | cp | BRD-K81473089 | tacrine | Acetylcholinesterase inhibitor |
| 1734 | 3.17 | cp | BRD-K82731415 | olomoucine | CDK inhibitor |
| 1731 | 3.2 | cp | BRD-A63546914 | RO-04-5595 | Glutamate receptor antagonist |
| 1726 | 3.21 | cp | BRD-A58157837 | butabindide | Tripeptidyl peptidase inhibitor |
| 1727 | 3.21 | cp | BRD-K54210043 | NS-1619 | Calcium channel activator |
| 1730 | 3.21 | cp | BRD-K71799949 | carbamazepine | Carboxamide antiepileptic |
| 1718 | 3.24 | cp | BRD-K59037100 | oxybenzone | Lipase inhibitor |
| 1719 | 3.24 | cp | BRD-K06024458 | n-arachidonyl-GABA | cannabinoid receptor agonist |
| 1724 | 3.24 | cp | BRD-K07303502 | arachidonyl-trifluoro-methane | Cytosolic phospholipase inhibitor |
| 1716 | 3.26 | cp | BRD-K11905747 | spectinomycin | Bacterial 30S ribosomal subunit inhibitor |
| 1717 | 3.26 | cp | BRD-K21548250 | moracizine | Sodium channel blocker |
| 1706 | 3.31 | cp | BRD-K67680372 | CI-966 | GAT inhibitor |
| 1704 | 3.32 | cp | BRD-A87719232 | naproxen | Cyclooxygenase inhibitor |
| 1697 | 3.38 | cp | BRD-K59753853 | MDL-29951 | Glutamate receptor antagonist |
| 1691 | 3.42 | cp | BRD-K20313525 | rosmarinic-acid | GABA transaminase inhibitor |
| 1692 | 3.42 | cp | BRD-K63089472 | farnesylthioacetic-acid | Inhibitor of methyl esterification of farnesylated proteins |
| 1693 | 3.42 | cp | BRD-K37447567 | hydrocotarnine | Opioid receptor antagonist |
| 1688 | 3.45 | cp | BRD-K78431006 | crizotinib | ALK inhibitor |
| 1687 | 3.46 | cp | BRD-K32836707 | CAY-10577 | Casein kinase inhibitor |
| 1686 | 3.47 | cp | BRD-A35912562 | pregnenolone | Glutamate receptor modulator |
| 1681 | 3.52 | cp | BRD-A53131506 | epitestosterone | Inactive testosterone analog |
| 1678 | 3.55 | cp | BRD-A45333398 | periplocymarin | Apoptosis stimulant |
| 1676 | 3.56 | cp | BRD-K23369905 | oxiconazole | Bacterial cell wall synthesis inhibitor |
| 1674 | 3.59 | cp | BRD-A75935363 | atracurium | Acetylcholine receptor antagonist |
| 1671 | 3.63 | cp | BRD-K43149758 | myricetin | Androgen receptor agonist |
| 1666 | 3.7 | cp | BRD-A78877355 | nefopam | Cyclooxygenase inhibitor |
| 1667 | 3.7 | cp | BRD-A45889380 | mepacrine | Cytokine production inhibitor |
| 1662 | 3.74 | cp | BRD-K54771420 | glycocholic-acid | Cholesterol inhibitor |
| 1660 | 3.77 | cp | BRD-K75181824 | acetyl-geranygeranyl-cysteine | Inhibitor of methyl esterification of geranylgeranylated proteins |
| 1656 | 3.81 | cp | BRD-K94832621 | Y-134 | Estrogen receptor antagonist |
| 1654 | 3.84 | cp | BRD-K22631935 | neurodazine | Neurogenesis of non-pluripotent C2C12 myoblast inducer |
| 1655 | 3.84 | cp | BRD-K68190965 | GR-46611 | Serotonin receptor agonist |
| 1652 | 3.86 | cp | BRD-K16508793 | diazepam | Benzodiazepine receptor agonist |
| 1649 | 3.88 | cp | BRD-K83322645 | L-693403 | Sigma receptor agonist |
| 1646 | 3.92 | cp | BRD-K52989797 | clomipramine | Serotonin transporter inhibitor (SERT) |
| 1641 | 3.95 | cp | BRD-K57033106 | tripelennamine | Histamine receptor antagonist |
| 1642 | 3.95 | cp | BRD-A02713983 | dihydrodeoxygedunin | Growth factor receptor activator |
| 1640 | 3.97 | cp | BRD-K06593056 | LE-135 | Retinoid receptor agonist |
| 1637 | 3.98 | cp | BRD-K25079130 | avrainvillamide-analog-4 | nucleophosmin inhibitor |
| 1638 | 3.98 | cp | BRD-A71459254 | cymarin | ATPase inhibitor |
| 1634 | 4.02 | cp | BRD-A53576514 | orphenadrine | Acetylcholine receptor antagonist |
| 1629 | 4.05 | cp | BRD-K61097567 | SB-218795 | Tachykinin antagonist |
| 1630 | 4.05 | cp | BRD-K18059238 | gamma-linolenic-acid | Cyclooxygenase inhibitor |
| 1620 | 4.09 | cp | BRD-K26863634 | BIX-01338 | Histone lysine methyltransferase inhibitor |
| 1621 | 4.09 | cp | BRD-A09094913 | strychnine | Acetylcholine receptor antagonist |
| 1619 | 4.11 | cp | BRD-K84595254 | strophanthidin | ATPase inhibitor |
| 1616 | 4.12 | cp | BRD-A71009679 | KUC103420N | -666 |
| 1617 | 4.12 | cp | BRD-K94830329 | ataluren | CFTR channel agonist |
| 1610 | 4.19 | cp | BRD-A06352508 | SB-218078 | CHK inhibitor |
| 1605 | 4.23 | cp | BRD-K61217870 | n-(3-acetamidophenyl)-3-chlorobenzamide | Glutamate receptor antagonist |
| 1604 | 4.26 | cp | BRD-A80793822 | pemoline | Dopamine receptor agonist |
| 1599 | 4.3 | cp | BRD-K78485176 | olmesartan | Angiotensin receptor antagonist |
| 1589 | 4.38 | cp | BRD-A07440155 | labetalol | Adrenergic receptor antagonist |
| 1579 | 4.44 | cp | BRD-K08554278 | bisbenzimide | DNA binding agent |
| 1576 | 4.45 | cp | BRD-K67537649 | PQ-401 | IGF-1 inhibitor |
| 1574 | 4.47 | cp | BRD-K09907482 | PRL-3-inhibitor-I | Tyrosine phosphatase inhibitor |
| 1571 | 4.51 | cp | BRD-K02965346 | SU-11274 | Hepatocyte growth factor receptor inhibitor |
| 1568 | 4.55 | cp | BRD-K93188295 | ARC-239 | Adrenergic receptor antagonist |
| 1565 | 4.58 | cp | BRD-K52850071 | JAK3-Inhibitor-II | JAK inhibitor |
| 1561 | 4.62 | cp | BRD-K57926513 | tyrphostin-AG-1295 | PDGFR receptor inhibitor |
| 1562 | 4.62 | cp | BRD-K45296539 | ZD-7114 | Adrenergic receptor agonist |
| 1554 | 4.69 | cp | BRD-K22662435 | ganciclovir | DNA polymerase inhibitor |
| 1549 | 4.75 | cp | BRD-K89055274 | alverine | Muscle relaxant |
| 1545 | 4.76 | cp | BRD-K00673382 | famotidine | Histamine receptor antagonist |
| 1548 | 4.76 | cp | BRD-K93280214 | gabazine | GABA receptor antagonist |
| 1541 | 4.81 | cp | BRD-K78599730 | manumycin-a | Farnesyltransferase inhibitor |
| 1537 | 4.84 | cp | BRD-A11990600 | lorazepam | Benzodiazepine receptor agonist |
| 1539 | 4.84 | cp | BRD-K98548675 | parthenolide | NFkB pathway inhibitor |
| 1534 | 4.86 | cp | BRD-K05104363 | PD-184352 | MEK inhibitor |
| 1535 | 4.86 | cp | BRD-K85383046 | IAA-94 | Chloride channel blocker |
| 1531 | 4.87 | cp | BRD-K08996725 | zolantidine | Histamine receptor antagonist |
| 1530 | 4.9 | cp | BRD-K48029790 | OBAA | Phospholipase inhibitor |
| 1525 | 4.97 | cp | BRD-K06426971 | ryuvidine | Histone lysine methyltransferase inhibitor |
| 1522 | 5.02 | cp | BRD-A83326220 | brazilin | Nitric oxide production inhibitor |
| 1520 | 5.04 | cp | BRD-K02950022 | BMS-299897 | Gamma secretase inhibitor |
| 1513 | 5.14 | cp | BRD-K37516142 | idebenone | Calcium channel modulator |
| 1514 | 5.14 | cp | BRD-K18518344 | digitoxigenin | ATPase inhibitor |
| 1507 | 5.25 | cp | BRD-A68723818 | brompheniramine | Histamine receptor antagonist |
| 1508 | 5.25 | cp | BRD-K35687265 | ON-01910 | PLK inhibitor |
| 1506 | 5.29 | cp | BRD-K02992638 | lamivudine | Nucleoside reverse transcriptase inhibitor |
| 1504 | 5.32 | cp | BRD-K29173907 | isoflupredone | Glucocorticoid receptor agonist |
| 1497 | 5.39 | cp | BRD-K88611939 | aniracetam | Glutamate receptor agonist |
| 1496 | 5.4 | cp | BRD-K46862739 | metyrapone | Cytochrome P450 inhibitor |
| 1493 | 5.42 | cp | BRD-A29426959 | carbinoxamine | Histamine receptor antagonist |
| 1489 | 5.45 | cp | BRD-K04185004 | oxybuprocaine | Local anesthetic |
| 1482 | 5.53 | cp | BRD-A35519318 | benidipine | Calcium channel blocker |
| 1484 | 5.53 | cp | BRD-K06080977 | eicosatetraynoic-acid | Cyclooxygenase inhibitor |
| 1476 | 5.58 | cp | BRD-K30867024 | SB-216641 | Serotonin receptor antagonist |
| 1472 | 5.64 | cp | BRD-A59808129 | guggulsterone | Cholesterol inhibitor |
| 1468 | 5.7 | cp | BRD-K55468218 | spiperone | Dopamine receptor antagonist |
| 1463 | 5.78 | cp | BRD-K28761384 | zuclopenthixol | Dopamine receptor antagonist |
| 1461 | 5.8 | cp | BRD-K52020312 | metronidazole | DNA inhibitor |
| 1456 | 5.88 | cp | BRD-A79803969 | memantine | Glutamate receptor antagonist |
| 1457 | 5.88 | cp | BRD-K68143200 | SA-792541 | CDC inhibitor |
| 1451 | 5.93 | cp | BRD-K61951118 | FG-7142 | GABA benzodiazepine site receptor inverse agonist |
| 1448 | 5.95 | cp | BRD-K01779529 | fluoropyruvate | Pyruvate dehydrogenase kinase inhibitor |
| 1442 | 6 | cp | BRD-K01649396 | indatraline | Norepinephrine transporter inhibitor |
| 1437 | 6.06 | cp | BRD-K29733039 | deforolimus | MTOR inhibitor |
| 1431 | 6.15 | cp | BRD-K29582677 | flunarizine | Calcium channel blocker |
| 1429 | 6.17 | cp | BRD-A02710418 | meptazinol | Opioid receptor agonist |
| 1430 | 6.17 | cp | BRD-A68929948 | DAPT-GSI-IX | Gamma secretase inhibitor |
| 1422 | 6.25 | cp | BRD-A41450521 | tosufloxacin | Bacterial DNA gyrase inhibitor |
| 1421 | 6.28 | cp | BRD-K10705233 | GW-405833 | Cannabinoid receptor agonist |
| 1420 | 6.29 | cp | BRD-K08703257 | 3-amino-benzamide | PARP inhibitor |
| 1414 | 6.35 | cp | BRD-A39255369 | DCPIB | Chloride channel blocker |
| 1412 | 6.39 | cp | BRD-K68332390 | ponalrestat | Aldose reductase inhibitor |
| 1409 | 6.45 | cp | BRD-K04923131 | GSK-3-inhibitor-IX | Glycogen synthase kinase inhibitor |
| 1405 | 6.52 | cp | BRD-K59331372 | SB-366791 | TRPV antagonist |
| 1404 | 6.54 | cp | BRD-K83637872 | SANT-1 | Smoothened receptor antagonist |
| 1402 | 6.58 | cp | BRD-A43150328 | penicillic-acid | other antibiotic |
| 1400 | 6.59 | cp | BRD-K11696279 | BU-239 | Imidazoline receptor agonist |
| 1396 | 6.64 | cp | BRD-A39969961 | eplerenone | Cytochrome P450 inhibitor |
| 1395 | 6.66 | cp | BRD-K25310650 | ormetoprim | Bacterial antifolate |
| 1385 | 6.94 | cp | BRD-K93645900 | tadalafil | Phosphodiesterase inhibitor |
| 1384 | 6.95 | cp | BRD-K88849294 | lobaric-acid | Tyrosine phosphatase inhibitor |
| 1380 | 6.99 | cp | BRD-K18036262 | L-168049 | Glucagon receptor antagonist |
| 1379 | 7.01 | cp | BRD-K74761218 | WT-171 | HDAC inhibitor |
| 1375 | 7.03 | cp | BRD-K80725821 | RS-16566 | Serotonin receptor antagonist |
| 1376 | 7.03 | cp | BRD-K83508485 | FK-888 | Tachykinin antagonist |
| 1377 | 7.03 | cp | BRD-K26657438 | imiquimod | TLR agonist |
| 1366 | 7.19 | cp | BRD-K17705806 | JTC-801 | Opioid receptor antagonist |
| 1367 | 7.19 | cp | BRD-A02189320 | met-leu-phe | -666 |
| 1365 | 7.2 | cp | BRD-K52075715 | oxibendazole | Tubulin inhibitor |
| 1360 | 7.31 | cp | BRD-A96255180 | ribavirin | Antiviral |
| 1357 | 7.33 | cp | BRD-M07438658 | lapatinib | EGFR inhibitor |
| 1356 | 7.34 | cp | BRD-A28422058 | L-689560 | Glutamate receptor antagonist |
| 1353 | 7.43 | cp | BRD-K14693417 | cinchonine | P-glycoprotein inhibitor |
| 1352 | 7.45 | cp | BRD-K67637637 | olopatadine | Histamine receptor antagonist |
| 1351 | 7.47 | cp | BRD-A41995253 | brucine | Glycine receptor antagonist |
| 1350 | 7.51 | cp | BRD-A14966924 | alaproclate | Serotonin receptor antagonist |
| 1348 | 7.52 | cp | BRD-K39111395 | BCL2-inhibitor | BCL inhibitor |
| 1347 | 7.56 | cp | BRD-A83937277 | mephenytoin | Hydantoin antiepileptic |
| 1345 | 7.6 | cp | BRD-K43887077 | dopamine | Dopamine receptor agonist |
| 1341 | 7.66 | cp | BRD-K73978287 | hydrocortisone | Glucocorticoid receptor agonist |
| 1338 | 7.71 | cp | BRD-K92428153 | mycophenolate-mofetil | Dehydrogenase inhibitor |
| 1339 | 7.71 | cp | BRD-K04046242 | equilin | Estrogen receptor agonist |
| 1336 | 7.78 | cp | BRD-A01593789 | chlormadinone | 5-alpha reductase inhibitor |
| 1335 | 7.8 | cp | BRD-K64857848 | XMD-885 | Leucine rich repeat kinase inhibitor |
| 1333 | 7.81 | cp | BRD-K03842655 | penitrem-a | Potassium channel blocker |
| 1328 | 7.89 | cp | BRD-K64935403 | ebelactone-b | Lipase inhibitor |
| 1326 | 7.92 | cp | BRD-K04887706 | AKT-inhibitor-1-2 | AKT inhibitor |
| 1324 | 7.97 | cp | BRD-K10467831 | tibolone | Androgen receptor agonist |
| 1319 | 8.05 | cp | BRD-K05396879 | 15-delta-prostaglandin-j2 | PPAR receptor agonist |
| 1318 | 8.07 | cp | BRD-K96778649 | tyrphostin-47 | EGFR inhibitor |
| 1314 | 8.12 | cp | BRD-A18763547 | BAX-channel-blocker | Cytochrome C release inhibitor |
| 1308 | 8.25 | cp | BRD-K03816923 | rottlerin | MAP kinase inhibitor |
| 1306 | 8.26 | cp | BRD-K71926323 | marbofloxacin | Bacterial DNA gyrase inhibitor |
| 1303 | 8.32 | cp | BRD-K43164539 | cholic-acid | Bile acid |
| 1302 | 8.35 | cp | BRD-A70731303 | avrainvillamide-analog-5 | nucleophosmin inhibitor |
| 1301 | 8.36 | cp | BRD-K38305202 | domperidone | Dopamine receptor antagonist |
| 1296 | 8.38 | cp | BRD-K51485625 | ritonavir | HIV protease inhibitor |
| 1297 | 8.38 | cp | BRD-K03642198 | AY-9944 | Hedgehog pathway modulator |
| 1292 | 8.39 | cp | BRD-K68341547 | W-9 | Calmodulin antagonist |
| 1295 | 8.39 | cp | BRD-A23072235 | pheniramine | Histamine receptor antagonist |
| 1290 | 8.45 | cp | BRD-K16336526 | capsaicin | TRPV agonist |
| 1284 | 8.53 | cp | BRD-A95696820 | acadesine | AMPK activator |
| 1282 | 8.56 | cp | BRD-K03406345 | azacitidine | DNA methyltransferase inhibitor |
| 1280 | 8.58 | cp | BRD-A22256192 | terazosin | Adrenergic receptor antagonist |
| 1275 | 8.66 | cp | BRD-K51575138 | TPCA-1 | IKK inhibitor |
| 1272 | 8.67 | cp | BRD-K72029282 | probucol | Atherogenesis inhibitor |
| 1270 | 8.74 | cp | BRD-A43974499 | reboxetine | Adrenergic receptor antagonist |
| 1267 | 8.81 | cp | BRD-K06208435 | YS-035 | Calcium channel blocker |
| 1268 | 8.81 | cp | BRD-K13819402 | desoxypeganine | Acetylcholinesterase inhibitor |
| 1262 | 8.99 | cp | BRD-K56429665 | calcipotriol | Vitamin D receptor agonist |
| 1251 | 9.24 | cp | BRD-K49519144 | LY-2140023 | Glutamate receptor agonist |
| 1247 | 9.3 | cp | BRD-K99291625 | SB-203580 | p38 MAPK inhibitor |
| 1245 | 9.31 | cp | BRD-K06234293 | LY-364947 | TGF beta receptor inhibitor |
| 1246 | 9.31 | cp | BRD-K64052750 | gefitinib | EGFR inhibitor |
| 1243 | 9.38 | cp | BRD-K81847782 | scandenin | Plant compound with antimicrobial activity |
| 1239 | 9.42 | cp | BRD-A89672324 | CGP-55845 | GABA receptor antagonist |
| 1236 | 9.43 | cp | BRD-A62525898 | prednisone | Glucocorticoid receptor agonist |
| 1237 | 9.43 | cp | BRD-K72726508 | arcyriaflavin-a | CDK inhibitor |
| 1234 | 9.46 | cp | BRD-K78637815 | LY-320135 | Cannabinoid receptor antagonist |
| 1230 | 9.61 | cp | BRD-A96107863 | nisoldipine | Calcium channel blocker |
| 1229 | 9.65 | cp | BRD-K27721098 | clopidogrel | Purinergic receptor antagonist |
| 1227 | 9.66 | cp | BRD-K80431395 | triciribine | AKT inhibitor |
| 1226 | 9.67 | cp | BRD-K25186396 | tangeritin | Cell cycle inhibitor |
| 1225 | 9.69 | cp | BRD-A36707673 | hydroxycholesterol | LXR agonist |
| 1222 | 9.73 | cp | BRD-A70268693 | PG-9 | Acetylcholine receptor agonist |
| 1223 | 9.73 | cp | BRD-K43389675 | daunorubicin | RNA synthesis inhibitor |
| 1215 | 9.87 | cp | BRD-K17796732 | JWH-015 | Cannabinoid receptor agonist |
| 1216 | 9.87 | cp | BRD-A16311756 | profenamine | Butyrylcholinesterase inhibitor |
| 1208 | 10.03 | cp | BRD-K42452249 | EO-1428 | p38 MAPK inhibitor |
| 1207 | 10.04 | cp | BRD-K21350491 | phenamil | TRPV antagonist |
| 1205 | 10.08 | cp | BRD-K33193182 | methylnorlichexanthone | Aurora kinase inhibitor |
| 1204 | 10.09 | cp | BRD-K39569857 | avrainvillamide-analog-3 | nucleophosmin inhibitor |
| 1202 | 10.13 | cp | BRD-A10715913 | sulpiride | Dopamine receptor antagonist |
| 1201 | 10.14 | cp | BRD-K15196155 | IBC-293 | Hydroxycarboxylic acid receptor agonist |
| 1199 | 10.17 | cp | BRD-A77349281 | RK-682 | Tyrosine phosphatase inhibitor |
| 1196 | 10.22 | cp | BRD-K56403959 | ZK-756326 | CC chemokine receptor ligand |
| 1193 | 10.25 | cp | BRD-K69690935 | curcumin | Cyclooxygenase inhibitor |
| 1192 | 10.28 | cp | BRD-A83237092 | fulvestrant | Estrogen receptor antagonist |
| 1190 | 10.29 | cp | BRD-K85015012 | NNC-05-2090 | GAT inhibitor |
| 1189 | 10.31 | cp | BRD-K85603128 | resorcinol | Phosphodiesterase inhibitor |
| 1187 | 10.35 | cp | BRD-K10974103 | diloxanide | Protein synthesis inhibitor |
| 1185 | 10.36 | cp | BRD-K14880289 | GW-501516 | PPAR receptor agonist |
| 1181 | 10.5 | cp | BRD-K44876623 | zolpidem | Benzodiazepine receptor agonist |
| 1179 | 10.51 | cp | BRD-K33226500 | indinavir | HIV protease inhibitor |
| 1170 | 10.7 | cp | BRD-K94512704 | spiramide | Dopamine receptor antagonist |
| 1167 | 10.83 | cp | BRD-K21853356 | RG-14620 | EGFR inhibitor |
| 1166 | 10.84 | cp | BRD-K10843433 | phenylbutazone | Cyclooxygenase inhibitor |
| 1165 | 10.87 | cp | BRD-A71765365 | mepireserpate | Catecholamine depleting sympatholytic |
| 1164 | 10.88 | cp | BRD-K82135108 | elesclomol | Oxidative stress inducer |
| 1161 | 10.97 | cp | BRD-A24429032 | HEAT | Adrenergic receptor antagonist |
| 1160 | 11 | cp | BRD-K55344148 | BU-224 | Imidazoline receptor ligand |
| 1158 | 11.02 | cp | BRD-K21565985 | xylazine | Adrenergic receptor agonist |
| 1156 | 11.06 | cp | BRD-K87510569 | RS-504393 | CC chemokine receptor antagonist |
| 1154 | 11.11 | cp | BRD-A72758037 | asiatic-acid | Apoptosis stimulant |
| 1147 | 11.21 | cp | BRD-K12079898 | PD-160170 | Neuropeptide receptor antagonist |
| 1148 | 11.21 | cp | BRD-A41250306 | cyclopenthiazide | Thiazide diuretic |
| 1146 | 11.31 | cp | BRD-A42628519 | iopanoic-acid | Radiopaque medium |
| 1145 | 11.33 | cp | BRD-K10176267 | L-701252 | Glutamate receptor antagonist |
| 1142 | 11.37 | cp | BRD-K29653726 | topiramate | Carbonic anhydrase inhibitor |
| 1140 | 11.41 | cp | BRD-K10573841 | tunicamycin | GLCNAC phosphotransferase inhibitor |
| 1139 | 11.42 | cp | BRD-K13169950 | NSC-3852 | HDAC inhibitor |
| 1137 | 11.51 | cp | BRD-A52326238 | isogedunin | HSP inhibitor |
| 1136 | 11.52 | cp | BRD-K33882852 | ZK-93423 | Benzodiazepine receptor agonist |
| 1133 | 11.53 | cp | BRD-K79877282 | PF-543 | Sphingosine kinase inhibitor |
| 1132 | 11.54 | cp | BRD-A67373739 | AICA-ribonucleotide | AMPK activator |
| 1130 | 11.58 | cp | BRD-K70914287 | BIBX-1382 | EGFR inhibitor |
| 1125 | 11.67 | cp | BRD-K35573744 | erbstatin-analog | EGFR inhibitor |
| 1123 | 11.68 | cp | BRD-A55913614 | primaquine | Antimalarial |
| 1118 | 11.84 | cp | BRD-K04170657 | psoromic-acid | Ras GTPase inhibitor |
| 1117 | 11.86 | cp | BRD-K06543683 | bisindolylmaleimide-ix | CDK inhibitor |
| 1115 | 11.87 | cp | BRD-K91145395 | prostratin | PKC activator |
| 1116 | 11.87 | cp | BRD-A81772229 | simvastatin | HMGCR inhibitor |
| 1113 | 11.89 | cp | BRD-K65910366 | KUC103904N | -666 |
| 1111 | 12.05 | cp | BRD-K92991072 | PAC-1 | Caspase activator |
| 1108 | 12.15 | cp | BRD-K26241953 | piceatannol | SYK inhibitor |
| 1107 | 12.17 | cp | BRD-K95309561 | dienestrol | Estrogen receptor agonist |
| 1106 | 12.19 | cp | BRD-K05464208 | JX-401 | p38 MAPK inhibitor |
| 1104 | 12.2 | cp | BRD-K30697463 | desoximetasone | Glucocorticoid receptor agonist |
| 1105 | 12.2 | cp | BRD-K10136726 | tosyllysyl-chloromethyl-ketone | Chymotrypsin inhibitor |
| 1100 | 12.27 | cp | BRD-K46441700 | GR-55562 | Serotonin receptor antagonist |
| 1099 | 12.28 | cp | BRD-A55484088 | BNTX | Opioid receptor antagonist |
| 1098 | 12.29 | cp | BRD-K50836978 | purvalanol-a | CDK inhibitor |
| 1097 | 12.31 | cp | BRD-A04352665 | maraviroc | CC chemokine receptor antagonist |
| 1093 | 12.45 | cp | BRD-A65767837 | hydrocortisone | Glucocorticoid receptor agonist |
| 1092 | 12.5 | cp | BRD-A88774919 | doxycycline | Bacterial 30S ribosomal subunit inhibitor |
| 1091 | 12.57 | cp | BRD-A36471396 | biperiden | Acetylcholine receptor antagonist |
| 1089 | 12.62 | cp | BRD-K10177585 | PSB-11 | Adenosine receptor antagonist |
| 1085 | 12.68 | cp | BRD-A98378129 | talniflumate | Cyclooxygenase inhibitor |
| 1083 | 12.73 | cp | BRD-K37691127 | hinokitiol | Tyrosinase inhibitor |
| 1078 | 12.88 | cp | BRD-K59773493 | benzohydroxamic-acid | Antifungal |
| 1076 | 12.89 | cp | BRD-K34995470 | SU-1498 | VEGFR inhibitor |
| 1077 | 12.89 | cp | BRD-K39520573 | GW-5074 | Leucine rich repeat kinase inhibitor |
| 1073 | 12.93 | cp | BRD-K79092138 | nitrofural | Bacterial DNA inhibitor |
| 1071 | 12.96 | cp | BRD-K39733634 | L-161982 | Prostanoid receptor antagonist |
| 1069 | 13.05 | cp | BRD-K55591206 | epigallocatechin | Nitric oxide synthase inhibitor |
| 1065 | 13.13 | cp | BRD-K93461745 | buspirone | Serotonin receptor agonist |
| 1062 | 13.22 | cp | BRD-A15493168 | tetracycline | Bacterial 30S ribosomal subunit inhibitor |
| 1058 | 13.3 | cp | BRD-K28428262 | brivanib | FGFR inhibitor |
| 1055 | 13.38 | cp | BRD-K06817181 | BRD-K06817181 | JAK inhibitor |
| 1054 | 13.4 | cp | BRD-K83063356 | RS-102895 | CCR antagonist |
| 1046 | 13.68 | cp | BRD-K15086322 | JNJ-10191584 | Histamine receptor antagonist |
| 1044 | 13.7 | cp | BRD-K91696562 | orantinib | FGFR inhibitor |
| 1038 | 13.75 | cp | BRD-A10969569 | ambelline | Plant alkaloid |
| 1035 | 13.83 | cp | BRD-A07824748 | flavanone | 11-beta-HSD1 inhibitor |
| 1034 | 13.87 | cp | BRD-K00317371 | RITA | MDM inhibitor |
| 1029 | 14.06 | cp | BRD-K11927976 | ER-27319 | Mediator release inhibitor |
| 1028 | 14.08 | cp | BRD-K68246049 | TTNPB | Retinoid receptor agonist |
| 1026 | 14.25 | cp | BRD-K11853856 | PJ-34 | PARP inhibitor |
| 1021 | 14.38 | cp | BRD-A56892734 | esomeprazole | ATPase inhibitor |
| 1020 | 14.43 | cp | BRD-A92177080 | betamethasone | Glucocorticoid receptor agonist |
| 1012 | 14.78 | cp | BRD-K94649603 | taxifolin | Opioid receptor antagonist |
| 1010 | 14.86 | cp | BRD-K15519488 | CS-110266 | Dopamine receptor agonist |
| 995 | 15.36 | cp | BRD-K83988098 | alvespimycin | HSP inhibitor |
| 994 | 15.41 | cp | BRD-K62736196 | guanabenz | -666 |
| 993 | 15.51 | cp | BRD-A19195498 | trimipramine | Norepinephrine reuptake inhibitor |
| 989 | 15.55 | cp | BRD-K30097969 | pitavastatin | HMGCR inhibitor |
| 986 | 15.61 | cp | BRD-K99964838 | bosutinib | ABL inhibitor |
| 987 | 15.61 | cp | BRD-K21806131 | tegaserod | Serotonin receptor partial agonist |
| 983 | 15.67 | cp | BRD-K08219523 | 5-nonyloxytryptamine | Serotonin receptor agonist |
| 982 | 15.68 | cp | BRD-K48722258 | dilazep | Adenosine reuptake inhibitor |
| 981 | 15.71 | cp | BRD-K96119599 | leucodin | Melanin inhibitor |
| 980 | 15.74 | cp | BRD-A04308630 | genipin | Choleretic agent |
| 979 | 15.76 | cp | BRD-K97399794 | quercetin | Polar auxin transport inhibitor |
| 977 | 15.85 | cp | BRD-K31611373 | fluprostenol | Prostanoid receptor agonist |
| 974 | 15.92 | cp | BRD-K06980535 | promazine | Dopamine receptor antagonist |
| 973 | 15.99 | cp | BRD-K93258693 | GW-9662 | PPAR receptor antagonist |
| 970 | 16.19 | cp | BRD-K41564320 | purvalanol-b | Tyrosine kinase inhibitor |
| 966 | 16.28 | cp | BRD-K47192521 | icosapent | Platelet aggregation inhibitor |
| 963 | 16.32 | cp | BRD-K19295594 | gossypol | BCL inhibitor |
| 961 | 16.36 | cp | BRD-K33211335 | dextromethorphan | Glutamate receptor antagonist |
| 958 | 16.5 | cp | BRD-A06784547 | MRS-1334 | Adenosine receptor antagonist |
| 955 | 16.53 | cp | BRD-K75295174 | alisertib | Aurora kinase inhibitor |
| 956 | 16.53 | cp | BRD-A78295502 | hydroquinine | Antiarrhythmic |
| 951 | 16.61 | cp | BRD-K57631554 | aminolevulinic-acid | Oxidizing agent |
| 949 | 16.66 | cp | BRD-K82941592 | rosuvastatin | HMGCR inhibitor |
| 946 | 16.73 | cp | BRD-U44618005 | WH-4023 | SRC inhibitor |
| 945 | 16.74 | cp | BRD-A34205397 | suloctidil | Adrenergic receptor antagonist |
| 943 | 16.88 | cp | BRD-K30990140 | FR-122047 | Cyclooxygenase inhibitor |
| 939 | 16.93 | cp | BRD-K01624546 | docosatrienoic-acid | LTB4 inhibitor |
| 940 | 16.93 | cp | BRD-K39120595 | bithionol | Autotaxin inhibitor |
| 938 | 16.94 | cp | BRD-K30649484 | mafenide | Carbonic anhydrase inhibitor |
| 936 | 17.02 | cp | BRD-A25775766 | securinine | GABA receptor antagonist |
| 934 | 17.09 | cp | BRD-K46137903 | prednicarbate | Phospholipase activator |
| 932 | 17.12 | cp | BRD-A00758722 | noretynodrel | Progestogen hormone |
| 931 | 17.16 | cp | BRD-K63979671 | etifenin | Compound used in hepatobiliary scans of the liver |
| 930 | 17.18 | cp | BRD-A80502530 | cinobufagin | ATPase inhibitor |
| 925 | 17.33 | cp | BRD-K21680192 | mitoxantrone | Topoisomerase inhibitor |
| 924 | 17.44 | cp | BRD-K43764301 | dexketoprofen | Cyclooxygenase inhibitor |
| 923 | 17.56 | cp | BRD-K41445866 | alfaxalone | Chloride channel agonist |
| 921 | 17.67 | cp | BRD-K70881766 | solanine | Acetylcholinesterase inhibitor |
| 918 | 17.83 | cp | BRD-K47679368 | bromfenac | Cyclooxygenase inhibitor |
| 913 | 17.88 | cp | BRD-K86873305 | piperacillin | Bacterial cell wall synthesis inhibitor |
| 914 | 17.88 | cp | BRD-K04430056 | 7-nitroindazole | nitric oxide synthase inhibitor |
| 910 | 17.98 | cp | BRD-K85242180 | beta-CCP | Indoleamine 2,3-dioxygenase inhibitor |
| 907 | 18.08 | cp | BRD-K99411983 | lumicolchicine | Colchicine isomer, non-binder of microtubules |
| 905 | 18.14 | cp | BRD-K64800655 | PHA-793887 | CDK inhibitor |
| 901 | 18.28 | cp | BRD-K80396088 | gliquidone | Sulfonylurea |
| 897 | 18.33 | cp | BRD-K82109576 | vincristine | Tubulin inhibitor |
| 895 | 18.48 | cp | BRD-K92492521 | LY-255283 | Leukotriene receptor antagonist |
| 894 | 18.55 | cp | BRD-K78659596 | MLN-2238 | Proteasome inhibitor |
| 890 | 18.72 | cp | BRD-K33483813 | actarit | Interleukin receptor agonist |
| 885 | 18.91 | cp | BRD-K66766661 | 17-beta-estradiol | Estrogen receptor agonist |
| 884 | 19.03 | cp | BRD-K33860217 | CP-94253 | Serotonin receptor agonist |
| 878 | 19.36 | cp | BRD-K40645748 | mefloquine | Adenosine receptor antagonist |
| 875 | 19.38 | cp | BRD-K85985071 | ellipticine | Topoisomerase inhibitor |
| 872 | 19.47 | cp | BRD-A39268308 | epibatidine | Acetylcholine receptor agonist |
| 871 | 19.48 | cp | BRD-K29673530 | hypericin | Tyrosine kinase inhibitor |
| 870 | 19.49 | cp | BRD-K43978949 | PIT | Purinergic receptor antagonist |
| 869 | 19.51 | cp | BRD-A52282606 | lacidipine | Calcium channel blocker |
| 868 | 19.62 | cp | BRD-K18787491 | U-0126 | MEK inhibitor |
| 867 | 19.64 | cp | BRD-K31238592 | devazepide | CCK receptor antagonist |
| 866 | 19.78 | cp | BRD-K04546108 | JAK3-inhibitor-VI | JAK inhibitor |
| 865 | 19.79 | cp | BRD-K60690191 | MPEP | Glutamate receptor antagonist |
| 864 | 19.8 | cp | BRD-K63784565 | BRD-K63784565 | Topoisomerase inhibitor |
| 861 | 20.02 | cp | BRD-K24201553 | SB-269970 | Serotonin receptor antagonist |
| 856 | 20.11 | cp | BRD-K99545815 | PF-562271 | Focal adhesion kinase inhibitor |
| 851 | 20.29 | cp | BRD-K81916719 | triclabendazole | Microtubule inhibitor |
| 850 | 20.33 | cp | BRD-K82255054 | propofol | GABA receptor agonist |
| 849 | 20.35 | cp | BRD-A65449987 | flunisolide | Cytochrome P450 inhibitor |
| 847 | 20.37 | cp | BRD-A85860691 | chaetocin | Histone lysine methyltransferase inhibitor |
| 841 | 20.54 | cp | BRD-A18579359 | wiskostatin | Neural Wiskott-Aldrich syndrome protein inhibitor |
| 837 | 20.68 | cp | BRD-K88701661 | dimercaptosuccinic-acid | Chelating agent |
| 835 | 20.75 | cp | BRD-A13122391 | triptolide | RNA polymerase inhibitor |
| 833 | 20.78 | cp | BRD-K78838262 | austricine | Hypolipidemic |
| 832 | 20.8 | cp | BRD-K62206109 | VUF-5681 | Histamine receptor antagonist |
| 831 | 20.82 | cp | BRD-K66093087 | FGIN-1-43 | Benzodiazepine receptor agonist |
| 829 | 20.86 | cp | BRD-K41868777 | W-5 | Calmodulin antagonist |
| 828 | 20.87 | cp | BRD-A63836183 | PD-123319 | Angiotensin receptor antagonist |
| 824 | 21.01 | cp | BRD-K76698671 | HNHA | HDAC inhibitor |
| 822 | 21.07 | cp | BRD-K70301876 | escitalopram | Selective serotonin reuptake inhibitor (SSRI) |
| 818 | 21.11 | cp | BRD-K90333595 | phentolamine | Adrenergic receptor antagonist |
| 815 | 21.45 | cp | BRD-A29437505 | RWJ-21757 | TLR agonist |
| 813 | 21.57 | cp | BRD-K53790871 | triamcinolone | Glucocorticoid receptor agonist |
| 812 | 21.72 | cp | BRD-K13800121 | parecoxib | Cyclooxygenase inhibitor |
| 811 | 21.75 | cp | BRD-K12357156 | AG-490 | EGFR inhibitor |
| 801 | 22.23 | cp | BRD-K05926469 | lenalidomide | Antineoplastic |
| 800 | 22.24 | cp | BRD-K17075857 | chloroxine | Opioid receptor antagonist |
| 799 | 22.25 | cp | BRD-A27887842 | prednisolone | Glucocorticoid receptor agonist |
| 796 | 22.52 | cp | BRD-K43245338 | MDL-28170 | Calpain inhibitor |
| 795 | 22.59 | cp | BRD-K87049188 | fusaric-acid | Dopamine beta hydroxylase inhibitor |
| 793 | 22.72 | cp | BRD-K94919853 | 10H-phenothiazin-10-yl)(p-tolyl)methanone | Butyrylcholinesterase inhibitor |
| 789 | 22.9 | cp | BRD-K98521173 | desoxycortone | Mineralocorticoid receptor agonist |
| 788 | 22.95 | cp | BRD-A10355991 | norketamine | Glutamate receptor antagonist |
| 783 | 23.14 | cp | BRD-K65285700 | BRD-K65285700 | Cannabinoid receptor agonist |
| 782 | 23.21 | cp | BRD-K70505054 | ranitidine | Histamine receptor antagonist |
| 780 | 23.3 | cp | BRD-K59650319 | YM-298198 | Glutamate receptor antagonist |
| 779 | 23.32 | cp | BRD-K95899059 | LY-344864 | Serotonin receptor agonist |
| 774 | 23.67 | cp | BRD-K26548821 | quinpirole | Dopamine receptor agonist |
| 773 | 23.68 | cp | BRD-K89930444 | AG-592 | Tyrosine kinase inhibitor |
| 771 | 23.7 | cp | BRD-A04756508 | norgestimate | Progesterone receptor agonist |
| 769 | 23.72 | cp | BRD-K46068882 | eugenitol | Bacterial quorum sensing inhibitor |
| 766 | 23.78 | cp | BRD-K07507905 | BRL-37344 | Adrenergic receptor agonist |
| 764 | 23.91 | cp | BRD-A15415227 | GW-1929 | PPAR receptor agonist |
| 762 | 24.02 | cp | BRD-K99946902 | hexylresorcinol | Local anesthetic |
| 761 | 24.1 | cp | BRD-K73982490 | BI-78D3 | JNK inhibitor |
| 760 | 24.13 | cp | BRD-A34806832 | proscillaridin | ATPase inhibitor |
| 759 | 24.18 | cp | BRD-K62012036 | acitretin | Retinoid receptor agonist |
| 754 | 24.53 | cp | BRD-K54330070 | SB-202190 | p38 MAPK inhibitor |
| 753 | 24.54 | cp | BRD-K22096725 | ALW-II-49-7 | Ephrin inhibitor |
| 746 | 25.13 | cp | BRD-K50214219 | CS-1657 | PARP inhibitor |
| 745 | 25.16 | cp | BRD-A08709697 | heliotrine | Pyrrolizidine alkaloid |
| 741 | 25.36 | cp | BRD-K30570479 | VU-0400195-3 | Glutamate receptor modulator |
| 740 | 25.43 | cp | BRD-K36009368 | NNC-63-0532 | Opioid receptor agonist |
| 738 | 25.64 | cp | BRD-A44551378 | LFM-A12 | EGFR inhibitor |
| 737 | 25.66 | cp | BRD-K35458079 | edaravone | Nootropic agent |
| 736 | 25.7 | cp | BRD-K92428232 | GSK-461364 | PLK inhibitor |
| 735 | 25.75 | cp | BRD-K91442916 | CAM-9-026 | Membrane metalloendopeptidase inhibitor |
| 733 | 25.84 | cp | BRD-K30296925 | flavokavain-b | Antineoplastic |
| 728 | 26.02 | cp | BRD-K21672174 | RO-28-1675 | Glucokinase activator |
| 724 | 26.23 | cp | BRD-A49906757 | scopolamine | Acetylcholine receptor antagonist |
| 717 | 26.46 | cp | BRD-K33308633 | INCA-6 | Calcineurin inhibitor |
| 710 | 26.98 | cp | BRD-A95513702 | androsta-1,4-dien-3,17-dione | Aromatase inhibitor |
| 709 | 27.01 | cp | BRD-K19309090 | SR-95639A | Acetylcholine receptor agonist |
| 703 | 27.24 | cp | BRD-U73238814 | QL-XI-92 | DDR1 inhibitor |
| 698 | 27.53 | cp | BRD-K00312224 | PPT | Estrogen receptor agonist |
| 695 | 27.63 | cp | BRD-K17415526 | tyrphostin-AG-835 | Protein tyrosine kinase inhibitor |
| 689 | 28.19 | cp | BRD-K20742498 | RS-39604 | Serotonin receptor antagonist |
| 688 | 28.2 | cp | BRD-K16406336 | methylene-blue | Guanylyl cyclase inhibitor |
| 681 | 28.35 | cp | BRD-A35989968 | megestrol | Progesterone receptor agonist |
| 680 | 28.36 | cp | BRD-K19894101 | MST-312 | Telomerase inhibitor |
| 674 | 28.84 | cp | BRD-K33572481 | taurodeoxycholic-acid | Bile acid |
| 673 | 28.96 | cp | BRD-K47780086 | penciclovir | DNA directed DNA polymerase inhibitor |
| 671 | 29.07 | cp | BRD-K32906660 | bis-tyrphostin | EGFR inhibitor |
| 669 | 29.15 | cp | BRD-A69960130 | bromocriptine | Dopamine receptor agonist |
| 668 | 29.19 | cp | BRD-A15914070 | 4-hydroxy-2-nonenal | Cytotoxic lipid peroxidation product |
| 667 | 29.21 | cp | BRD-K02581333 | protein-tyrosine-phosphatase-inhibitor-IV | Tyrosine phosphatase inhibitor |
| 665 | 29.27 | cp | BRD-A80775386 | hyperforin | Cyclooxygenase inhibitor |
| 662 | 29.71 | cp | BRD-A25004090 | erastin | Ion channel antagonist |
| 660 | 29.85 | cp | BRD-K93095519 | SJ-172550 | MDM inhibitor |
| 655 | 30.11 | cp | BRD-A82590476 | SDZ-NKT-343 | Tachykinin antagonist |
| 651 | 30.36 | cp | BRD-K32311154 | nifekalant | Potassium channel blocker |
| 650 | 30.52 | cp | BRD-K43797669 | genistein | Tyrosine kinase inhibitor |
| 649 | 30.6 | cp | BRD-K98493452 | honokiol | AKT inhibitor |
| 646 | 30.87 | cp | BRD-K72676686 | fluvoxamine | Selective serotonin reuptake inhibitor (SSRI) |
| 644 | 30.92 | cp | BRD-K49294207 | BIBU-1361 | EGFR inhibitor |
| 643 | 31 | cp | BRD-A35033682 | eriodictyol | Cytochrome P450 inhibitor |
| 640 | 31.1 | cp | BRD-K67013324 | luzindole | Melatonin receptor antagonist |
| 639 | 31.17 | cp | BRD-A80383043 | BRD-A80383043 | Glutamate receptor agonist |
| 635 | 31.36 | cp | BRD-A04327189 | synephrine | Adrenergic receptor agonist |
| 632 | 31.43 | cp | BRD-K50938287 | sumatriptan | Serotonin receptor agonist |
| 631 | 31.45 | cp | BRD-K64517075 | heliomycin | ATP synthase inhibitor |
| 630 | 31.47 | cp | BRD-K66206289 | lobeline | Acetylcholine receptor antagonist |
| 627 | 31.9 | cp | BRD-A54927599 | KF-38789 | P-selectin inhibitor |
| 624 | 32.06 | cp | BRD-K41903098 | diphenoxylate | Opioid receptor agonist |
| 623 | 32.09 | cp | BRD-K71266197 | PSB-06126 | NTPDase inhibitor |
| 621 | 32.21 | cp | BRD-A72988804 | tiaprofenic-acid | Cyclooxygenase inhibitor |
| 620 | 32.35 | cp | BRD-K85090592 | pilocarpine | Acetylcholine receptor agonist |
| 614 | 32.57 | cp | BRD-K62200014 | anagrelide | Phosphodiesterase inhibitor |
| 612 | 32.61 | cp | BRD-K11107424 | tiotidine | Histamine receptor antagonist |
| 611 | 32.65 | cp | BRD-K94353609 | fluocinolone | Glucocorticoid receptor agonist |
| 608 | 32.99 | cp | BRD-K11558771 | droxinostat | HDAC inhibitor |
| 607 | 33 | cp | BRD-K28168037 | fenretinide | Apoptosis stimulant |
| 602 | 33.26 | cp | BRD-K08502430 | angiogenesis-inhibitor | Angiogenesis inhibitor |
| 599 | 33.38 | cp | BRD-K19284129 | salvinorin-a | Opioid receptor agonist |
| 592 | 33.79 | cp | BRD-K32398298 | alprazolam | Benzodiazepine receptor agonist |
| 591 | 33.95 | cp | BRD-K04146668 | GW-441756 | Growth factor receptor inhibitor |
| 586 | 34.18 | cp | BRD-K19605405 | ZM-241385 | Adenosine receptor antagonist |
| 585 | 34.3 | cp | BRD-K14821540 | FCCP | Mitochondrial oxidative phosphorylation uncoupler |
| 578 | 34.91 | cp | BRD-K84996949 | sinensetin | Cyclooxygenase inhibitor |
| 575 | 35.08 | cp | BRD-K40758068 | efavirenz | HIV protease inhibitor |
| 574 | 35.09 | cp | BRD-K32584078 | BML-257 | AKT inhibitor |
| 572 | 35.19 | cp | BRD-K41337261 | ZM-306416 | ABL inhibitor |
| 571 | 35.24 | cp | BRD-K36395411 | SB-206553 | Serotonin receptor antagonist |
| 570 | 35.28 | cp | BRD-K73991644 | isoquercetin | Aldose reductase inhibitor |
| 565 | 35.71 | cp | BRD-K22010301 | JLK-6 | Gamma secretase inhibitor |
| 562 | 35.86 | cp | BRD-K68336408 | tyrphostin-AG-1478 | EGFR inhibitor |
| 561 | 35.88 | cp | BRD-K36198571 | WAY-170523 | Metalloproteinase inhibitor |
| 560 | 35.93 | cp | BRD-K56301217 | ABT-737 | BCL inhibitor |
| 555 | 36.4 | cp | BRD-A07765530 | epinephrine | carbonic anhydrase activator |
| 553 | 36.46 | cp | BRD-A42346008 | metanephrine | Epinephrine metabolite |
| 551 | 36.53 | cp | BRD-K01253243 | SB-590885 | RAF inhibitor |
| 550 | 36.54 | cp | BRD-A54029483 | IRL-2500 | Endothelin receptor antagonist |
| 542 | 36.97 | cp | BRD-K63504947 | semaxanib | VEGFR inhibitor |
| 541 | 37 | cp | BRD-K97309399 | thiothixene | Dopamine receptor antagonist |
| 537 | 37.42 | cp | BRD-K73395020 | SA-1478088 | -666 |
| 534 | 37.49 | cp | BRD-A37052580 | physostigmine | Acetylcholinesterase inhibitor |
| 532 | 37.58 | cp | BRD-K88625236 | nonoxynol-9 | Membrane integrity inhibitor |
| 530 | 37.7 | cp | BRD-K49657628 | tyrphostin-AG-18 | EGFR inhibitor |
| 529 | 37.72 | cp | BRD-K44849676 | capsazepine | TRPV agonist |
| 527 | 37.82 | cp | BRD-K84141129 | VU-0400193-3 | Glutamate receptor modulator |
| 521 | 38.42 | cp | BRD-K38477985 | malonoben | Protein tyrosine kinase inhibitor |
| 519 | 38.59 | cp | BRD-A80641450 | FR-139317 | Endothelin receptor antagonist |
| 517 | 38.64 | cp | BRD-K27665173 | D-64406 | PDGFR receptor inhibitor |
| 514 | 38.73 | cp | BRD-K61737877 | VEGF-receptor-2-kinase-inhibitor-IV | VEGFR inhibitor |
| 513 | 38.77 | cp | BRD-K91336023 | mesulergine | Dopamine receptor agonist |
| 512 | 38.8 | cp | BRD-K33453211 | levocabastine | Histamine receptor antagonist |
| 509 | 38.9 | cp | BRD-K34098590 | tienilic-acid | Sodium/potassium/chloride transporter inhibitor |
| 507 | 39.25 | cp | BRD-K63150726 | JTE-907 | Cannabinoid receptor inverse agonist |
| 505 | 39.35 | cp | BRD-K73290745 | ICI-199441 | Opioid receptor agonist |
| 504 | 39.43 | cp | BRD-K09764130 | mead-ethanolamide | Cannabinoid receptor agonist |
| 500 | 39.73 | cp | BRD-K34092021 | arvanil | TRPV agonist |
| 499 | 39.77 | cp | BRD-A35108200 | dexamethasone | Glucocorticoid receptor agonist |
| 496 | 39.82 | cp | BRD-A13807286 | HA-14-1 | BCL inhibitor |
| 492 | 40.37 | cp | BRD-A82656074 | naltrindole | Opioid receptor antagonist |
| 491 | 40.4 | cp | BRD-A57382968 | piroxicam | Cyclooxygenase inhibitor |
| 487 | 40.68 | cp | BRD-M30523314 | vinorelbine | Tubulin inhibitor |
| 486 | 40.69 | cp | BRD-A09539288 | homatropine | Acetylcholine receptor antagonist |
| 485 | 40.75 | cp | BRD-K62221994 | T-98475 | Gonadotropin releasing factor hormone receptor antagonist |
| 484 | 40.78 | cp | BRD-K67298865 | SB-431542 | TGF beta receptor inhibitor |
| 481 | 40.96 | cp | BRD-M47937986 | cefatrizine | Bacterial cell wall synthesis inhibitor |
| 478 | 41.1 | cp | BRD-A11007541 | BCI-hydrochloride | Protein phosphatase inhibitor |
| 474 | 41.32 | cp | BRD-K75478907 | GS-39783 | GABA receptor modulator |
| 470 | 41.4 | cp | BRD-K18194590 | mephentermine | Adrenergic receptor agonist |
| 469 | 41.48 | cp | BRD-K39944607 | ochratoxin-a | Phenylalanyl tRNA synthetase inhibitor |
| 465 | 41.63 | cp | BRD-K28667196 | fillalbin | Increases arterial blood pressure |
| 463 | 41.93 | cp | BRD-K19796430 | erismodegib | Smoothened receptor antagonist |
| 457 | 42.59 | cp | BRD-K29582115 | ziprasidone | Dopamine receptor antagonist |
| 456 | 42.69 | cp | BRD-K31843556 | T-0070907 | PPAR receptor antagonist |
| 455 | 42.75 | cp | BRD-A77118605 | BML-ST330 | Phospholipase inhibitor |
| 453 | 42.8 | cp | BRD-K61691971 | avrainvillamide-analog-1 | nucleophosmin inhibitor |
| 448 | 43.31 | cp | BRD-K53665955 | MK-5108 | Aurora kinase inhibitor |
| 438 | 44.33 | cp | BRD-K47328134 | lysylphenylalanyl-tyrosine | Heparin activation inhibitor |
| 429 | 44.82 | cp | BRD-A38749782 | fludrocortisone | Glucocorticoid receptor agonist |
| 428 | 44.93 | cp | BRD-K49810818 | sorafenib | FLT3 inhibitor |
| 427 | 45.24 | cp | BRD-K63606607 | bufalin | ATPase inhibitor |
| 423 | 45.75 | cp | BRD-K17497770 | butein | EGFR inhibitor |
| 420 | 45.88 | cp | BRD-K56700933 | phenethyl-isothiocyanate | Antineoplastic |
| 419 | 45.94 | cp | BRD-K50720187 | flupirtine | Glutamate receptor antagonist |
| 416 | 46.11 | cp | BRD-K82846253 | repaglinide | Insulin secretagogue |
| 412 | 46.42 | cp | BRD-K99498722 | NPI-2358 | Tubulin inhibitor |
| 411 | 46.73 | cp | BRD-K19111024 | clofibric-acid | PPAR receptor agonist |
| 408 | 46.9 | cp | BRD-A62809825 | thapsigargin | ATPase inhibitor |
| 407 | 46.97 | cp | BRD-A20126139 | medrysone | Glucocorticoid receptor agonist |
| 404 | 47.07 | cp | BRD-A42167015 | carteolol | Adrenergic receptor antagonist |
| 401 | 47.36 | cp | BRD-K92758126 | gibberellic-acid | NFkB pathway inhibitor |
| 400 | 47.43 | cp | BRD-A82238138 | budesonide | Glucocorticoid receptor agonist |
| 397 | 47.85 | cp | BRD-A17065207 | brefeldin-a | Protein synthesis inhibitor |
| 395 | 47.95 | cp | BRD-K84987553 | MDM2-inhibitor | MDM inhibitor |
| 394 | 47.97 | cp | BRD-M86331534 | pyrvinium-pamoate | AKT inhibitor |
| 388 | 48.29 | cp | BRD-K09638361 | SA-63133 | -666 |
| 385 | 48.45 | cp | BRD-K40919711 | BAPTA-AM | Potassium channel blocker |
| 383 | 48.54 | cp | BRD-A46186775 | hydrocortisone | Glucocorticoid receptor agonist |
| 381 | 48.67 | cp | BRD-K55677650 | CO-101244 | Ionotropic glutamate receptor antagonist |
| 378 | 48.95 | cp | BRD-K00610438 | altanserin | Serotonin receptor antagonist |
| 377 | 48.97 | cp | BRD-K64670467 | JNJ-16259685 | Glutamate receptor antagonist |
| 376 | 49 | cp | BRD-K02607075 | tubocurarine | Acetylcholine receptor antagonist |
| 369 | 50.01 | cp | BRD-K37456065 | VU-0365114-2 | M5 modulator |
| 364 | 50.08 | cp | BRD-K69763916 | LY-341495 | Glutamate receptor antagonist |
| 362 | 50.15 | cp | BRD-K76872913 | benzanthrone | Aromatic hydrocarbon derivative |
| 351 | 51.69 | cp | BRD-A78360835 | cercosporin | Photoactivated toxin |
| 349 | 52.04 | cp | BRD-A16478930 | amcinonide | Glucocorticoid receptor agonist |
| 342 | 53.22 | cp | BRD-K26818574 | BIX-01294 | Histone lysine methyltransferase inhibitor |
| 337 | 53.67 | cp | BRD-K19416115 | sitagliptin | Dipeptidyl peptidase inhibitor |
| 334 | 54.18 | cp | BRD-K97764662 | PD-173074 | FGFR inhibitor |
| 328 | 54.73 | cp | BRD-A92439610 | triamcinolone | Glucocorticoid receptor agonist |
| 324 | 54.87 | cp | BRD-K55191674 | benzylpenicillin | Penicillin binding protein inhibitor |
| 323 | 55.03 | cp | BRD-K40255344 | tyrphostin-A9 | Protein tyrosine kinase inhibitor |
| 319 | 55.47 | cp | BRD-A41555725 | chlortetracycline | Protein synthesis inhibitor |
| 318 | 55.52 | cp | BRD-A15079084 | phorbol-12-myristate-13-acetate | PKC activator |
| 315 | 55.66 | cp | BRD-K12539581 | nocodazole | Tubulin inhibitor |
| 313 | 55.77 | cp | BRD-K92301463 | 16,16-dimethylprostaglandin-e2 | Prostanoid receptor agonist |
| 306 | 56.3 | cp | BRD-K82091397 | SB-239063 | p38 MAPK inhibitor |
| 303 | 56.51 | cp | BRD-A01346607 | flumetasone | Glucocorticoid receptor agonist |
| 301 | 56.56 | cp | BRD-K24681473 | YM-155 | Survivin inhibitor |
| 300 | 56.61 | cp | BRD-K90864987 | cobalt(II)-chloride | HSP inducer |
| 298 | 56.78 | cp | BRD-A28105619 | cucurbitacin-i | JAK inhibitor |
| 294 | 57.43 | cp | BRD-A50675702 | fipronil | GABA gated chloride channel blocker |
| 290 | 57.56 | cp | BRD-A15297126 | fluocinonide | Glucocorticoid receptor agonist |
| 289 | 57.7 | cp | BRD-K17674993 | diflorasone | Corticosteroid agonist |
| 285 | 57.95 | cp | BRD-A50737080 | CGK-733 | ATR kinase inhibitor |
| 279 | 59.26 | cp | BRD-K30189597 | SYK-inhibitor | SYK inhibitor |
| 276 | 59.52 | cp | BRD-K96263742 | GW-7647 | PPAR receptor agonist |
| 275 | 59.53 | cp | BRD-K09436313 | prostaglandin | Prostanoid receptor antagonist |
| 273 | 59.75 | cp | BRD-K96799727 | pifithrin-mu | HSP inhibitor |
| 268 | 60.52 | cp | BRD-K85606544 | neratinib | EGFR inhibitor |
| 257 | 61.2 | cp | BRD-K03440695 | boldine | Acetylcholine receptor antagonist |
| 256 | 61.38 | cp | BRD-K76907295 | VU-0418947-2 | HIF modulator |
| 254 | 61.63 | cp | BRD-K44432556 | VU-0418946-1 | HIF modulator |
| 252 | 61.98 | cp | BRD-K88868628 | iodoacetic-acid | Cysteine peptidase inhibitor |
| 247 | 62.37 | cp | BRD-K26997899 | SA-792574 | Microtubule inhibitor |
| 246 | 62.62 | cp | BRD-K13566078 | BMS-345541 | IKK inhibitor |
| 245 | 62.63 | cp | BRD-A62184259 | cycloheximide | Protein synthesis inhibitor |
| 242 | 63.23 | cp | BRD-K51816706 | oxindole-I | VEGFR inhibitor |
| 239 | 63.67 | cp | BRD-A35623999 | CGP-37157 | L-type calcium channel blocker |
| 236 | 63.91 | cp | BRD-K52662033 | lidocaine | Histamine receptor agonist |
| 232 | 64.87 | cp | BRD-K05653692 | DL-PDMP | Glucosyltransferase inhibitor |
| 230 | 65.15 | cp | BRD-K09859624 | methantheline | Acetylcholine receptor antagonist |
| 229 | 65.25 | cp | BRD-A94756469 | digoxin | ATPase inhibitor |
| 227 | 65.72 | cp | BRD-K11433652 | aspirin | Cyclooxygenase inhibitor |
| 225 | 65.88 | cp | BRD-A23637604 | oxymetholone | Androgen receptor agonist |
| 223 | 66.25 | cp | BRD-A73605923 | mocimycin | Protein synthesis inhibitor |
| 219 | 66.49 | cp | BRD-A48570745 | ivermectin | GABA receptor agonist |
| 218 | 66.5 | cp | BRD-K56614220 | clofazimine | GK0582 inhibitor |
| 214 | 67.1 | cp | BRD-K33551950 | radicicol | HSP inhibitor |
| 209 | 67.55 | cp | BRD-K77133231 | PD-169316 | p38 MAPK inhibitor |
| 201 | 69.22 | cp | BRD-A73741725 | exemestane | Aromatase inhibitor |
| 200 | 69.48 | cp | BRD-K33583600 | isoliquiritigenin | Guanylate cyclase activator |
| 196 | 69.84 | cp | BRD-K76064317 | tyrphostin-AG-1296 | FLT3 inhibitor |
| 195 | 69.93 | cp | BRD-K50311478 | tosyl-phenylalanyl-chloromethyl-ketone | Chymotrypsin inhibitor |
| 194 | 69.95 | cp | BRD-A60197193 | amisulpride | Dopamine receptor antagonist |
| 183 | 71.29 | cp | BRD-K55430733 | WAY-629 | Serotonin receptor agonist |
| 181 | 71.48 | cp | BRD-K77987382 | mebendazole | Tubulin inhibitor |
| 173 | 72.67 | cp | BRD-K15600710 | obatoclax | BCL inhibitor |
| 167 | 73.06 | cp | BRD-A64479082 | quinidine | Sodium channel blocker |
| 166 | 73.1 | cp | BRD-K05977823 | tenovins | SIRT inhibitor |
| 164 | 73.31 | cp | BRD-M64432851 | sunitinib | FLT3 inhibitor |
| 160 | 73.39 | cp | BRD-K26669427 | WR-216174 | PFMRK inhibitor |
| 156 | 73.87 | cp | BRD-K06878038 | deferiprone | Chelating agent |
| 153 | 74.17 | cp | BRD-K41160163 | fenobam | Glutamate receptor antagonist |
| 152 | 74.26 | cp | BRD-K43236057 | piceid | ICAM1 inhibitor |
| 149 | 74.71 | cp | BRD-A93000692 | ciglitazone | PPAR receptor agonist |
| 138 | 76.16 | cp | BRD-A52650764 | ingenol | PKC activator |
| 136 | 76.3 | cp | BRD-K26117720 | gingerol | Nitric oxide synthase inhibitor |
| 133 | 76.33 | cp | BRD-K51290057 | SA-792709 | Retinoid receptor agonist |
| 130 | 76.86 | cp | BRD-K03557653 | sappanone-a | Tyrosinase inhibitor |
| 125 | 77.54 | cp | BRD-K82823804 | SA-792987 | PKC inhibitor |
| 124 | 78.06 | cp | BRD-K05901394 | terguride | Dopamine receptor agonist |
| 111 | 80.32 | cp | BRD-K80970344 | pyrrolidine-dithiocarbamate | NFkB pathway inhibitor |
| 108 | 80.88 | cp | BRD-K85402309 | dovitinib | EGFR inhibitor |
| 106 | 80.95 | cp | BRD-K86003836 | flubendazole | Tubulin inhibitor |
| 104 | 81.3 | cp | BRD-A56020723 | CA-074-Me | Cathepsin inhibitor |
| 101 | 81.66 | cp | BRD-K83289131 | CAY-10618 | NAMPT inhibitor |
| 93 | 83.78 | cp | BRD-K36737713 | AG-957 | Protein tyrosine kinase inhibitor |
| 88 | 84.55 | cp | BRD-A58955223 | sulforaphane | Antineoplastic |
| 87 | 84.56 | cp | BRD-K28120222 | parthenolide | NFkB pathway inhibitor |
| 79 | 85.8 | cp | BRD-K84895041 | BMY-45778 | IP1 prostacyclin receptor agonist |
| 72 | 86.73 | cp | BRD-K94325918 | kinetin-riboside | Apoptosis stimulant |
| 71 | 87.35 | cp | BRD-K03109492 | NSC-663284 | CDC inhibitor |
| 70 | 87.5 | cp | BRD-K67844266 | MLN-4924 | Nedd activating enzyme inhibitor |
| 67 | 87.76 | cp | BRD-K29113274 | ketoconazole | Sterol demethylase inhibitor |
| 65 | 87.98 | cp | BRD-K54233340 | dorsomorphin | AMPK inhibitor |
| 51 | 89.47 | cp | BRD-K81225797 | SCH-58261 | Adenosine receptor antagonist |
| 49 | 89.61 | cp | BRD-K59753975 | vindesine | Tubulin inhibitor |
| 45 | 90.17 | cp | BRD-K51730347 | diphencyprone | Immunostimulant |
| 42 | 90.53 | cp | BRD-K61829047 | 7b-cis | Exportin antagonist |
| 38 | 90.94 | cp | BRD-K40227168 | vinburnine | Adrenergic receptor antagonist |
| 36 | 91.65 | cp | BRD-K66792149 | quinoclamine | Algicide |
| 30 | 92.61 | cp | BRD-K56334280 | amonafide | Topoisomerase inhibitor |
| 28 | 92.79 | cp | BRD-K39983086 | loteprednol | Glucocorticoid receptor agonist |
| 13 | 97.29 | cp | BRD-A72596465 | GW-6471 | PPAR receptor antagonist |
| 5 | 98.89 | cp | BRD-K01555864 | dibenzoylmethane | Antineoplastic |

table S2. mRNA-miRNA relationship pairs

| miRNA | mRNA |
| --- | --- |
| miR-130ac | CBLB |
| miR-130ac | CBLB |
| miR-130ac | CBLB |
| miR-7 | CBLB |
| miR-7 | CBLB |
| miR-7 | CBLB |
| miR-9 | CBLB |
| miR-93 | CBLB |
| miR-96 | CBLB |
| miR-135ab | CBLB |
| miR-135ab | CBLB |
| miR-137 | CBLB |
| miR-138 | CBLB |
| miR-138 | CBLB |
| miR-139-5p | CBLB |
| miR-140 | CBLB |
| miR-140 | CBLB |
| miR-143 | CBLB |
| miR-143 | CBLB |
| miR-144 | CBLB |
| miR-144 | CBLB |
| miR-145 | CBLB |
| miR-146ac | CBLB |
| miR-146ac | CBLB |
| miR-148ab-3p | CBLB |
| miR-148ab-3p | CBLB |
| miR-148ab-3p | CBLB |
| miR-150 | CBLB |
| miR-150 | CBLB |
| miR-150 | CBLB |
| miR-150 | CBLB |
| miR-150 | CBLB |
| miR-150 | CBLB |
| miR-153 | CBLB |
| miR-155 | CBLB |
| miR-155 | CBLB |
| miR-15abc | CBLB |
| miR-15abc | CBLB |
| miR-17 | CBLB |
| miR-181abcd | CBLB |
| miR-181abcd | CBLB |
| miR-181abcd | CBLB |
| miR-182 | CBLB |
| miR-182 | CBLB |
| miR-183 | CBLB |
| miR-184 | CBLB |
| let-7 | CBLB |
| let-7 | CBLB |
| miR-187 | CBLB |
| miR-18ab | CBLB |
| miR-190 | CBLB |
| miR-191 | CBLB |
| miR-196abc | CBLB |
| miR-199ab-5p | CBLB |
| miR-199ab-5p | CBLB |
| miR-19ab | CBLB |
| miR-1ab | CBLB |
| miR-1ab | CBLB |
| miR-1ab | CBLB |
| miR-1ab | CBLB |
| miR-200bc | CBLB |
| miR-203 | CBLB |
| miR-203 | CBLB |
| miR-203 | CBLB |
| miR-203 | CBLB |
| miR-203 | CBLB |
| miR-203 | CBLB |
| miR-203 | CBLB |
| miR-204 | CBLB |
| miR-204 | CBLB |
| miR-205 | CBLB |
| miR-214 | CBLB |
| miR-216a | CBLB |
| miR-217 | CBLB |
| miR-218 | CBLB |
| miR-219-5p | CBLB |
| miR-22 | CBLB |
| miR-221 | CBLB |
| miR-221 | CBLB |
| miR-223 | CBLB |
| miR-223 | CBLB |
| miR-223 | CBLB |
| miR-122 | CBLB |
| miR-122 | CBLB |
| miR-122 | CBLB |
| miR-23abc | CBLB |
| miR-23abc | CBLB |
| miR-23abc | CBLB |
| miR-24 | CBLB |
| miR-24 | CBLB |
| miR-26ab | CBLB |
| miR-26ab | CBLB |
| miR-26ab | CBLB |
| miR-26ab | CBLB |
| miR-26ab | CBLB |
| miR-26ab | CBLB |
| miR-27abc | CBLB |
| miR-27abc | CBLB |
| miR-101 | CBLB |
| miR-101 | CBLB |
| miR-101 | CBLB |
| miR-29abcd | CBLB |
| miR-29abcd | CBLB |
| miR-30abcdef | CBLB |
| miR-31 | CBLB |
| miR-103a | CBLB |
| miR-124 | CBLB |
| miR-124 | CBLB |
| miR-124 | CBLB |
| miR-124 | CBLB |
| miR-124 | CBLB |
| miR-124 | CBLB |
| miR-338 | CBLB |
| miR-33a-3p | CBLB |
| miR-33a-3p | CBLB |
| miR-33ab | CBLB |
| miR-34ac | CBLB |
| miR-383 | CBLB |
| miR-383 | CBLB |
| miR-425 | CBLB |
| miR-128 | CBLB |
| miR-128 | CBLB |
| miR-129-5p | CBLB |
| miR-129-5p | CBLB |
| miR-129-5p | CBLB |
| miR-129-5p | CBLB |
| miR-129-5p | CBLB |
| miR-129-5p | CBLB |
| miR-490-3p | CBLB |
| miR-132 | RNF144A |
| miR-132 | RNF144A |
| miR-132 | RNF144A |
| miR-7 | RNF144A |
| miR-9 | RNF144A |
| miR-9 | RNF144A |
| miR-96 | RNF144A |
| miR-135ab | RNF144A |
| miR-138 | RNF144A |
| miR-142-3p | RNF144A |
| miR-143 | RNF144A |
| miR-143 | RNF144A |
| miR-146ac | RNF144A |
| miR-153 | RNF144A |
| miR-15abc | RNF144A |
| miR-15abc | RNF144A |
| miR-15abc | RNF144A |
| miR-17 | RNF144A |
| miR-18ab | RNF144A |
| miR-193 | RNF144A |
| miR-193 | RNF144A |
| miR-193 | RNF144A |
| miR-194 | RNF144A |
| miR-203 | RNF144A |
| miR-203 | RNF144A |
| miR-204 | RNF144A |
| miR-205 | RNF144A |
| miR-21 | RNF144A |
| miR-214 | RNF144A |
| miR-216b | RNF144A |
| miR-217 | RNF144A |
| miR-217 | RNF144A |
| miR-218 | RNF144A |
| miR-218 | RNF144A |
| miR-22 | RNF144A |
| miR-223 | RNF144A |
| miR-24 | RNF144A |
| miR-24 | RNF144A |
| miR-25 | RNF144A |
| miR-27abc | RNF144A |
| miR-27abc | RNF144A |
| miR-101 | RNF144A |
| miR-29abcd | RNF144A |
| miR-31 | RNF144A |
| miR-31 | RNF144A |
| miR-31 | RNF144A |
| miR-103a | RNF144A |
| miR-103a | RNF144A |
| miR-124 | RNF144A |
| miR-124 | RNF144A |
| miR-338 | RNF144A |
| miR-338 | RNF144A |
| miR-338 | RNF144A |
| miR-33a-3p | RNF144A |
| miR-33ab | RNF144A |
| miR-34ac | RNF144A |
| miR-34ac | RNF144A |
| miR-375 | RNF144A |
| miR-375 | RNF144A |
| miR-425 | RNF144A |
| miR-125a-5p | RNF144A |
| miR-10abc | RNF144A |
| miR-128 | RNF144A |
| miR-129-5p | RNF144A |
| miR-129-5p | RNF144A |
| miR-129-5p | RNF144A |

Code

logFoldChange=1

adjustP=0.05

library(limma)

rt=read.table("out.expr.txt",sep="\t",header=T,check.names=F)

rt=as.matrix(rt)

rownames(rt)=rt[,1]

exp=rt[,2:ncol(rt)]

dimnames=list(rownames(exp),colnames(exp))

rt=matrix(as.numeric(as.matrix(exp)),nrow=nrow(exp),dimnames=dimnames)

rt=avereps(rt)

rt=normalizeBetweenArrays(as.matrix(rt))

rt=log2(rt+1)

#differential

modType=c(rep("con",32),rep("treat",10))

design <- model.matrix(~0+factor(modType))

colnames(design) <- c("con","treat")

fit <- lmFit(rt,design)

cont.matrix<-makeContrasts(treat-con,levels=design)

fit2 <- contrasts.fit(fit, cont.matrix)

fit2 <- eBayes(fit2)

allDiff=topTable(fit2,adjust='fdr',number=200000)

write.table(allDiff,file="limmaTab.xls",sep="\t",quote=F)

diffSig <- allDiff[with(allDiff, (abs(logFC)>logFoldChange &P.Value < adjustP )), ]

write.table(diffSig,file="diff.xls",sep="\t",quote=F)

diffUp <- allDiff[with(allDiff, (logFC>logFoldChange & P.Value < adjustP )), ]

write.table(diffUp,file="up.xls",sep="\t",quote=F)

diffDown <- allDiff[with(allDiff, (logFC<(-logFoldChange) & P.Value < adjustP )), ]

write.table(diffDown,file="down.xls",sep="\t",quote=F)

hmExp=rt[rownames(diffSig),]

diffExp=rbind(id=colnames(hmExp),hmExp)

write.table(diffExp,file="heatmap.txt",sep="\t",quote=F,col.names=F)

pdf("vol.pdf")

xMax=max(abs(allDiff$logFC))

yMax=max(-log10(allDiff$P.Value))

plot(allDiff$logFC, -log10(allDiff$P.Value), xlab="log2FC",ylab="-log10(P.Value)",

main="Volcano",xlim=c(-xMax,xMax),ylim=c(0,yMax),yaxs="i",pch=20, cex=0.8)

diffSub=subset(allDiff, P.Value<adjustP & logFC>logFoldChange)

points(diffSub$logFC, -log10(diffSub$P.Value), pch=20, col="palevioletred1",cex=0.8)

diffSub=subset(allDiff, P.Value<adjustP & logFC<(-logFoldChange))

points(diffSub$logFC, -log10(diffSub$P.Value), pch=20, col="dodgerblue",cex=0.8)

abline(v=0,lty=2,lwd=3)

dev.off()

library(limma)

library(pheatmap)

library(gplots)

plotdata=read.table("heatmap.txt",sep="\t",header=T,check.names=F,row.names = 1)

up=read.table("up.xls",header=T,stringsAsFactors = F,sep = "\t")

down=read.table("down.xls",header=T,stringsAsFactors = F,sep = "\t")

annCol <- data.frame(Group = rep(c("Normal","Disease"),c(32,10)),

row.names = colnames(plotdata),

stringsAsFactors = F)

annRow <- data.frame(Direct = rep(c("Up","Down"),c(length(up$X),length(down$X))),

row.names = c(up$X,down$X),

stringsAsFactors = F)

annColors <- list("Group"=c("Normal"="blue",

"Disease"="red"),

"Direct"=c("Up"="yellow",

"Down"="green"))

plotdata <- plotdata[c(up$X,down$X),]

plotdata <- t(scale(t(plotdata)))

plotdata[plotdata > 5] <- 5

plotdata[plotdata < -5] <- -5

pdf(file="heatmap.pdf",width = 8,height = 8)

pheatmap(plotdata,

scale = "none",

annotation_row=annRow,

annotation_col=annCol,

annotation_colors = annColors,

color = colorRampPalette(c("navy", "white", "palevioletred1"))(20),

#color = greenred(64),

fontsize_row=7,

fontsize_col=5,

fontsize=9,

cluster_cols = FALSE,

cluster_rows = FALSE,

show_colnames = F,

show_rownames = F)

dev.off()

library("org.Hs.eg.db")

rt=read.table("gene.txt",sep="\t",check.names=F,header=T)

genes=as.vector(rt[,1])

entrezIDs <- mget(genes, org.Hs.egSYMBOL2EG, ifnotfound=NA)

entrezIDs <- as.character(entrezIDs)

out=cbind(rt,entrezID=entrezIDs)

write.table(out,file="id.txt",sep="\t",quote=F,row.names=F)

library("clusterProfiler")

library("org.Hs.eg.db")

library("enrichplot")

library("ggplot2")

rt=read.table("id.txt",sep="\t",header=T,check.names=F)

rt=rt[is.na(rt[,"entrezID"])==F,]

gene=rt$entrezID

kk <- enrichGO(gene = gene,

OrgDb = org.Hs.eg.db,

pvalueCutoff =0.05,

qvalueCutoff = 0.05,

ont="all",

readable =T)

write.table(kk,file="GO.txt",sep="\t",quote=F,row.names = F)

pdf(file="GObarplot.pdf",width = 10,height = 13)

barplot(kk, drop = TRUE, showCategory =10,split="ONTOLOGY",label_format=100) + facet_grid(ONTOLOGY~., scale='free')

dev.off()

pdf(file="GObubble.pdf",width = 10,height = 15)

dotplot(kk,showCategory = 10,split="ONTOLOGY") + facet_grid(ONTOLOGY~., scale='free')

dev.off()

library("clusterProfiler")

library("org.Hs.eg.db")

library("enrichplot")

library("ggplot2")

rt=read.table("id.txt",sep="\t",header=T,check.names=F)

rt=rt[is.na(rt[,"entrezID"])==F,]

gene=rt$entrezID

kk <- enrichKEGG(gene = gene, organism = "hsa", pvalueCutoff =0.05, qvalueCutoff =0.05)

write.table(kk,file="KEGGId.txt",sep="\t",quote=F,row.names = F)

pdf(file="KEGGbarplot.pdf",width = 10,height = 10)

barplot(kk, drop = TRUE, showCategory = 30,label_format=100)

dev.off()

pdf(file="KEGGbubble.pdf",width = 10,height = 10)

dotplot(kk, showCategory = 30)

dev.off()

library(tidyverse)

library(glmnet)

source('msvmRFE.R')

library(VennDiagram)

library(sigFeature)

library(e1071)

library(caret)

library(randomForest)

train<-read.table("ARGexp.txt",row.names = 1,as.is = F,header = T)

train[1:4,1:4]

x <- as.matrix(train[,-1])

(y <- ifelse(train$group == "NR", 0,1))

x[1:4,1:4]

#library(glmnet)

set.seed(800)

fit = glmnet(x, y, family = "binomial", alpha = 1, lambda = NULL)

plot(fit, xvar = "dev", label = TRUE)

cvfit = cv.glmnet(x, y,

nfold=10,

family = "binomial", type.measure = "class")

plot(cvfit)

cvfit$lambda.min

myCoefs <- coef(cvfit, s="lambda.min");

lasso_fea <- myCoefs@Dimnames[[1]][which(myCoefs != 0 )]

(lasso_fea <- lasso_fea[-1])

write.csv(lasso_fea,"feature_lasso.csv")

predict <- predict(cvfit, newx = x[1:nrow(x),], s = "lambda.min", type = "class")

table(predict,y)

input <- train

set.seed(20)

svmRFE(input, k = 5, halve.above = 100)

nfold = 5

nrows = nrow(input)

folds = rep(1:nfold, len=nrows)[sample(nrows)]

folds = lapply(1:nfold, function(x) which(folds == x))

results = lapply(folds, svmRFE.wrap, input, k=5, halve.above=100)

top.features = WriteFeatures(results, input, save=F)

head(top.features)

write.csv(top.features,"feature_svm.csv")

featsweep = lapply(1:10, FeatSweep.wrap, results, input)

featsweep

no.info = min(prop.table(table(input[,1])))

errors = sapply(featsweep, function(x) ifelse(is.null(x), NA, x$error))

PlotErrors(errors, no.info=no.info)

Plotaccuracy(1-errors,no.info=no.info)

which.min(errors)

(myoverlap <- intersect(lasso_fea, top.features[1:which.min(errors), "FeatureName"]))

summary(lasso_fea%in%top.features[1:which.min(errors), "FeatureName"])

pdf("C_lasso_SVM_venn.pdf", width = 5, height = 3)

grid.newpage()

venn.plot <- venn.diagram(list(LASSO = lasso_fea,

SVM_RFE = as.character(top.features[1:which.min(errors),"FeatureName"])), NULL,

fill = c("#E31A1C","#E7B800"),

alpha = c(0.5,0.5), cex = 4, cat.fontface=3,

category.names = c("LASSO", "SVM_RFE"),

main = "Overlap")

grid.draw(venn.plot)

dev.off()

library(randomForest)

exp <- read.table("symbol.txt",sep = "\t",row.names = 1,check.names = F,stringsAsFactors = F,header = T)

exp <- as.matrix(exp)

exp<-log2(exp+1)

group <- read.table("group.txt",sep = "\t",row.names = 1,check.names = F,stringsAsFactors = F,header = T)

com_sam <- intersect(colnames(exp),rownames(group))

exp <- exp

set.seed(200)

rownames(exp) <- gsub("-","_",rownames(exp))

dat <- cbind.data.frame(t(exp[,com_sam]),group[com_sam,,drop = F])

dat$Group <- ifelse(dat$Group == "normal",0,1)

group <- factor(group$Group)

model_RF <- randomForest(Group ~ .,

data = dat,

ntree = 1000,

nPerm = 50,

mtry = floor(sqrt(ncol(dat)-1)),

proximity = T,

importance = T)

varImpPlot(model_RF)

imp <- as.data.frame(importance(model_RF, type = 1))

sel_var <- imp[order(imp$`%IncMSE`,decreasing = T),,drop = F]

imp.ori <- as.data.frame(importance(model_RF))

write.table(imp.ori,"importance of variables.txt",sep = "\t",row.names = T,col.names = NA,quote = F)

source("CIBERSORT.R")

results=CIBERSORT("ref.txt", "out.expr.txt", perm=100, QN=TRUE)

input="CIBERSORT-Results.txt"

outpdf="barplot.pdf"

data <- read.table(input,header=T,sep="\t",check.names=F,row.names=1)

data=t(data)

col=rainbow(nrow(data),s=0.7,v=0.7)

pdf(outpdf,height=10,width=25)

par(las=1,mar=c(8,4,4,15))

a1 = barplot(data,col=col,yaxt="n",ylab="Relative Percent",xaxt="n")

a2=axis(2,tick=F,labels=F)

axis(2,a2,paste0(a2*100,"%"))

axis(1,a1,labels=F)

par(srt=65,xpd=T);text(a1,-0.02,colnames(data),adj=1.1,cex=1.1);par(srt=0)

ytick2 = cumsum(data[,ncol(data)])

ytick1 = c(0,ytick2[-length(ytick2)])

legend(par('usr')[2]*0.98,par('usr')[4],legend=rownames(data),col=col,pch=15,bty="n",cex=1.3)

dev.off()

rt=read.table("CIBERSORT-Results.txt",sep="\t",header=T,row.names=1,check.names=F)

library(corrplot)

pdf("corHeatmap.pdf",height=13,width=13)

corrplot(corr=cor(rt),

method = "color",

order = "hclust",

tl.col="black",

addCoef.col = "black",

number.cex = 1,

col=colorRampPalette(c("blue", "white", "red"))(50),

)

dev.off()

library(vioplot)

normal=32

tumor=10

rt=read.table("CIBERSORT-Results.txt",sep="\t",header=T,row.names=1,check.names=F)

pdf("vioplot.pdf",height=8,width=15)

par(las=1,mar=c(10,6,3,3))

x=c(1:ncol(rt))

y=c(1:ncol(rt))

plot(x,y,

xlim=c(0,64),ylim=c(min(rt),max(rt)+0.02),

main="",xlab="", ylab="Fraction",

pch=21,

col="white",

xaxt="n")

text(seq(1,64,3),-0.05,xpd = NA,labels=colnames(rt),cex = 1,srt = 45,pos=2)

for(i in 1:ncol(rt)){

normalData=rt[1:normal,i]

tumorData=rt[(normal+1):(normal+tumor),i]

vioplot(normalData,at=3*(i-1),lty=1,add = T,col = 'blue')

vioplot(tumorData,at=3*(i-1)+1,lty=1,add = T,col = 'red')

wilcoxTest=wilcox.test(normalData,tumorData)

p=round(wilcoxTest$p.value,3)

mx=max(c(normalData,tumorData))

lines(c(x=3*(i-1)+0.2,x=3*(i-1)+0.8),c(mx,mx))

text(x=3*(i-1)+0.5,y=mx+0.02,labels=ifelse(p<0.001,paste0("p<0.001"),paste0("p=",p)),cex = 0.8)

}

dev.off()

library(ggplot2)

library(ggpubr)

library(SimDesign)

library(cowplot)

library(dplyr)

library(GSVA)

library(limma)

library(stringr)

jco <- c("#2874C5","#EABF00","#868686","#C6524A","#80A7DE")

expr <- read.table("out.expr.txt",sep = "\t",row.names = 1,check.names = F,stringsAsFactors = F,header = T)

gene <- read.table("genelist.txt",sep = "\t",row.names = NULL,check.names = F,stringsAsFactors = F,header = F)

ciber <- read.table("CIBERSORT-Results.txt",sep = "\t",row.names = 1,check.names = F,stringsAsFactors = F,header = T)

for (i in gene$V1) {

message(paste0("analysis of ",i," starts..."))

subexpr <- as.numeric(expr[i,])

names(subexpr) <- colnames(expr)

lsam <- names(subexpr[subexpr < median(subexpr)])

hsam <- names(subexpr[subexpr >= median(subexpr)])

dat <- as.numeric(expr[i,]); names(dat) <- colnames(expr)

comsam <- intersect(names(dat), rownames(ciber))

tmp1 <- dat[comsam]

tmp2 <- ciber[comsam,]

var <- colnames(ciber)

data <- data.frame(var)

for (j in 1:length(var)){

test <- cor.test(as.numeric(tmp2[,j]),tmp1,method = "spearman")

data[j,2] <- test$estimate

data[j,3] <- test$p.value

}

names(data) <- c("symbol","correlation","pvalue")

data <- as.data.frame(na.omit(data))

data %>%

filter(pvalue <0.05) %>%

ggplot(aes(correlation,forcats::fct_reorder(symbol,correlation))) +

geom_segment(aes(xend=0,yend=symbol)) +

geom_point(aes(col=pvalue,size=abs(correlation))) +

scale_colour_gradientn(colours=c("#7fc97f","#984ea3")) +

scale_size_continuous(range =c(2,8)) +

theme_minimal() +

ylab(NULL)

ggsave(paste0("correlation between cibersort and expression of ", i,".pdf"),width = 8,height = 6)

}

library(ggplot2)

library(stringr)

a_1 <- read.table("out.expr.txt",header = T,row.names = 1,sep = "\t", quote = "",fill = T,check.names=F)

a_1 <- log(a_1+2)

dim(a_1)

head(a_1[,1:3])

a_2 <- as.data.frame(t(a_1))

dim(a_2)

head(a_2[,1:3])

a_3 <- a_1

a_3$Id <- rownames(a_3)

dim(a_3)

head(a_3[,1:3])

b_1 <- read.table("Immunomodulator_and_chemokines.txt",header = T,sep = "\t", quote = "",fill = T)

dim(b_1)

head(b_1)

b_2 <- b_1[b_1$type == "receptor",]

dim(b_2)

head(b_2)

data1 <- dplyr::inner_join(b_2,a_3,by="Id")

dim(data1)

head(data1[,1:6])

data2 <- a_2[,c("CBLB","JADE2","RNF144A",data1$Id)]

dim(data2)

head(data2[,1:5])

library(Hmisc)

CorMatrix <- function(cor,p) {

ut <- upper.tri(cor)

data.frame(row = rownames(cor)[row(cor)[ut]] ,

column = rownames(cor)[col(cor)[ut]],

cor =(cor)[ut],

p = p[ut] )

}

res <- rcorr(as.matrix(data2),type = "pearson")

result_1 <- CorMatrix(res$r, res$P)

head(result_1)

dim(result_1)

result_2 <- result_1[result_1$row == "CBLB" |result_1$row == "JADE2"|result_1$row == "RNF144A",]

dim(result_2)

head(b_2)

b_2$column <- b_2$Id

head(b_2)

result_3 <- dplyr::inner_join(result_2,b_2,by="column")

dim(result_3)

result1 <- result_3[,1:4]

head(result1)

dim(result1)

result1$Regulation <- result1$cor

result1[,5][result1[,5] > 0] <- c("postive")

result1[,5][result1[,5] < 0] <- c("negative")

head(result1)

colnames(result1) <- c("gene", "immuneGene", "cor", "pvalue", "Regulation")

write.table(result1,file="receptor.xls",sep="\t",quote=F,col.names=T,row.names = F)

a1 <- read.table("receptor.xls",header = T,sep = "\t", quote = "",fill = T)

head(a1)

data2 <- a1

library(ggpubr)

data2$pvalue <- ifelse(data2$pvalue < 0.05,

ifelse(data2$pvalue < 0.01,"**","*"),

"")

data2$pvalue[1:20]

data2$type <- data2$cor

summary(data2)

data3 <- data2[order(data2$immuneGene,data2$cor),]

head(data3)

dim(data3)

data4 <- data3[data3$pvalue < 0.05,]

dim(data4)

summary(data4)

p <- ggplot(data4,aes(x=gene,y=immuneGene)) +

geom_point(aes(colour = cor, size=pvalue)) +

labs(x="",y="receptor")

p <- p + scale_colour_gradient2(low = "blue", high = "red", mid = "white",

midpoint = 0, limit = c(-1, 1), space = "Lab",

name="Pearson\nCorrelation")

p <- p + theme_bw() +

theme(panel.grid.major = element_blank(), panel.grid.minor = element_blank()) +

theme(axis.text=element_text(size = 15)) +

theme(axis.text.x=element_text(colour = "black",angle=0,hjust=0.5,size = 15)) +

theme(axis.text.y=element_text(colour = "black", vjust=0,size = 15)) +

theme(axis.title =element_text(size = 20)) +

theme(text = element_text(size = 15))

p+rotate_x_text(45)

ggsave("receptor.pdf")

library(ggpubr)

pFilter=0.99

rt=read.table("ARGexp.txt",sep="\t",header=T,row.names=1,check.names=F)

rt=log2(rt+1)

data=rt

Type=read.table("cluster.Immunity.txt",sep="\t",check.names=F,row.names=1,header=F)

Type=Type[row.names(data),]

colnames(Type)=c("cluster","Subtype")

outTab=data.frame()

data=cbind(data,Type)

for(i in colnames(data[,1:(ncol(data)-2)])){

rt1=data[,c(i,"Subtype")]

colnames(rt1)=c("expression","Subtype")

ksTest<-kruskal.test(expression ~ Subtype, data = rt1)

pValue=ksTest$p.value

if(pValue<pFilter){

outTab=rbind(outTab,cbind(rt1,gene=i))

print(pValue)

}

}

write.table(outTab,file="data.txt",sep="\t",row.names=F,quote=F)

data=read.table("data.txt",sep="\t",header=T,check.names=F)

data$Subtype=factor(data$Subtype, levels=c("Normal","Disease"))

p=ggboxplot(data, x="gene", y="expression",color = "grey",fill = "Subtype",

ylab="Expression",

xlab="",

palette =c("skyblue","pink") )

p=p+rotate_x_text(45)

p

pdf(file="boxplot.pdf",width=12,height=4)

p+stat_compare_means(aes(group=Subtype),symnum.args=list(cutpoints = c(0, 0.001, 0.01, 0.05, 1), symbols = c("***", "**", "*", "ns")),label = "p.signif",method="wilcox")

dev.off()

library(dplyr)

library(ggplot2)

data %>%

filter(Subtype %in% c("Normal","Disease")) %>%

ggplot(aes(x= gene, y= expression, fill = Subtype, color = Subtype))+

geom_boxplot(alpha=0.3)+

scale_fill_manual(name= "Subtype", values = c("deepskyblue", "hotpink"))+

scale_color_manual(name = "Subtype", values = c("dodgerblue", "plum3"))+

theme_bw()+labs(x="", y="Expression")+

theme(axis.text.x = element_text( vjust = 1,size = 12, hjust = 1,colour = "black"),legend.position="top")+

rotate_x_text(45)+stat_compare_means(aes(group=Subtype),symnum.args=list(cutpoints = c(0, 0.001, 0.01, 0.05, 1), symbols = c("***", "**", "*", "ns")),label = "p.signif",method="wilcox")

library(ggplot2)

library(stringr)

a_1 <- read.table("symbol.txt",header = T,row.names = 1,sep = "\t", quote = "",fill = T,check.names=F)

a_1=log2(a_1+1)

dim(a_1)

head(a_1[,1:3])

a_2 <- as.data.frame(t(a_1))

dim(a_2)

head(a_2[,1:3])

a_3 <- a_1

a_3$Id <- rownames(a_3)

dim(a_3)

head(a_3[,1:3])

b_1 <- read.table("111.txt",header = T,sep = "\t", quote = "",fill = T)

dim(b_1)

head(b_1)

b_2 <- b_1[b_1$type == "Disease",]

dim(b_2)

head(b_2)

data1 <- dplyr::inner_join(b_2,a_3,by="Id")

dim(data1)

head(data1[,1:6])

data2 <- a_2[,c("CBLB", "JADE2","RNF144A",data1$Id)]

dim(data2)

head(data2[,1:5])

library(Hmisc)

CorMatrix <- function(cor,p) {

ut <- upper.tri(cor)

data.frame(row = rownames(cor)[row(cor)[ut]] ,

column = rownames(cor)[col(cor)[ut]],

cor =(cor)[ut],

p = p[ut] )

}

res <- rcorr(as.matrix(data2),type = "pearson")

result_1 <- CorMatrix(res$r, res$P)

head(result_1)

dim(result_1)

result_2 <- result_1[result_1$row == "CBLB" |result_1$row == "JADE2" |result_1$row == "RNF144A",]

dim(result_2)

head(b_2)

b_2$column <- b_2$Id

head(b_2)

result_3 <- dplyr::inner_join(result_2,b_2,by="column")

dim(result_3)

result1 <- result_3[,1:4]

head(result1)

dim(result1)

result1$Regulation <- result1$cor

result1[,5][result1[,5] > 0] <- c("postive")

result1[,5][result1[,5] < 0] <- c("negative")

head(result1)

colnames(result1) <- c("gene", "immuneGene", "cor", "pvalue", "Regulation")

write.table(result1,file="SAP.xls",sep="\t",quote=F,col.names=T,row.names = F)

a1 <- read.table("SAP.xls",header = T,sep = "\t", quote = "",fill = T)

head(a1)

data2 <- a1

library(ggpubr)

data2$pvalue <- ifelse(data2$pvalue < 0.05,

ifelse(data2$pvalue < 0.01,"**","*"),

"")

data2$pvalue[1:20]

data2$type <- data2$cor

summary(data2)

data3 <- data2[order(data2$immuneGene,data2$cor),]

head(data3)

dim(data3)

data4 <- data3[data3$pvalue < 0.05,]

dim(data4)

summary(data4)

p <- ggplot(data4,aes(x=gene,y=immuneGene)) +

geom_point(aes(colour = cor, size=pvalue)) +

labs(x="",y="SAP genes")

p <- p + scale_colour_gradient2(low = "blue", high = "red", mid = "white",

midpoint = 0, limit = c(-1, 1), space = "Lab",

name="Pearson\nCorrelation")

p <- p + theme_bw() +

theme(panel.grid.major = element_blank(), panel.grid.minor = element_blank()) +

theme(axis.text=element_text(size = 15)) +

theme(axis.text.x=element_text(colour = "black",angle=0,hjust=0.5,size = 15)) +

theme(axis.text.y=element_text(colour = "black", vjust=0,size = 15)) +

theme(axis.title =element_text(size = 20)) +

theme(text = element_text(size = 15))

p+rotate_x_text(45)

ggsave("SAP.pdf")

library(ggplot2)

library(ggExtra)

rt=read.table("symbol.txt",sep="\t",header=T,check.names=F,row.names = 1)

rt=log2(rt+1)

dat<-as.data.frame(t(rt))

corr_eqn <- function(x,y,digits=3) {

test <- cor.test(x,y,type="pearson")

paste(paste0("n = ",length(x)),

paste0("r = ",round(test$estimate,digits),"(Pearson)"),

paste0("p.value= ",round(test$p.value,digits)),

sep = ", ")

}

gene<-as.numeric(dat$CBLB)

imucell<-dat$FLT3

corr_eqn(gene,imucell)

gg<-ggplot(dat, aes(x=gene, y=imucell)) +

geom_point(color = "black") +

geom_smooth(method="loess", se=F,color="blue") +

labs(

y="FLT3",

x="CBLB",

title="Scatterplot")+

labs(title = paste0(corr_eqn(gene,imucell)))+

theme_bw()

gg

gg2 <- ggMarginal(gg, type="density")

gg2 <- ggMarginal(gg, type="density",xparams = list(fill ="orange"),

yparams = list(fill ="skyblue"))
